# Structure-Guided Design of Benzothiazole and Benzimidazole-Based Urea Derivatives Curtailing Oncogenic Signaling via Concurrent Inhibition of VEGFR-2, EGFR, and c‑Met

**DOI:** 10.1021/acsomega.5c10972

**Published:** 2026-01-21

**Authors:** Sadia Shaheen, Arshma Siddique, Ali Iftikhar, Amir Faisal, Hafiz M. Rehman, Ghulam Murtaza, Ayesha Tahir, Anees Saeed, Abbas Hassan, Umer Rashid

**Affiliations:** † Department of Chemistry, COMSATS University Islamabad, Abbottabad Campus, 22060 Abbottabad, Pakistan; ‡ Department of Life Sciences, SBA School of Science and Engineering, 66687 Lahore University of Management Sciences, Lahore Cantt., Lahore 54792, Pakistan; § School of Biochemistry and Biotechnology, 66906 University of the Punjab, Lahore 54590, Punjab, Pakistan; ∥ Department of Pharmacy, COMSATS University Islamabad, Lahore Campus, Lahore 54000, Pakistan; ⊥ Department of Chemistry, College of Science, 11239 United Arab Emirates University, Al Ain, 15551, Abu Dhabi, United Arab Emirates

## Abstract

Receptor tyrosine kinases (RTKs), including VEGFR-2, EGFR, and c-MET, have been recognized as promising oncogenic targets in tumor progression, invasion, and metastasis. Developing multitarget inhibitors that block these kinases simultaneously offers a powerful strategy to suppress angiogenesis and oncogenic signaling, while potentially minimizing adverse effects. A new series of benzothiazole- and benzimidazole-based urea derivatives was designed rationally through scaffold modification and linker optimization to enhance multikinase inhibition. Moreover, *in vitro* evaluation of the newly synthesized series revealed that compounds **6a**–**c**, **7a**, **12a**, **17**, and **18** exhibited multitarget inhibitory potential. Additionally, **11b**, **12a**, **17**, and **18** showed the best antiproliferative potential against MCF7 and A549 cells, as indicated by the antiproliferative assay. While compounds **6b**, **7a**, **17**, and **18** demonstrated negligible cytotoxicity against normal HEK-293 cells, with IC_50_ values exceeding 100 μM (>100 μM). Furthermore, the antiangiogenic efficacy of **11b**, **12a**, **17**, and **18** was validated through CAM assays, which markedly suppressed neovascularization. Molecular docking revealed efficient occupation of **6b**, **7a**, **12a**, **17,** and **18** with key binding pockets across VEGFR-2, EGFR, and c-Met. The 200 ns molecular dynamics (MD) simulations confirmed the stability of the **4ASD–6b** complex with enhanced flexibility compared to sorafenib. Collectively, these findings establish benzothiazole, benzimidazole, and quinoline-based urea hybrids as promising leads with enhanced multikinase selectivity and reduced toxicity compared to existing inhibitors, offering strong therapeutic potential in angiogenesis-driven cancers.

## Graphical abstract

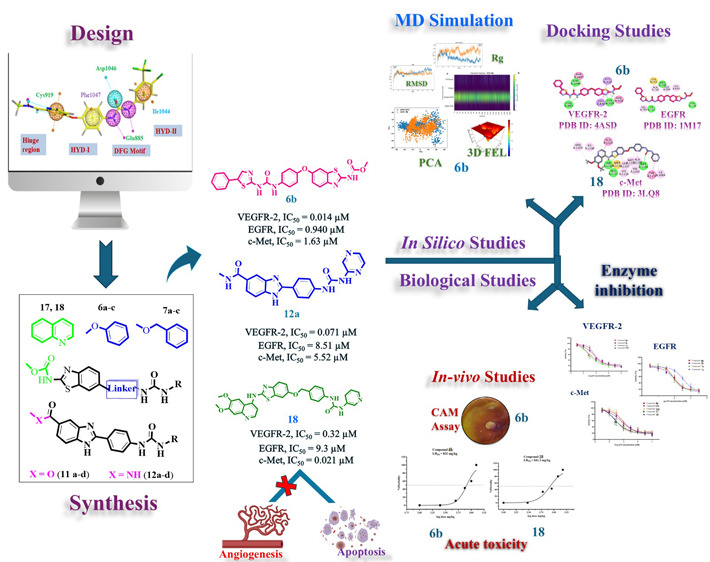

## Introduction

Globally, cancer ranks as the second most prevalent cause of death.^1,2^ In 2022, an estimated 20 million new cancer cases and 10 million deaths were reported globally, with annual cases projected to rise by 77% to reach 35 million by 2050.^3^ Cancer results from genetic and epigenetic changes that induce uncontrolled proliferation of cells, frequently triggered by environmental and lifestyle exposures. Currently, chemotherapeutic medications represent an important option for cancer treatment, together with surgery and radiotherapy, but are plagued by poor selectivity, high toxicity, and quick resistance.^4,5^ To address these drawbacks, targeted therapies have been created to take advantage of tumor cell-specific weaknesses in proliferative, survival, and angiogenic signaling cascades.^6^

Receptor tyrosine kinases (RTKs) are cell surface receptors, transducing extracellular signals to initiate signaling pathways regulating proliferation, survival, angiogenesis, and metastasis under both normal and pathological conditions.^7^ The vascular endothelial growth factor receptor (VEGFR-2), human epidermal growth factor receptor (EGFR), and human mesenchymal to epithelial transition receptor (c-MET) are members of the receptor tyrosine kinase family. Aberrant activation of these three RTKs (VEGFR, EGFR, and c-Met) is strongly recognized as a key therapeutic target in cancer treatment.^8,9^

VEGF enhances cell permeability, angiogenesis, and tumor progression,^10,11^ through VEGFR-1, VEGFR-2, and VEGFR-3, with VEGFR-2 being the key therapeutic target due to its overexpression in tumor-associated endothelial cells.^12–14^ Moreover, VEGFR-2 also blocks the proliferation of liver stem cells (LSCs) in the injury process.^15^ VEGFR-2 inhibitors are classified as type I (e.g., Sunitinib),^16^ type II (e.g., Sorafenib), and type III (e.g., Fruquintinib), based on their binding modes. Notably, type II inhibitors that stabilize the inactive “DFG-out” conformation exhibit superior potency and selectivity^17–19^ (Figure 1).

**Figure 1:**
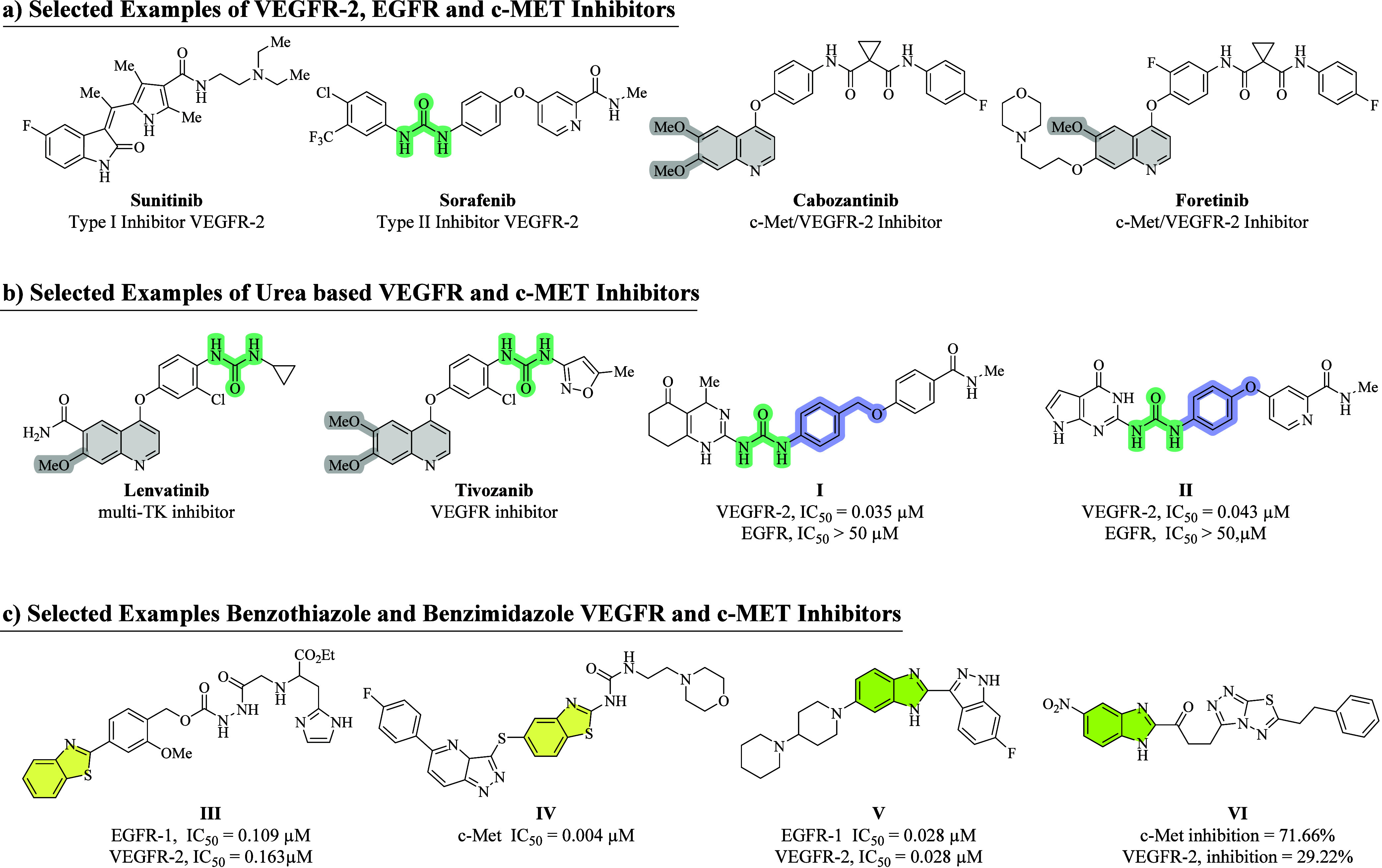
(a) Selected marketed VEGFR-2, EGFR, and c-Met inhibitors, (b) urea-based VEGFR, EGFR, VEGFR, and c-Met inhibitors, and (c) reported benzothiazole/benzimidazole as c-Met, EGFR, and VEGFR-2 inhibitors.^34–37^

Epidermal growth factor receptor EGFR (ErbB1/HER1), frequently mutated or overexpressed in many solid tumors, and its aberrant activation trigger downstream signaling cascades that promote cell survival, proliferation, and metastasis.^20^ Its activation causes VEGF upregulation, thereby indirectly stimulating VEGFR-2-mediated angiogenesis.^21^ Moreover, dysregulation of Death-associated protein kinase 2 (DAPK2), a key regulator of apoptosis, autophagy, and mitochondrial homeostasis, causes EGFR-TKI resistance.^22^ Therefore, inhibition of EGFR with strategies that address DAPK2-related resistance mechanisms can effectively suppress both proliferative and angiogenic pathways and may be essential for designing more effective multitarget antiangiogenic therapies.^22,23^

c-Met, the receptor for hepatocyte growth factor (HGF), is commonly dysregulated in variant human cancers and drives tumor growth, angiogenesis, and metastasis.^24^ Its signaling synergizes with VEGFR-2, making combined inhibition of c-Met, VEGFR-2, and EGFR a rational multitarget strategy to overcome resistance.^25^ Clinically, Gefitinib targets EGFR/VEGFR-2, while Cabozantinib and Foretinib act as dual VEGFR-2/c-Met inhibitors used for advanced Renal Cell Carcinoma^26,27^ (Figure 1).

The urea moiety has received considerable attention as a key pharmacophore in the design of targeted anticancer medications due to its strong and specific binding with tyrosine kinases.^28^ Urea-based drugs like Sorafenib, Lenvatinib, and Tivozanib are FDA-approved VEGFR-2 inhibitors.^29–31^ Our research group recently reported bicyclic 2-aminopyrimidine-urea hybrid VEGFR-2 inhibitors. Among all, compounds **I** and **II** emerged as potent and selective VEGFR-2 inhibitors, with IC_50_ values of 0.035 and 0.043 μM, respectively, comparable to Sorafenib (IC_50_ = 0.027 μM^32^; Figure 1). The benzothiazole moiety possesses extensive pharmacological effects against various cancers.^33^ The reported benzothiazole-based derivative **III** acts as a dual inhibitor of VEGFR-2/EGFR with IC_50_ = 0.163 and 0.109 μM^34^ while **IV** acts as a c-Met inhibitor with an IC_50_ value of 0.004 μM (Figure 1). Similarly, benzimidazole is a chemically active moiety present in various reported VEGFR-2, EGFR, and c-Met inhibitors.^35^ The reported benzimidazole-based compound **V** was recognized as a dual inhibitor of EGFR/VEGFR-2, with an IC_50_ value of 0.028 μM toward both EGFR and VEGFR-2,^36^ while **VI** shows dual VEGFR-2/c-Met inhibition percentage of 29.22% and 71.66%, respectively (Figure 1).^37^

In the design rationale, fragment merging was used as a deliberate structure-based approach to integrate pharmacophoric elements from multiple known kinase inhibitors (e.g., sorafenib, cabozantinib, and EGFR-active heteroaryl scaffolds). Docking analysis within VEGFR-2 (PDB 4ASD), EGFR (PDB 1M17), and c-Met (PDB 3LQ8) ATP-binding pockets revealed that three core fragments consistently acted as key interaction motifs: (1) urea linker, forming conserved H-bond interactions with the hinge region/Glu–Asp residues; (2) planar heteroaryl head groups (benzothiazole/benzimidazole/pyrazine/thiazole), engaging the gatekeeper region through π–π contacts and polar anchoring; and (3) terminal hydrophobic/aromatic fragments, filling the allosteric/back pocket to enhance multikinase affinity.

Using fragment merging, these complementary fragments were combined into a single hybrid scaffold, so that every structural element fulfills a distinct binding role across the three kinases. This merging enabled us to align urea-mediated hinge recognition, heteroaryl-driven ATP-pocket anchoring, and hydrophobic tail occupancy within the same molecular framework, resulting in compounds **6a–c**, **7a–c**, **11a–d**, and **12a**–**d** capable of simultaneously inhibiting VEGFR-2, EGFR, and c-Met (Figure 2).

**Figure 2:**
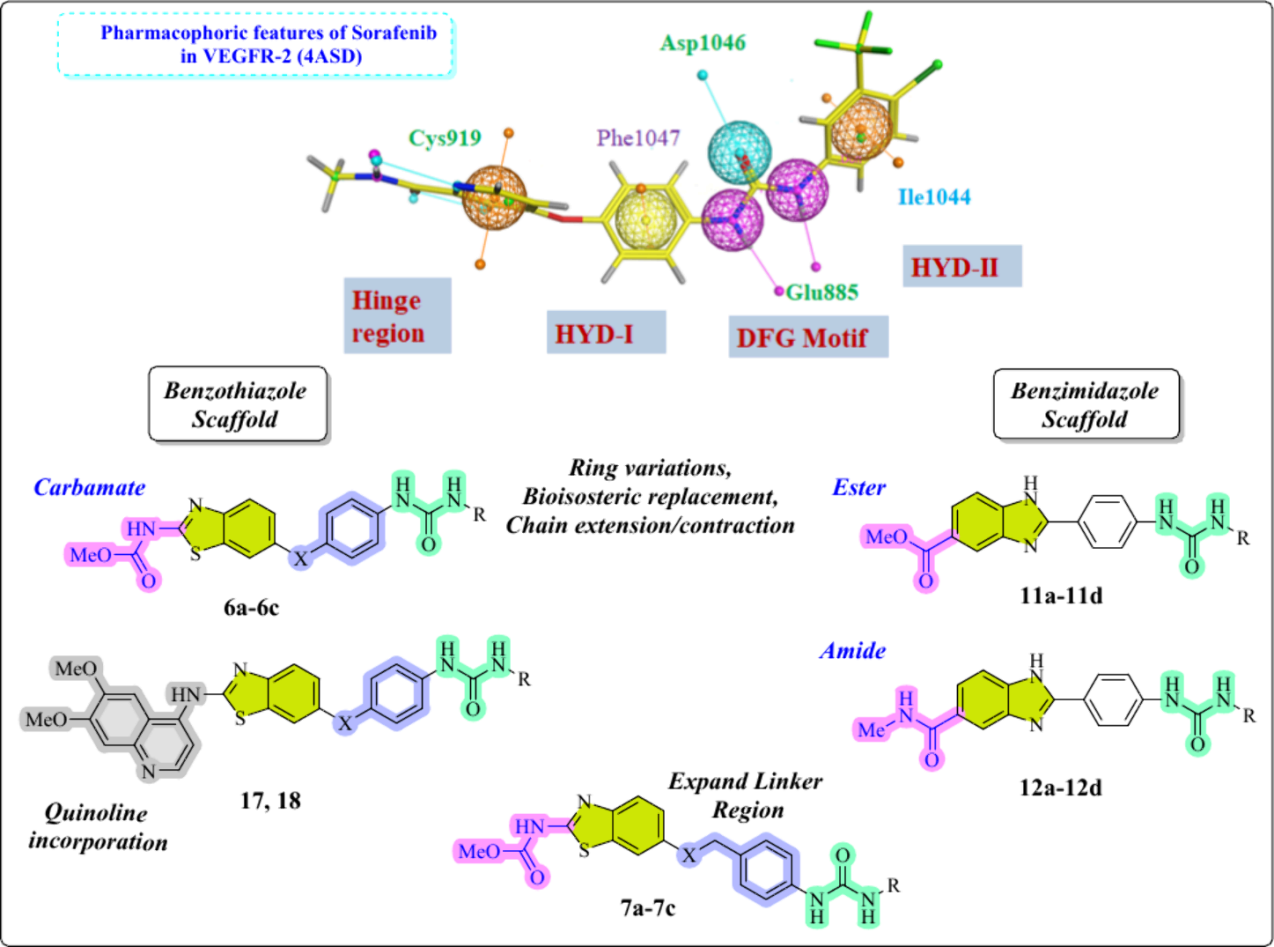
Design strategy for new target compounds against VEGFR-2, c-Met, and EGFR.

Compared with our earlier bicyclic-2-aminopyrimidine-based urea derivatives (IC_5__0_: 0.035–0.043 μM, VEGFR-2 selective),^32^ current design introduces structural diversification at the scaffold and functional group levels. Specifically, we retained the urea pharmacophore and linkers but expanded the chemical space by incorporating carbamates, esters, extended amide, and quinoline, offering a promising framework to inhibit angiogenesis, tumor proliferation, and metastasis simultaneously.

## Results and Discussion

### Chemistry

This study centers on the strategic incorporation of benzothiazole and benzimidazole scaffolds in the synthesis of a new series of multitarget kinase (VEGFR-2, c-Met, and EGFR) inhibitors, intending to investigate their biological relevance. Different substitution patterns were incorporated into these scaffolds to create a series of compounds for anticancer evaluation. In the synthetic procedures outlined in Schemes 1–3, target compounds were successfully prepared.

**Scheme 1:**
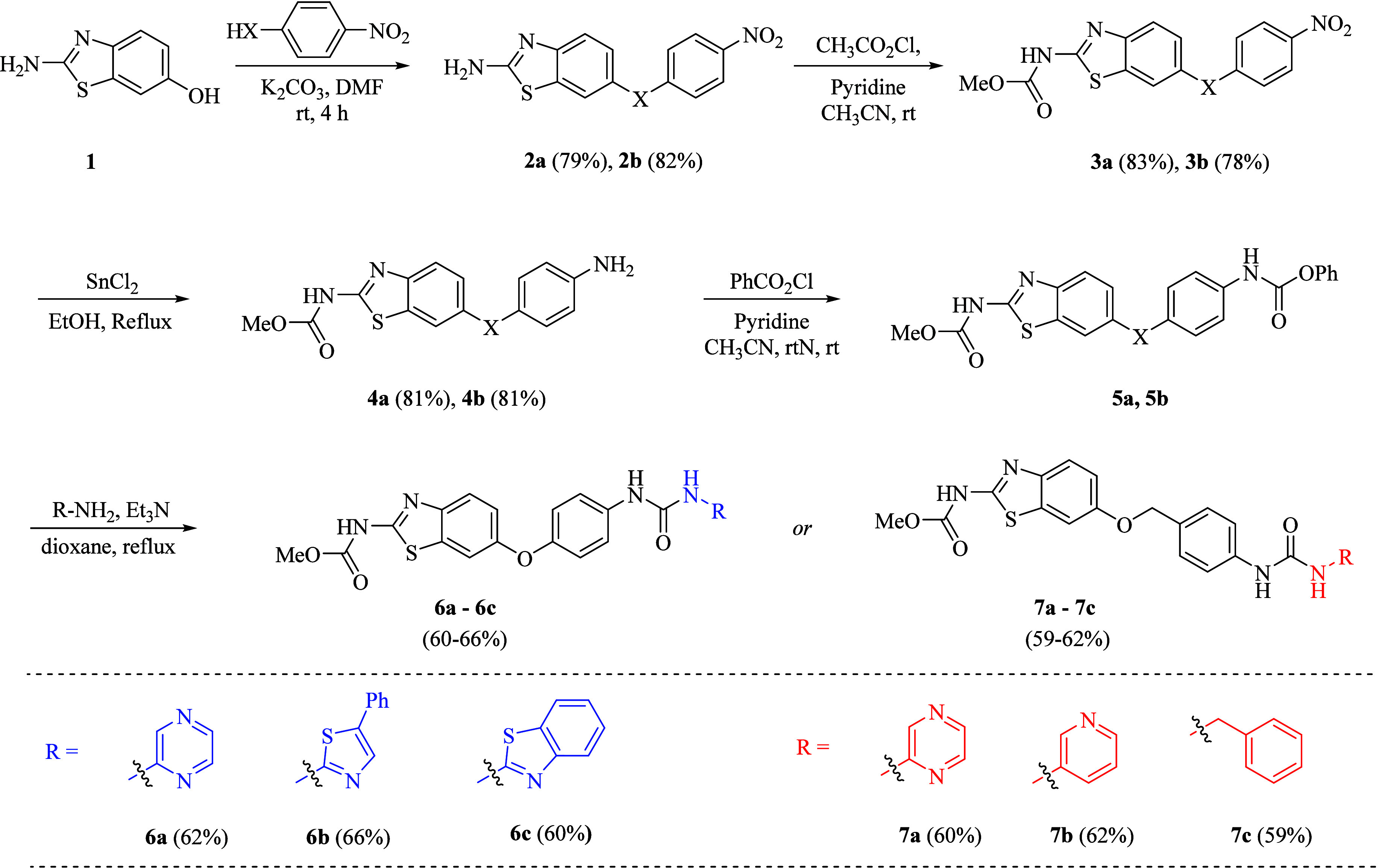
General Synthetic Scheme of Benzothiazole-Based Urea Hybrids **6a**–**c** and **7a**–**c**

**Scheme 2:**
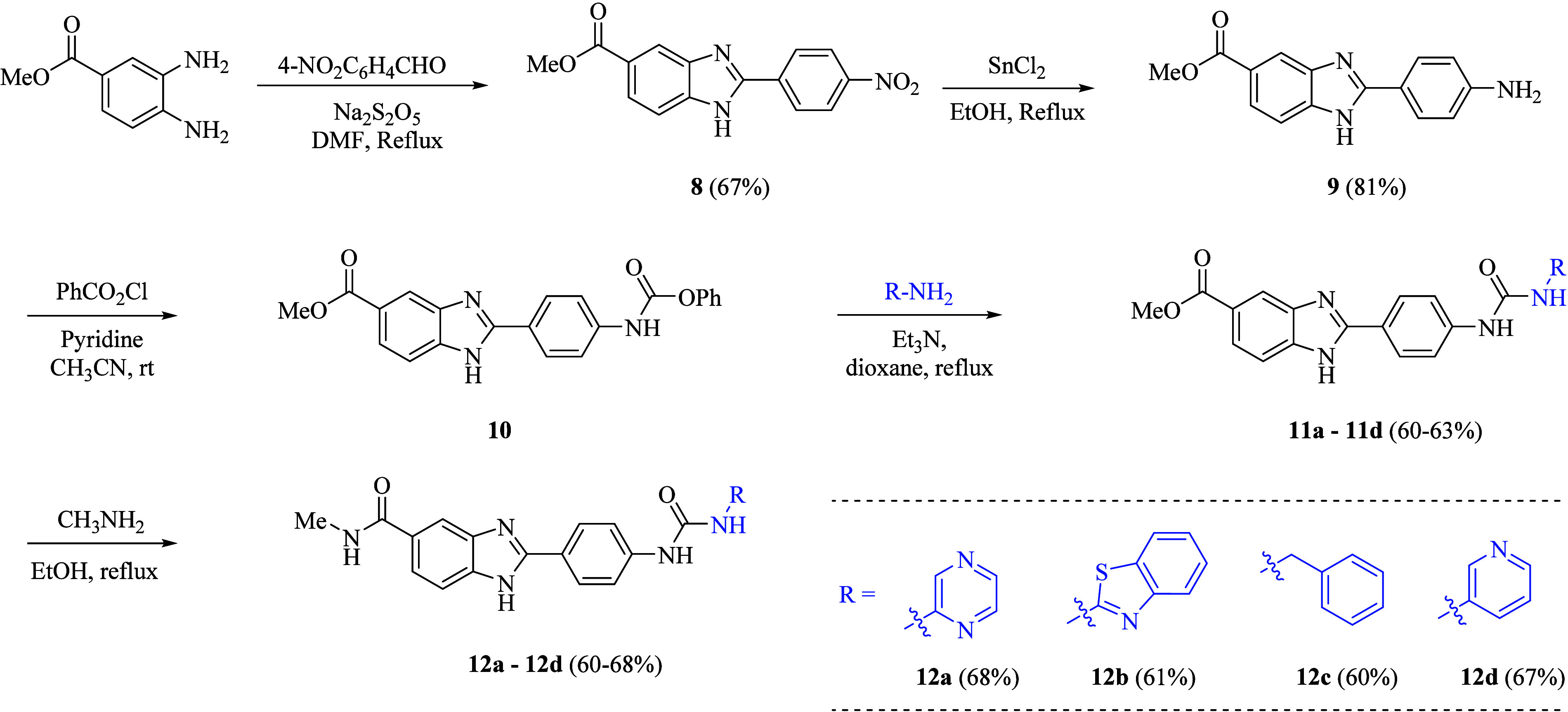
Synthetic Schemes for Target Compounds **11a**–**d** and **12a**–**d**

**Scheme 3:**
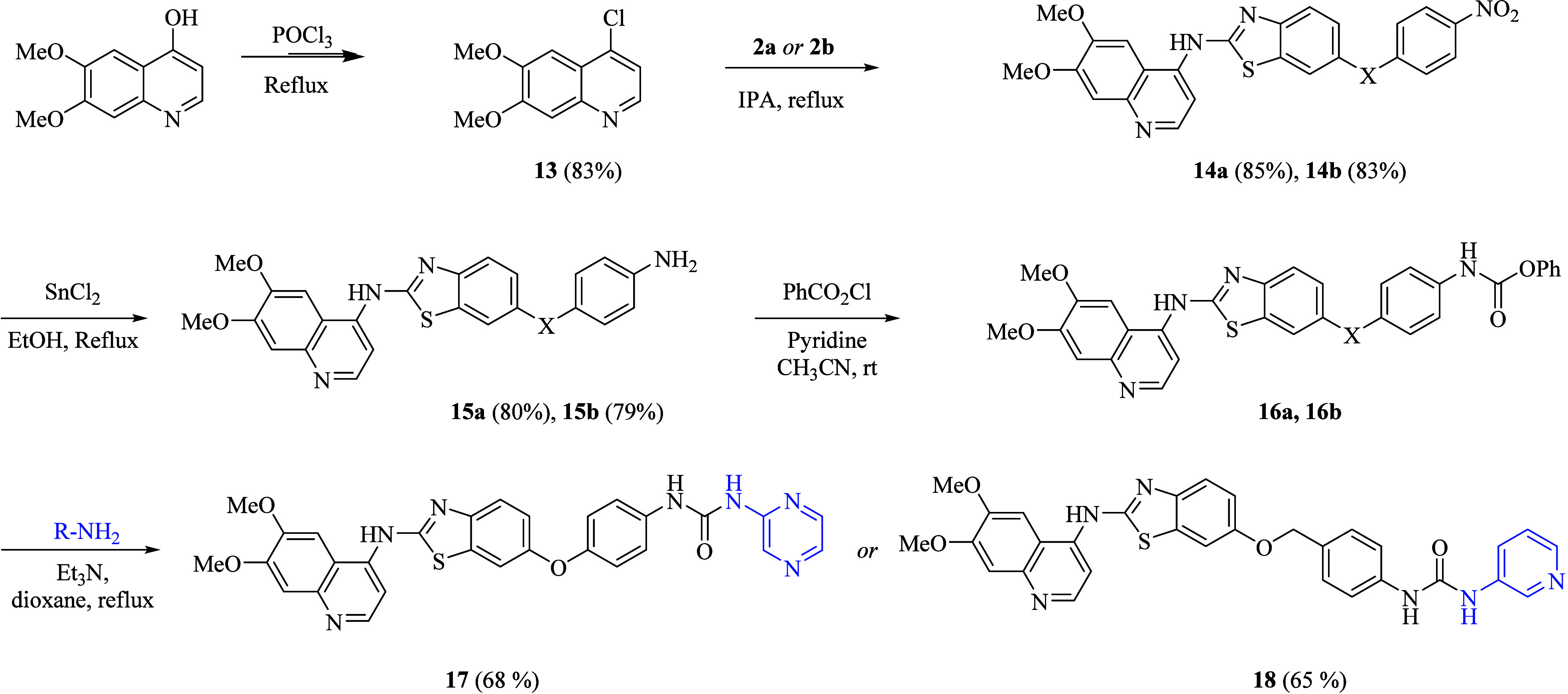
Synthetic Route of Target Compounds **17** and **18**

Initially, for the synthesis of benzothiazole carbamates **4a** and **4b**, commercially available 2-aminobenzothiazol-6-ol **1** was chosen as the starting material. The nucleophilic substitution reaction of 6-hydroxy-2-aminobenzothiazole **1** with 1-chloro-4-nitrobenzene and 1-(bromomethyl)-4-nitrobenzene under standard Williamson ether synthesis resulted in intermediates **2a** and **2b.**

In the next step, condensation of the obtained intermediates **2a** and **2b** with methyl chloroformate by using pyridine as a base in acetonitrile obtained intermediates **3a** and **3b**, which were further subsequently reduced under reflux with SnCl_2_·2H_2_O and concentrated HCl in the presence of ethanol to get key intermediates 1,3-benzothiazole-based carbamates **4a** and **4b**, respectively (Scheme 1). Finally, the desired benzothiazole-based urea hybrids **6a**–**c** and **7a**–**c** were prepared by reacting the previously synthesized benzothiazole carbamates **4a** and **4b** with phenyl chloroformate in acetonitrile using pyridine as a base followed by further reacting of obtained intermediates **5a** and **5b** with different aromatic amines in the presence of trimethylamine (TEA) by using 1,4 dioxane as solvent as shown in Scheme 1. The ^1^1H NMR spectra of the synthesized derivatives **6a**–**c** and **7a**–**c** presented the characteristic three D_2_O exchangeable singlet signals of NH protons, one NH of the carbamate group, and two NH protons of urea. Compounds **6a**,**b** and **7a**–**c** showed the singlet signal at 10.52 ppm assigned to the NH proton of the carbamate, while in **6c**, this signal appeared at 10.86 ppm. The compounds **6a**–**c** demonstrate two singlet signals corresponding to two NH protons of urea at 10.51–9.96 ppm. Simultaneously, the three protons of the OCH_3_ group showed a singlet signal at δ value range from 3.77 to 3.70 ppm. Moreover, the two protons of methylene (−CH_2_) of **6a**–**c** were confirmed by the appearance of a singlet signal at a δ value of 5.40 ppm. The three protons of the benzothiazole scaffold appeared in the range δ 7.54–6.98 ppm, as a singlet or doublet, depending on the attached substituent with it. The ^13^C NMR spectra of compounds **6a**–**c** exhibited the presence of two C=O (carbonyl carbons) with the δ value of 168–163 ppm attributed to carbamate carbonyl, while the carbonyl carbon of urea appeared at a δ range from 158.5 to 156 ppm in addition to one methyl carbon at 52.6–52.2 ppm. In contrast, the singlet signal at δ 68.3–67.5 ppm indicated the presence of methylene carbon in compounds **7a–c**.

Synthesis of our primary starting material, methyl 2-(4-aminophenyl)-1*H*-benzoimidazole-5-carboxylate **8**, was carried out through reacting methyl 3,4-diaminobenzoate with 4-nitrobenzaldehyde in DMF under reflux conditions and in the presence of Na_2_S_2_O_5_ followed by the reduction of the nitro group by using the appropriate amount of SnCl_2_·2H_2_O and concentrated HCl in ethanol. Next, intermediate **9** was stirred with phenyl chloroformate under the same reaction conditions as mentioned above to get the benzimidazole carbamate intermediate **10**. Finally, the carbamate was treated with different amines in 1,4-dioxane in the presence of triethylamine to obtain the methyl benzimidazole carboxylate-based urea analogs as target compounds **11a**–**d** in yields of 60–63%. Reacting to the analogs **11a**–**d** with methylamine in ethanol resulted in methyl benzimidazole carboxamide derivatives **12a**–**d** with yields of 60–68% (Scheme 2). The ^1^H NMR spectra showed the two characteristic D_2_O exchangeable singlet signals of the urea moiety at δ values of 10.24–9.97 ppm located in the synthesized derivatives **11a**–**d** and **12a**–**d**. The NH group of imidazole showed a singlet signal at 12.09 ppm. Moreover, the compounds **11a**–**d** demonstrated the singlet signal at a chemical shift value of 3.83 ppm assigned to the three protons of the methyl group. In contrast, an additional quartet signal appeared at δ 8.70, corresponding to the amidic proton in compounds **12a**–**d**. The ^13^C NMR spectra of compounds **11a**–**d** and **12a**–**d** exhibited the presence of two C=O (carbonyl carbons) with the δ value of 168.5 ppm assigned to the carbonyl group of the ester in compounds **11a**–**d**, while the δ value of 170.2 ppm was attributed to the carbonyl group of the amide moiety of compounds **12a**–**d**. The carbonyl carbon of urea appeared at a δ range from 154.3.5–154.0 ppm of **11a**–**d** and **12a**–**d** simultaneously. In addition, the signals at 52.6–52.2 ppm in compounds **11a**–**d** and at 25 ppm in compounds **12a**–**d** indicated the presence of a methoxy group in **11a**–**d** and a methyl group in compounds **12a-d.**

The preparation of 4-chloro-6,7-dimethoxyquinoline **13** was carried out by treating 6,7-dimethoxyquinoline-4-ol with phosphoryl trichloride (POCl_3_) under reflux conditions. Afterward, compound **13** undergoes nucleophilic substitution with compounds **2a** or **2b** separately in the presence of isopropanol at 82 °C, yielding intermediates **14a** and **14b,** respectively. Next, the nitro group present in these intermediates, **14a** and **14b**, was then subjected to reduction by utilizing tin­(II) chloride (SnCl_2_) and conc. HCl in ethanol under reflux to afford the corresponding amino-substituted derivatives **15a** and **15b.** After that, the mixture of amino phenoxy-substituted compounds **15a** and **15b** and phenyl chloroformate were allowed to stir at room temperature in the presence of acetonitrile and pyridine to afford the corresponding carbamate derivatives **16a** and **16b**, respectively, which were further subjected to react with further various primary amines R-NH_2_ in 1,4-dioxane in the presence of triethylamine (TEA) under reflux conditions, yielding the corresponding urea derivatives **17** and **18** as final compounds (Scheme 3).

The ^1^H NMR spectra of synthesized derivatives **17** and **18** demonstrated three distinctive D_2_O exchangeable singlet signals of NH protons, one NH of the quinoline moiety, and two NH protons of urea. The singlet signal appeared at a chemical shift value of 10.15 ppm, attributed to the one NH of the quinoline moiety, while the singlet signals at 8.86 and 8.71 were assigned to the two NH protons of the urea. In addition, 6 protons of the 6,7-dimethoxy substituent appeared as singlets at 3.88–3.83 ppm. Moreover, the distinctive two protons of methylene (−CH_2_) of compounds **35** were confirmed by the appearance of a singlet signal at a δ value of 5.40 ppm. The ^13^C NMR spectra of compounds **17** and **18** exhibited the presence of one C=O (carbonyl carbons) with a δ value of 156 ppm, attributed to the carbonyl carbon of urea in addition to two methoxy carbons at 55.3–54.0 ppm. In contrast, the singlet signal at δ 68.33–67.50 ppm confirmed the presence of methylene carbon in compound **18.**

### Biological Evaluation

The *in vitro* inhibitory activities of the synthesized benzothiazole **6a–c**, **7a–c**, **17**, and **18** and benzimidazole-urea analogs **11a–d** and **12a–d** were systematically evaluated against VEGFR-2, EGFR, and c-Met kinases, using cabozantinib (a dual VEGFR-2/c-Met inhibitor) and sorafenib (a dual VEGFR-2/EGFR inhibitor) as standard drugs. The IC_5__0_ values are summarized in Table 1.

**Table 1:**
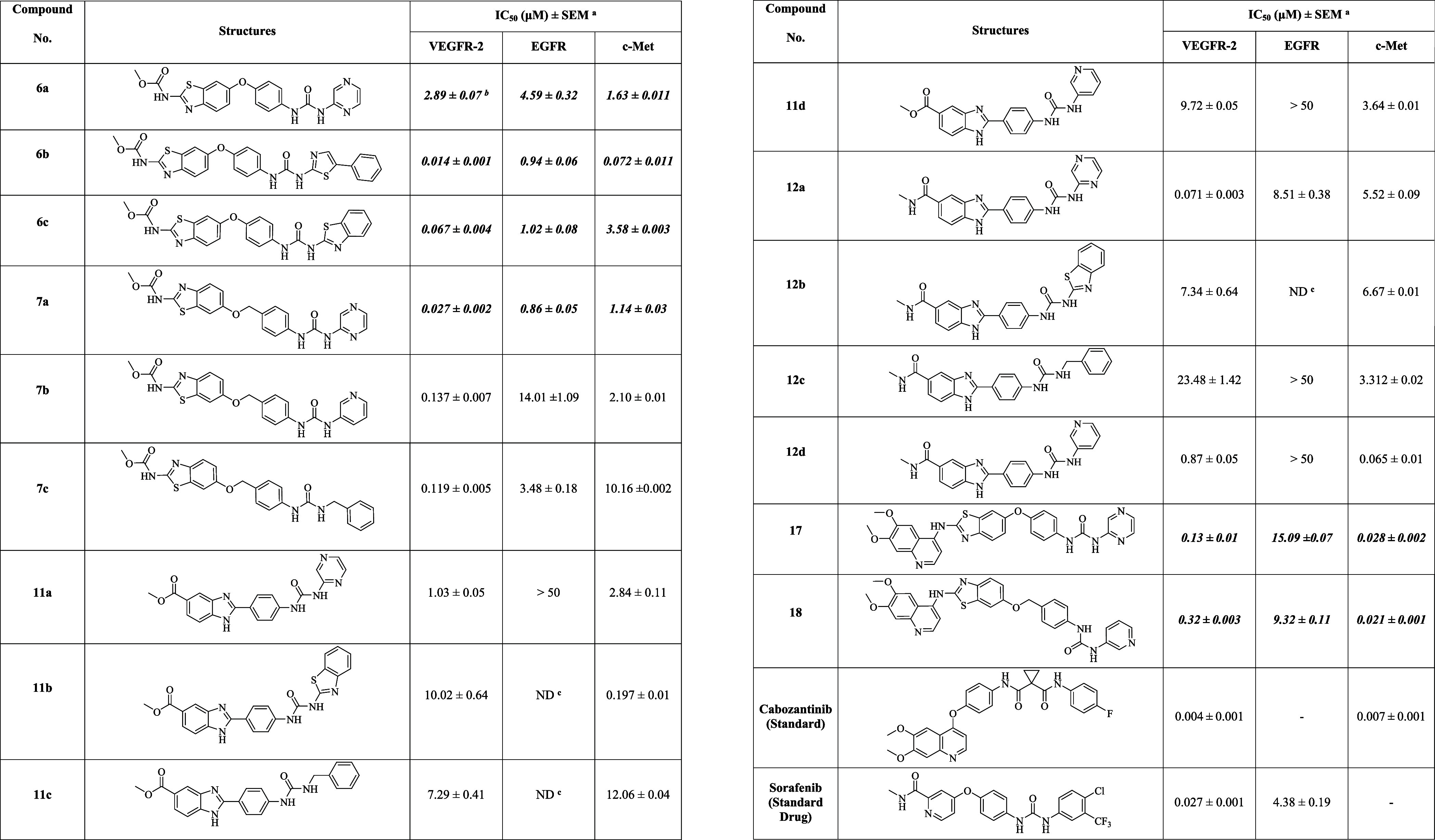
*In Vitro* Analysis of Final Compounds against VEGFR-2, EGFR, and c-Met^d^

a IC_50_ reported as mean ± SEM (*n* = 3).

b Bold and italic boxes indicate the compounds that exhibit balanced (moderate to excellent) biological activities against all tested targets.

c ND = not determined.

d Using GraphPad Prism, nonlinear regression (a four-parameter logistic model) was used to determine IC_50_ values.

#### *In Vitro* VEGFR-2 Inhibitory Assay

For VEGFR-2 inhibition, cabozantinib (IC_5__0_ = 0.004 μM) exhibited the highest potency followed closely by sorafenib (IC_5__0_ = 0.027 μM). The benzothiazole-urea-based series **6a–c**, **7a–c**, **17**, and **18** displayed IC_50_ values within a desirable range, ranging from 0.014 to 2.89 μM, confirming their efficacy from moderate to high levels, while benzimidazole-urea series **11a**–**d** and **12a**–**d** showed minimal inhibitory activity toward VEGFR-2 with an IC_50_ range (0.071–23.48 μM) in comparison to sorafenib (IC_50_ = 0.027 μM). Precisely, out of all 16 screened compounds, four analogues showed remarkable inhibition against VEGFR-2, having IC_5__0_ values of 0.014 μM (**6b**), 0.027 μM (**7a**), 0.067 μM (**6c**), and 0.071 μM (**12a**), confirming their nanomolar efficacy and performance comparable to sorafenib. Next, **7b**, **7c**, **17**, and **18** (IC_5__0_ = 0.137, 0.119, 0.13, and 0.32 μM, respectively) show strong inhibition, though less potent than the standards. In comparison, compounds **6a**, **11a**, and **12d** (IC_5__0_ = 2.89, 1.03, and 0.873 μM, respectively) possessed moderate VEGFR-2 inhibition, while the rest of the compounds, **11b**, **11d**, **11c**, **12c**, and **12b** (IC_5__0_ = 10.02, 9.72, 7.29, 23.48, and 7.34 μM, respectively), exhibited the lowest VEGFR-2 inhibition, respectively (Figure S-48 (Supporting Information file)).

#### *In Vitro* EGFR Inhibitory Assay

The *in vitro* EGFR potential of **6a–c**, **7a–c**, **11a**, **12a–d**, **17**, and **18** was further evaluated by applying a standard procedure, taking sorafenib (IC_50_ = 4.38 ± 0.19 μM) as a positive control, and their IC_50_ values are presented in Table 1. Considering the results, out of the screened compounds, **6b** (0.94 ± 0.06 μM), **6c** (1.02 ± 0.08 μM), **7a** (0.86 ± 0.05 μM), and **7c** (3.48 ± 0.18 μM) exhibited the strongest EGFR inhibition potential, compared to sorafenib, as depicted in Figure S-48 (Supporting Information file). Next, compounds **6a** (4.59 ± 0.32) **12a** (8.51 ± 0.38 μM), and **18** (9.3 ± 0.01 μM) displayed moderate inhibition potential while **7b** (14.01 ± 1.09) and **18** (15.09 ± 0.04 μM) exhibited the weakest inhibition, and all the other remaining compounds, **11a** and **12b–d**, showed greater than 50% EGFR inhibitory potential.

#### *In Vitro* c-Met Inhibitory Assay

For c-Met inhibition, cabozantinib (IC_5__0_ = 0.007 μM) displayed potent activity, whereas sorafenib was inactive (IC_5__0_ > 50 μM). Out of the screened analogues, **18** (0.021 μM), **17** (0.028 μM), **12a** (0.052 μM), **12d** (0.065 μM), **6b** (0.072 μM), and **11a** (0.084 μM) exhibited strong inhibition comparable to that of cabozantinib. Compound **7a** (0.176 μM) also retained significant potency, while **6a** (1.63 μM), **7b** (2.10 μM), and **11b** (0.197 μM) were moderately active. Compounds **7c** (10.16 μM) and **11c** (12.06 μM) showed the weakest inhibition. Overall, these findings demonstrate that several benzothiazole/benzimidazole-based urea analogues **6b**, **12a**, **12d**, **17**, and **18** exhibited multitarget kinase inhibition, precisely showing dual potency against VEGFR-2 and c-Met, comparable to cabozantinib, while outperforming sorafenib as depicted in Figure S-48 (Supporting Information file).

### Structure–Activity Relationship

The SAR analysis of the synthesized benzothiazole/benzimidazole-based urea derivatives revealed distinct structural determinants governing their multikinase inhibitory activity against VEGFR-2, EGFR, and c-Met compared to sorafenib and cabozantinib (standard drugs).

In the current study, we employed two main scaffolds, 2-amino-6-hydroxybenzothiazole and methyl 2-(4-nitrophenyl)-1*H*-benzo­[*d*]­imidazole-5-carboxylate. On the right side, the 2-amino-6-hydroxy-benzothiazole core connected with the urea moiety via phenoxy and benzyloxy linkers, while its 2-amino group on the left-hand side was derivatized into carbamate and quinoline analogs. In contrast, reduction of the nitro group in the benzimidazole scaffold afforded the corresponding amine for urea formation and subsequent modification of the ester functionality yielded both ester and amide derivatives.

Series 1 (**6a–c**) comprises compounds bearing a carbamate functionality on the left-hand side, while a phenoxy group is linked to the urea moiety on the right-hand side. Structural diversification within this series was achieved by varying the substituents attached to one of the urea nitrogens. Notably, the urea derivative synthesized from 2-amino phenylthiazole emerged as a potent multikinase inhibitor, exhibiting IC_5__0_ values of 0.027 μM for VEGFR-2, 0.94 μM for EGFR, and 0.072 μM for c-Met.

In series 2 (**7a–c**), the urea moiety linked via a benzyloxy bridge to the benzothiazole scaffold yielded potent multitarget inhibitors. The pyrazine analogue **7a** exhibited strong VEGFR-2, EGFR, and c-Met inhibition, while *N*-pyridine (**7b**) and *N*-benzyl (**7c**) substituents exhibited comparatively reduced potency.

A clear difference was observed between ester-linked (**11a**–**d**) and amide-linked (**12a**–**d**) benzimidazole series. Ester derivatives were generally weak, with pyrazine-based compound **11a** showing only moderate VEGFR-2 inhibition with an IC_50_ = 1.03 μM but weak c-Met activity (IC_50_ = 2.84 μM). While *N*-benzothiazole (**11b**), *N*-benzyl (**11c**), and 3-aminopyridine (**11d**) groups resulted in decreased potency against VEGFR-2 and inconsistent c-Met activity. By contrast, the amide-linked analogs (**12a–d**) exhibited improved potency. The pyrazine-based compound **12a** showed strong VEGFR-2 inhibition (IC_5__0_ = 0.071 μM) while maintaining moderate activity against EGFR (IC_5__0_ = 8.51 μM) and c-Met (IC_5__0_ = 5.52 μM), suggesting that the amide linkage may contribute to enhanced stabilization through hydrogen-bonding interactions.

To mimic the structural features of cabozantinib, quinoline moieties were introduced to enhance interactions within the hydrophobic back pocket and hinge region of the kinases. The quinoline-containing derivatives demonstrated exceptional activity, underscoring their key role in effective binding. Among them, pyrazine-based compound **17** exhibited remarkable c-Met inhibition (IC_5__0_ = 0.028 μM) along with potent VEGFR-2 inhibition (IC_5__0_ = 0.13 μM), establishing it as a selective dual VEGFR-2/c-Met inhibitor. Likewise, the pyridine-based analogue **18** achieved outstanding c-Met inhibition (IC_5__0_ = 0.021 μM) and moderate inhibition of VEGFR-2 (IC_5__0_ = 0.32 μM) and EGFR (IC_5__0_ = 9.30 μM). The balanced inhibition profile and exceptional c-Met potency of compound **18** can be attributed to the presence of a flexible linker, which likely facilitates optimal orientation within the kinase active site.

Collectively, the SAR analysis highlights that urea/amide linkers, planar electron-rich heteroaryl rings (thiazole, pyrazine, quinoline), and rigid aromatic linkers are key determinants of multikinase potency. Notably, compound **6b** emerges as the most promising inhibitor, validating this integrated design strategy for future multitargeted kinase inhibitors (Figure 3).

**Figure 3:**
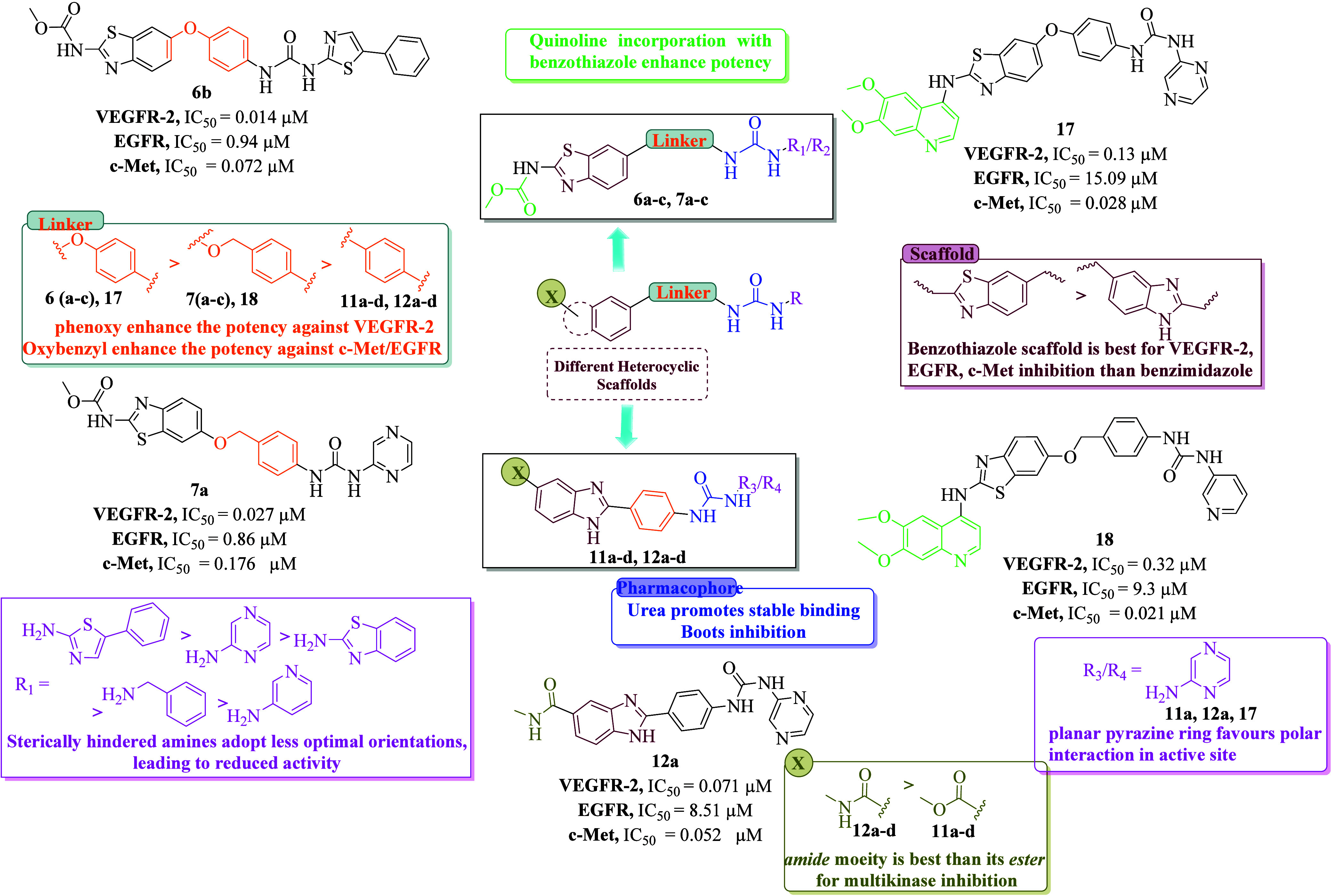
Structure–activity relationship of newly synthesized analogues as multikinase inhibitors of VEGFR-2, EGFR, and c-Met.

#### *In Vitro* Antiproliferative Assay against MCF7 and A549 Cells

Next, we applied the 3-day sulforhodamine B (SRB) proliferation assay to evaluate the anticancer potential of benzothiazole/benzimidazole-based urea derivatives against the lung cancer cell line (A549) and the human breast cancer cell lines (MCF7), and the results were expressed as IC_50_ (Table 2). In MCF-7 cells, only four compounds were active with an IC_50_ below 10 μM. Specifically, **17**, **11b**, and **18** showed the lowest IC_50_ values of 0.84 ± 0.05, 1.34 ± 0.65, and 1.98 ± 0.11 μM, respectively, making them most potent against MCF-7 cells, followed by **12a**, showing an IC_50_ value of 7.61 ± 3.60 μM. Similarly, in A549 cells, compounds **17**, **18**, and **12d** also show activity with IC_50_ values of 0.69 ± 0.03, 0.74 ± 0.01, and 0.96 ± 0.08 μM, making them potent in A549, like MCF7, followed by **12a**, having an IC_50_ value of 12.5 ± 4.27 μM.

**Table 2:** IC_50_ (μM) ± SEM Values of the Most Potent Derivatives in Cancerous MCF7 A549 Cells and Normal Human Embryonic HEK-293 Cells^a^

	IC_50_ (μM) ± SEM			
compounds	MCF (μM)	A549 (μM)	HEK-293 (μM)	*N*
**6a**	>100	>100	ND	3
**6b**	>100	>100	>100	3
**6c**	>100	>100	90.31 ± 3.01	3
**7a**	89.84 ± 34.17	>100	>100	3
**7c**	>100	79.7 ± 14.22	41.27 ± 2.49	3
**11b**	1.34 ± 0.65	0.96 ± 0.08	3.24 ± 0.17	3
**11c**	>100	>100	10.79 ± 1.63	3
**12a**	7.61 ± 3.60	12.5 ± 4.27	35.77 ± 2.41	3
**17**	0.84 ± 0.05	0.69 ± 0.03	>100	3
**18**	1.98 ± 0.11	0.74 ± 0.01	>100	3

a SEM = standard error mean ± *n* = 3.

Notably, although potent compounds **6b**, **6c**, and **7a** against VEGFR-2/EGFR as well as c-Met did not exhibit cytotoxic activity in MCF-7 and A549 cancer cell lines, they displayed excellent antiangiogenic efficacy in the CAM assay, which indicated strong *in vivo* effects. This discrepancy likely reflects the marginal role that VEGFR-2 plays in the survival of these epithelial cancer cell lines, as VEGFR-2 primarily mediates angiogenesis in endothelial cells rather than promoting tumor cell proliferation. The *in vivo* activity indeed supports the notion that the compounds act through the inhibition of angiogenesis and corroborates that VEGFR-2 is an established key regulator of tumor vascularization.

#### *In Vitro* Cytotoxicity Evaluation on Normal Human Embryonic HEK-293 Cells by the MTT Assay

The *in vitro* cytotoxic evaluation of the synthesized compounds against normal human kidney cell line (HEK293) cells by using an MTT (3-{4,5-dimethylthiazol-2-yl}-2,5-diphenyl-tetrazolium-bromide) assay. The results of the assay are listed in Table 2. Compounds **6b**, **7a**, **17**, and **18** demonstrated inactivity against normal HEK-293 cells with IC_50_ values exceeding 100 μM (>100 μM) and indicated negligible cytotoxicity. The remaining tested compounds exhibited measurable cytotoxicity, with IC_50_ values of 3.24, 10.79, 35.77, 41.27, and 90.31 μM (Table 2). Among these compounds, **11b** and **11c** with IC_50_ values of 3.24 and 10.79 μM, respectively, showed the highest cytotoxicity while compounds **7c** and **12a** showed considerable cytotoxicity.

##### Acute Toxicity Test for Compounds **6b** and **18**

Since anticancer potential must be accompanied by an acceptable safety profile, we performed acute toxicity studies and the LD_50_ values of the most potent compounds were determined. This assessment provides insights into the systemic tolerability and preliminary therapeutic index, supporting their potential as lead candidates for further preclinical development.

The acute toxicity test of compounds **6b** and **18** was evaluated in albino mice employing the up-and-down method (OECD Guideline 425). Individually, the doses are given to the mice; if the previous dose (mice) survives, a larger dose is administered to the next one. Conversely, a smaller dose is given if the previous mice die. For each succeeding animal, the dosage is adjusted either up or down based on the outcomes of the previous mice. The purpose of this study was to estimate the oral toxicity of compounds **6b** and **18** administered via oral gavage to mice at various doses and different numbers of mice for each concentration. During the initial phase, all mice died after receiving a dose of compound **6b** and **18** at 1250 mg/kg BW and 1500 mg/kg BW. Consequently, several doses of compounds were administered to the mice according to the UDP as per Table 3a,b. At the dosage of 500 mg/kg BW (**6b**), one out of nine mice died, which indicated the dosage to be lowered; at the dosage of 250 mg/kg BW, all of the animals survived. Similarly, at dosages of 300, 100, and 50 mg/kg of BW (compound **18**), no mice died. All the animals that survived the 72 h observation period showed no toxicity symptoms.

**Table 3:** Mice Mortality Brought on by Compounds **15b** and **35**, Administered by Oral Gavage, along with the Survival Durations Associated with Each Treatment

doses (mg/kg)	no. of animals tested	death no.	survival time	% mortality
**(a) Compound 6b**				
1250	3	3	28 min; 32 min; 44 min	100%
1000	5	3	35 min; 1 h; 2.5 h	60%
750	7	3	2 h; 7.5 h; 9 h	42.86%
500	9	1	20 h	11.11%
250	10	0	>72 h	0%
100	10	0	>72 h	0%
50	10	0	>72 h	0%
**(b) Compound 18**				
1500	3	3	18 min; 23 min; 41 min	100%
1200	5	4	35 min; 90 min; 2h; 2.5h	80%
900	7	3	2h; 4.5h; 7h	42.86%
600	9	3	9h; 12h; 15h	33.33%
300	10	0	>72h	0%
100	10	0	>72h	0%
50	10	0	>72h	0%

To assess the No Observed Adverse Effect Level (NOAEL), mice were also given doses of compound **6b** at 100 and 50 mg/kg BW and compound **18** at 300, 100, and 50 mg/kg BW. All those mice survived the 72 h observations (Table 3) and showed no signs of toxicity. Both compounds caused dose-dependent mortality in this method. As illustrated in Figure 4, a nonlinear regression fitting approach was used to predict the oral LD_50_ of compound **6b** to be 821 mg/kg BW (95% CI: 688.2 to 953.2 mg/kg BW; *R* ^2^ = 0.9623) and the oral LD_100_ to be 1250 mg/kg BW. While the estimated LD_50_ for compound **18** was 841.3 mg/kg (95% CI: 637.3 to 1042 mg/kg BW; *R* ^2^ = 0.9506), the oral LD_100_ was 1500 mg/kg BW (GraphPad Prism 8).

**Figure 4:**
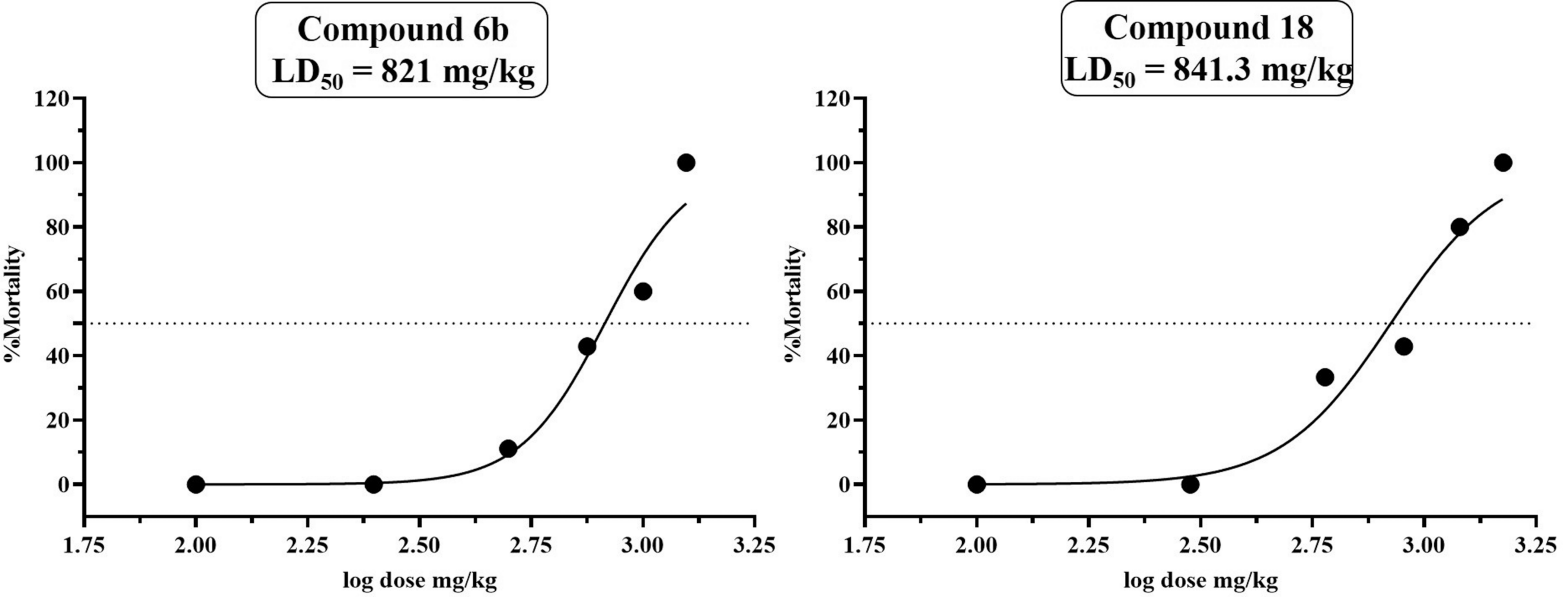
Dose–response mortality curve of oral compound **6b** (95% CI: 688.2 to 953.2 mg/kg BW; *R* ^2^ = 0.9623) and **18** (95% CI: 637.3 to 1042 mg/kg BW; *R* ^2^ = 0.9506) in mice presents the log of its concentration on the *x*-axis against the percentage of mortality on the *y*-axis, indicating the LD_50_ value. Nonlinear regression with a 95% confidence interval was used to compute the LD_50_ values (GraphPad Prism 8).

#### *In Vivo* Antiangiogenic Activity through the Chick Chorioallantois Membrane (CAM) Assay

In the current study, the CAM assay was applied to assess the anti-angiogenic efficiency of the active compounds, as this assay ensures an economical and adaptable *in vivo* model, commonly employed in oncology research to assess neovascularization. Therefore, the *in vivo* CAM model was selected as a reliable approach to assess the anti-angiogenic action of chosen compounds for the blockage of vascularization. In the current experiment, after a 24 h incubation period, a significant reduction in vessel density was observed, indicating the anti-angiogenic potential of the tested substances (Figure 5). Moreover, it has been analyzed from the anti-angiogenic results of the tested compounds, observed after the sample inoculation and post-treatment after 24 h, that **6b** demonstrated the most pronounced anti-angiogenic action while compounds **7a**, **17**, and **18** exhibited considerable anti-angiogenic activity, but slightly weaker. These observations highlight the value of the CAM assay as a valid and effective preclinical model for the screening of anti-angiogenic compounds. The inhibition of vessel formation by the compounds tested in this study indicates their promise as drug candidates for the inhibition of tumor-induced angiogenesis.

**Figure 5:**
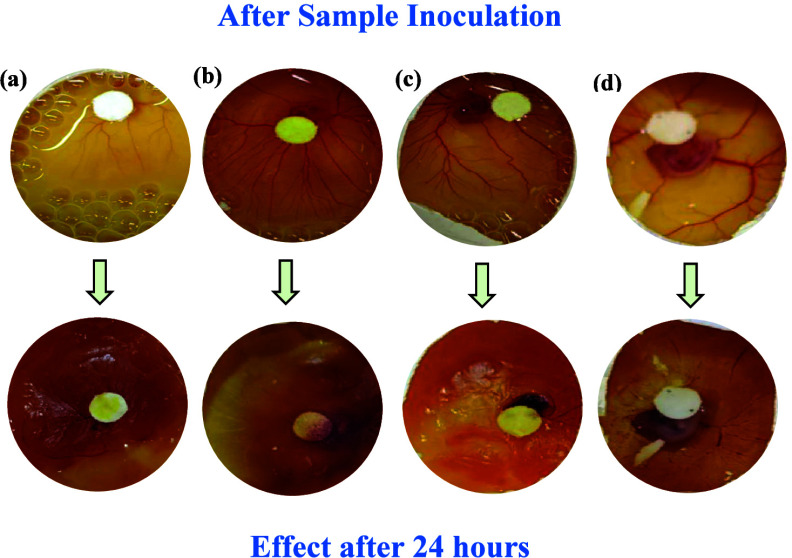
Visual representation of antiangiogenic effects of compounds **6b**, **7a**, **17**, and **18** using the CAM model.

#### *In Silico* Studies

##### Molecular Docking of Compounds **6b**, **7a**, **11a**, **12a**, and **17** against VEGFR-2

To explore the binding pattern of the newly synthesized derivatives with VEGFR-2 (PDB ID: 4ASD), a molecular docking study was conducted by applying the Molecular Operating Environment (MOE 2016 08.02). At first, the docking protocol was validated by redocking the cocrystallized ligand (sorafenib) inside the active pocket of the VEGFR-2. The resulting docked pose closely overlapped with the original pose, showing a low RMSD of 0.24 Å. Sorafenib demonstrated a binding energy of −9.3187 kcal/mol and effectively occupied the four key regions within the active site of the VEGFR-2. In the hinge region of VEGFR-2 receptor, the *N*-methyl picolinamide moiety formed a hydrogen bond with Cys919 and established six hydrophobic interactions (HIs) with Leu1035, Phe918, Leu840, and Ala866. The central phenyl ring (linker) is engaged in five HIs with Val868, Val916, Cys1045, Lys868, and Ala866. The pharmacophore moiety, i.e., urea group, formed three hydrogen bonds with Glu885 and Asp1046. The 1-chloro-2-(trifluoromethyl) benzene moiety contributed five HIs with Leu1044, Cys1045, Val898, and Leu889. Additionally, this moiety formed one hydrogen bond with Val 899 (Figure 6).

**Figure 6:**
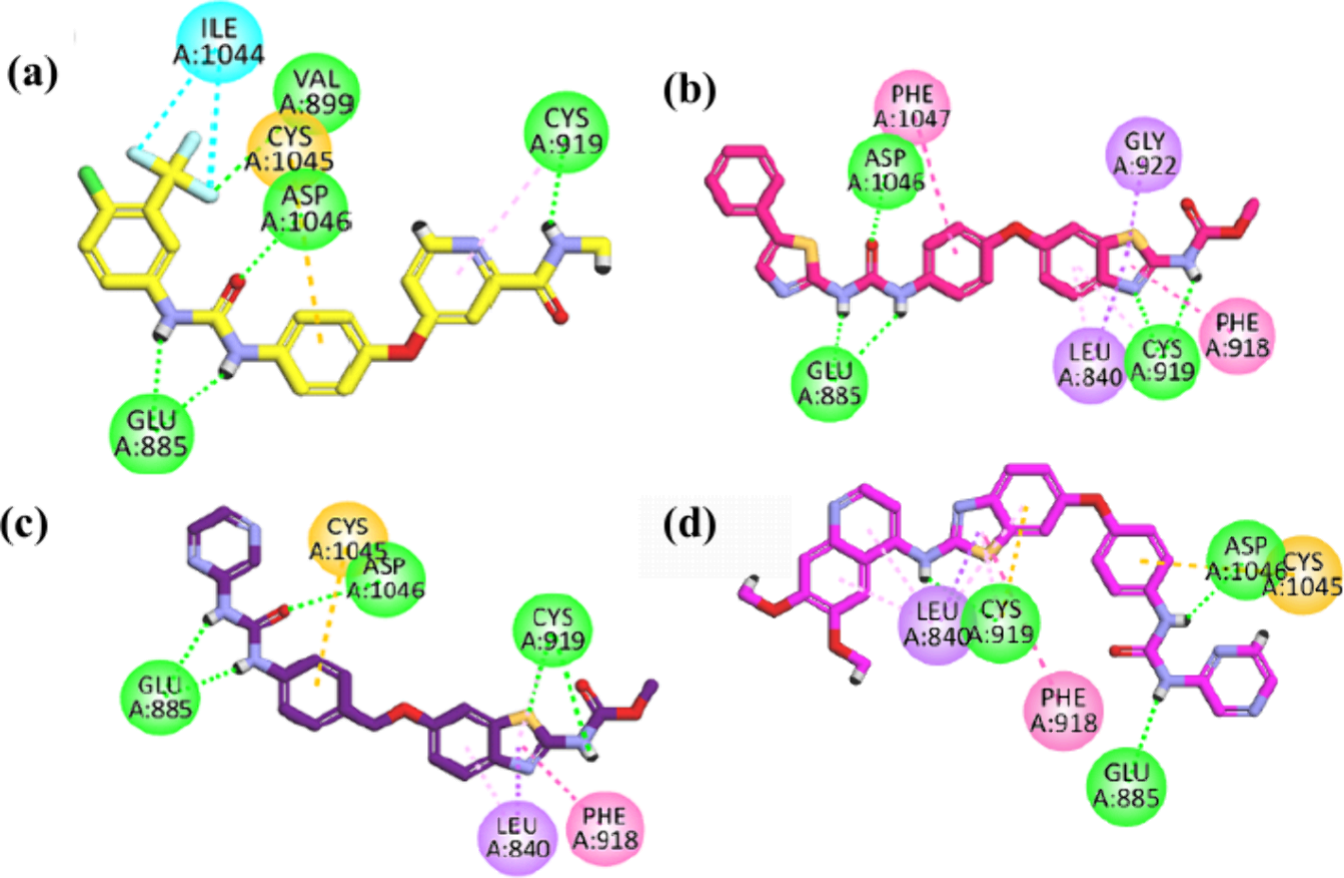
2D interaction plot of (a) sorafenib and compounds (b) **6b**, (c) **7a**, and (d) **18** in the VEGFR-2 active site.

Compound **6b** showed a binding energy of −9.6045 kcal/mol. First, the benzothiazole carbamate moiety, specifically occupying the hinge region of the receptor, formed two hydrogen bonds with Cys919 and hydrophobically interacted with Leu840, Leu1035, Ala866, Cys919, Val848, Gly922, Leu840 (π-sigma), and Phe918 (π–π stacked). Next, the phenoxy linker between the ATP region and the DFG-motif of the receptor engages in five hydrophobic interactions with Cys1045, Val848, Val916, Val899, Lys868, and Phe1047 (π-sigma). The urea pharmacophore accommodated the DFG-motif of the receptor, forming three hydrogen bond interactions with two amino acids, i.e., Glu885 and Asp1046. Finally, the terminal phenyl thiazole moiety occupied the allosteric pocket, hydrophobically interacting with Leu889, Ile892, Cys1024, Leu1019, and Ile888 (Figure 6).

Compound **7a** displayed a binding energy of −9.2129 kcal/mol. In the hinge region of the enzyme, the methyl benzothiazole carbamate moiety engaged in two hydrogen bonds with Cys919, as well as five hydrophobic interactions with Val848, Leu840, Leu1035, Ala866, and Cys919. This moiety interacted with residues Leu840 (π-sigma) and Phe918 (π–π stacked). The phenoxy (linker) moiety engaged in four hydrophobic interactions with Val916, Cys1045, Val899, and Lys868. The urea moiety formed three conventional hydrogen bonds with Glu885 and Asp1046 through nitrogen and oxygen atoms, as depicted in Figure 6.

Compound **17** displayed a binding energy level of −10.7958 kcal/mol. In the hinge region of the enzyme, the benzothiazole moiety engaged in a hydrogen bond with Cys919 through its NH and formed hydrophobic interactions with Val848, Leu1035, and Ala866. This moiety also interacted with Leu840 (π-sigma) and Phe918 (π–π stacked). The quinoline moiety hydrophobically interacted with residues Leu840, Phe1047, and Leu1035. The phenoxy (linker) interacted hydrophobically with Val848, Val916, Val899, Lys868, and Cys1045 (π-sulfur). The urea moiety formed two conventional hydrogen bonds with Glu885 and Asp1046 through its NH groups. The terminal pyrazine ring interacted with Ile892 and Leu889 hydrophobically, as depicted in Figure 6.

Compound **11a** showed a binding energy of −8.3978 kcal/mol. First, a benzimidazole ester moiety accommodated the hinge region of the receptor, forming eight hydrophobic interactions with Leu840, Leu1035, Phe918, Val848, and Ala866. The phenyl group (linker) hydrophobically interacted with Val848, Val916, Ala866, Cys1045, and Val899. Furthermore, the pharmacophore moiety (urea) formed three hydrogen bonds with key residues Asp1046 and Glu885. Lastly, the pyrazine moiety, which occupied the allosteric pocket of the enzyme, was involved in only one hydrophobic interaction with Leu889 (Figure 7.

**Figure 7:**
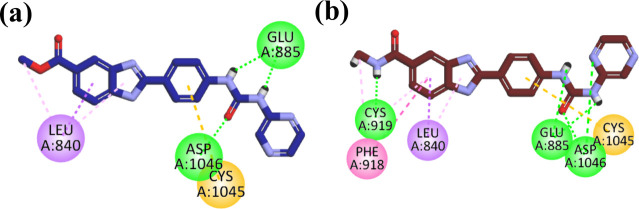
2D interaction diagram of compounds (a) **11a** and (b) **12a** in the VEGFR-2 active site.

Compound **12a** exhibited a binding energy score of −8.2721 kJ/mol. First, the benzimidazole amide moiety accommodated the hinge region of the receptor, formed a hydrogen bond with Cys919, and showed hydrophobic interactions with Leu840, Ala866, Cys919, Phe918, Leu1035, and Val848. The phenyl linker engages in four hydrophobic interactions with Val916, Val899, Cys1045, and Val848. Furthermore, the urea pharmacophore forms hydrogen bonds with Asp1046 and Glu885. Lastly, the pyrazine moiety occupied the allosteric pocket and interacted through only one hydrogen bond with Asp1046 and one hydrophobic interaction with Leu889. These interactions with the VEGFR-2 receptor showed that the amide group interacted much more strongly with the key residue compared to its corresponding ester moiety (Figure 7).

##### Docking Studies of Compounds **6b**, **7a**, **12a**, and **18** against EGFR

To explore the binding interactions pattern of the most potent synthesized compounds **6b**, **7a**, **12a**, and **18** toward EGFR, docking studies were performed. The 3D crystallographic structure of EGFR (PDB ID: 1M17) was used, and erlotinib was chosen as the standard reference. First, for validation of the protocol, redocking of the crystallized ligand (erlotinib) was carried out, exhibiting an RMSD of 1.48 Å. The key active site residues present inside the ATP pocket of the EGFR were Met 793, Cys797, Thr854, Asp831, Thr766, Gly96, Leu694, Phe699, Val702, Lys721, Thr830, and Asp831. The binding interaction pattern plot is displayed in Figure 8.

**Figure 8:**
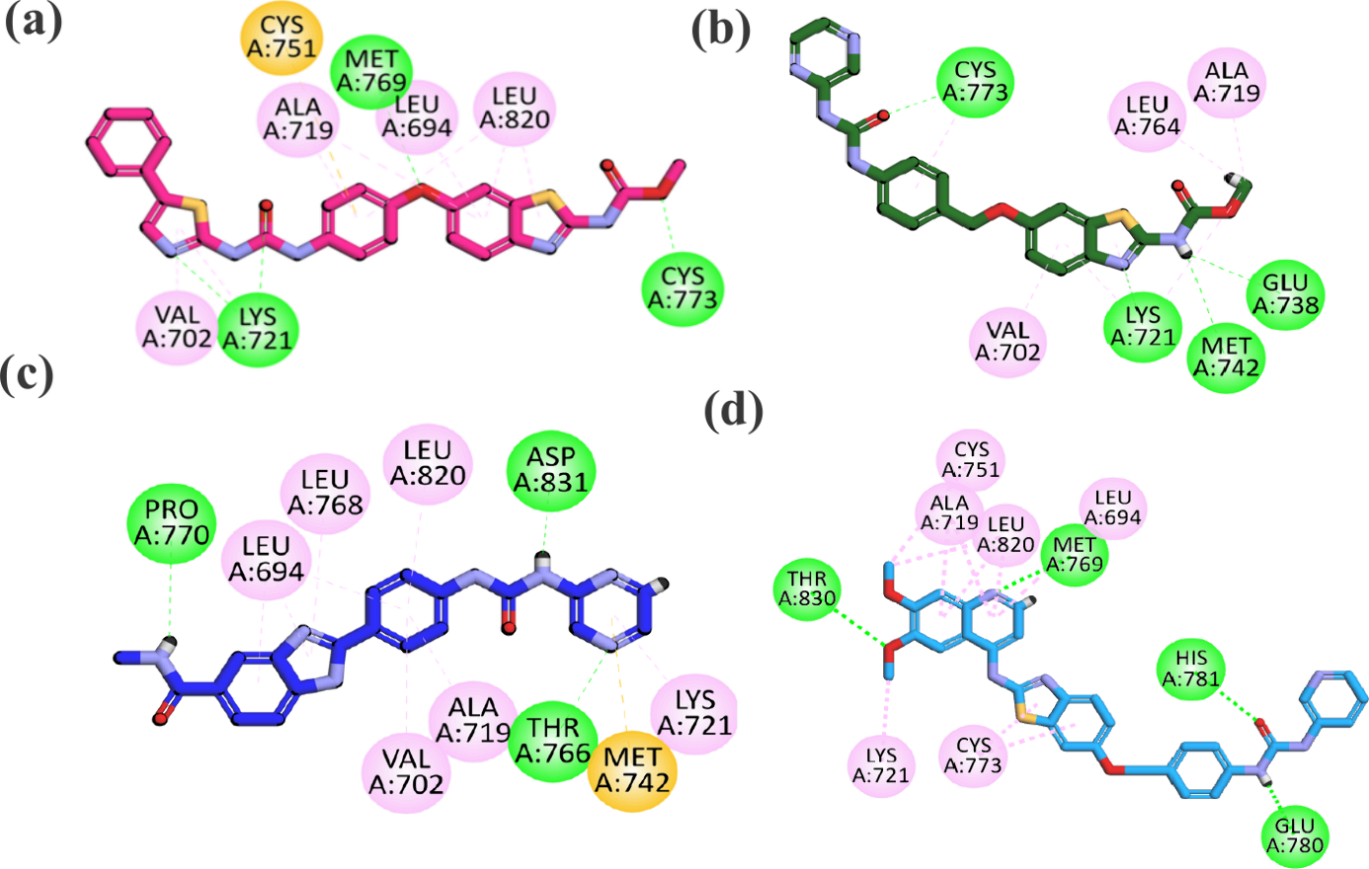
2D interaction plots of (a) **6b**, (b) **7a**, (c) **12a**, and (d) **18** in the EGFR active site.

Compound **6b** exhibited a binding energy score of −7.0363 kcal/mol, and the binding pattern analysis showed that the phenyl thiazole moiety formed a hydrogen bond interaction with Lys721 and two π-alkyl interactions with Val702. The Met769 residue again serves as a key H-bond donor/acceptor, anchoring the ligand at the hinge region. Additional hydrophobic interactions were formed with Ala719, Leu694, and Leu820. Notably, Cys773 was involved in hydrogen bonding at the periphery of the pocket, enhancing binding stability. Lys721 and Cys751 also contributed to polar contacts, consistent with typical binding interactions seen with other known EGFR inhibitors, Figure 8.

Compound **7a** exhibited a binding energy score of −7.0722 kcal/mol. The binding orientation of **7a** displayed that the heteroaromatic ring moiety interacted with the key amino acids Glu738, Met742, and Lys721 via hydrogen bonding. Additionally, this moiety also hydrophobically interacts with Leu764, Val702, and Ala719. The urea moiety occupied the gatekeeper region and interacted with Cys773 through hydrogen bonding. Furthermore, hydrophobic moiety II interacted hydrophobically with Cys773. Thus, all these engagements align well with the known pharmacophore features necessary for effective EGFR inhibition (Figure 8).

Compound **12a** exhibited a binding energy of −7.2193 kcal/mol. The binding orientation of **12a** revealed that the amide functionality engaged in a hydrogen bond with the Pro770 residue. The benzimidazole moiety engaged in hydrophobic interactions with Leu694 and Leu768, respectively. The phenyl linker interacted hydrophobically with Leu768, Leu820, Va702, and Ala719. The urea moiety formed a hydrogen bond with Asp831 through its NH group, and the last pyrazine moiety interacted with Thr766 (H-bond), Met742­(π-sulfur), and Lys721 (π-alkyl), respectively (Figure 8).

Compound **18** exhibited a binding energy score of −7.9697 kcal/mol. The binding orientation of **18** displayed that the quinoline moiety interacted with the key amino acids Met769 and Thr830 through hydrogen bonding. Additionally, this moiety also hydrophobically interacted with Lys721, Ala719, Cys751, Leu820, and Leu694, respectively. The benzothiazole moiety hydrophobically interacted with only Cys773. Lastly, the urea moiety occupied the gatekeeper region and interacted with His781 and Glu780 through hydrogen bonding, as illustrated in Figure 8.

##### Docking Studies of Compounds **6c**, **7a**, **12a**, and **18** against C-Met

To investigate the binding interaction analysis of synthesized compounds **6c**, **7a**, **12a**, and **18** inside the active site pocket of C-Met, docking studies were carried out. For performing the docking procedure, the three-dimensional crystallographic structure of C-Met kinase (PDB ID: 3LQ8) was downloaded from the RCSB protein data bank and protonated, minimized, and prepared by using the previously reported protocol. Moreover, Foretinib was chosen as the standard reference. Furthermore, to validate the docking protocol, redocking of the crystallized ligand (Foretinib) was carried out, bearing the RMSD = 0.0128 Å.

The active site of c-MET kinase is surrogated with Asp1222, Lys1110, Met1160, Phe1223, TYR1159, Ile1084, Met1211, Met1131, Ala1108, Pro1158, Leu1157, Ala1221, Phe1134, His1202, and Val1092. Therefore, depending on the molecular docking predictions, we screened out the compounds that achieved stable interactions with the active amino acid residues located inside the pocket of the c-Met enzyme. The binding interaction pattern of potent compounds **6c**, **7a**, **12a**, and **18** is displayed in Figure 9.

**Figure 9:**
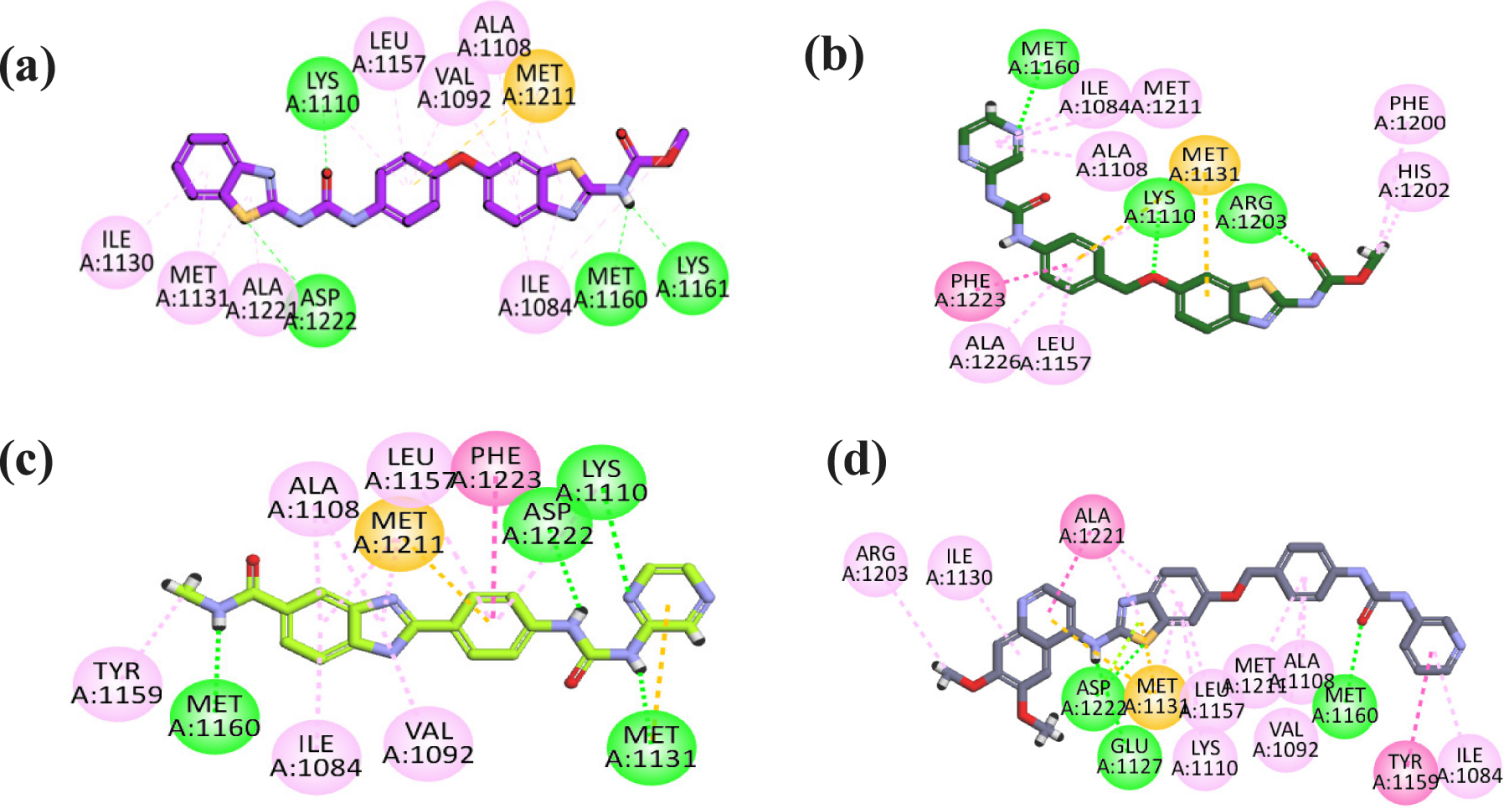
2D interaction diagram of compounds (a) **6c**, (b) **7a**, (c) **11a**, and (d) **18** in the C-Met active site.

Compound **6c** showed a docking energy of −9.36 kcal/mol. The carbamate substituent attached to the benzothiazole moiety of **6c** interacted with highly conserved residues Met1160 and Lys1161 through hydrogen bonding surrounding the hinge region of the receptor. Additionally, this moiety hydrophobically interacted with Met1211, Val1092, Ala1108, and Ile1084. Moreover, the phenoxy moiety (linker) interacted hydrophobically with Val1092, Ala1108, and Ieu1157. Next, the urea moiety formed HB with another highly conserved residue, Lys1110, located in the HB domain. Further, the benzothiazole ring formed a hydrogen bond with key residue Asp1222 in addition to hydrophobic interactions with Ile1130, Met 1131, and Ala1221.

Compound **7a** showed a docking energy of −9.58 kcal/mol. The carbamate substituent attached to the benzothiazole moiety of **7a** interacted with Arg1203 through a hydrogen bond and hydrophobically interacted with His1202 and Phe1200. Additionally, the benzothiazole moiety itself interacted with Met1131 (π-sulfur). Next, the phenoxy (linker) interacted with the highly conserved residue Lys1110 through a hydrogen bond and interacted hydrophobically with Met1131, Ala1226, Leu1157, and Phe1223 (π-π stacked) accordingly. Further, the pyrazine ring formed a hydrogen bond with the highly conserved key residue Met1160 in addition to hydrophobic interactions with Ile1084, Met1211, and Ala1108. Compound **12a** (*S* = −8.56 kcal/mol) exhibited the dual hydrogen bond interaction with key residue Met1160 through the nitrogen of the amide group present in the hinge region, as well as hydrophobic interaction with Tyr1159 through the methyl group. In addition, the benzimidazole moiety interacted hydrophobically with Ile1084, Val1092, Ala1108, and Met1211 (π-sulfur). Next, the phenyl linker hydrophobically interacted with Met1211, Leu1157, and Phe1223.

##### Molecular Dynamics Simulation

The results of the biological evaluation and molecular docking studies highlight compound **6b** as a promising dual EGFR/VEGFR-2 inhibitor and a potential anticancer agent. Accordingly, the crystal structure of VEGFR-2 in complex with sorafenib and compound **6b** was selected for molecular dynamics (MD) simulations. The most favorable binding conformation of compound **6b** within the VEGFR-2 active site was obtained from docking studies, emphasizing that the protein complex was subjected to a 200 ns MD simulation. The simulation used a standard force field and a water model. The systems were first minimized and then equilibrated. Production runs were carried out under a constant temperature and pressure.

Root mean square deviation (RMSD) analysis was performed to assess the stability of the protein–ligand complex. For the 4ASD complex, the RMSD remained between 1.5 and 2.0 Å throughout the 100 ns simulation. This shows that the structure remained stable throughout the simulation. For the 4ASD-**6b** complex, the RMSD initially remained low but gradually increased to approximately 3.0 Å after 40 ns. This means that the system had more movement. Still, it stayed below 3.0 Å, so the complex was stable. The extra movement may be due to how compound **6b** fits and binds (Figure 10).

**Figure 10:**
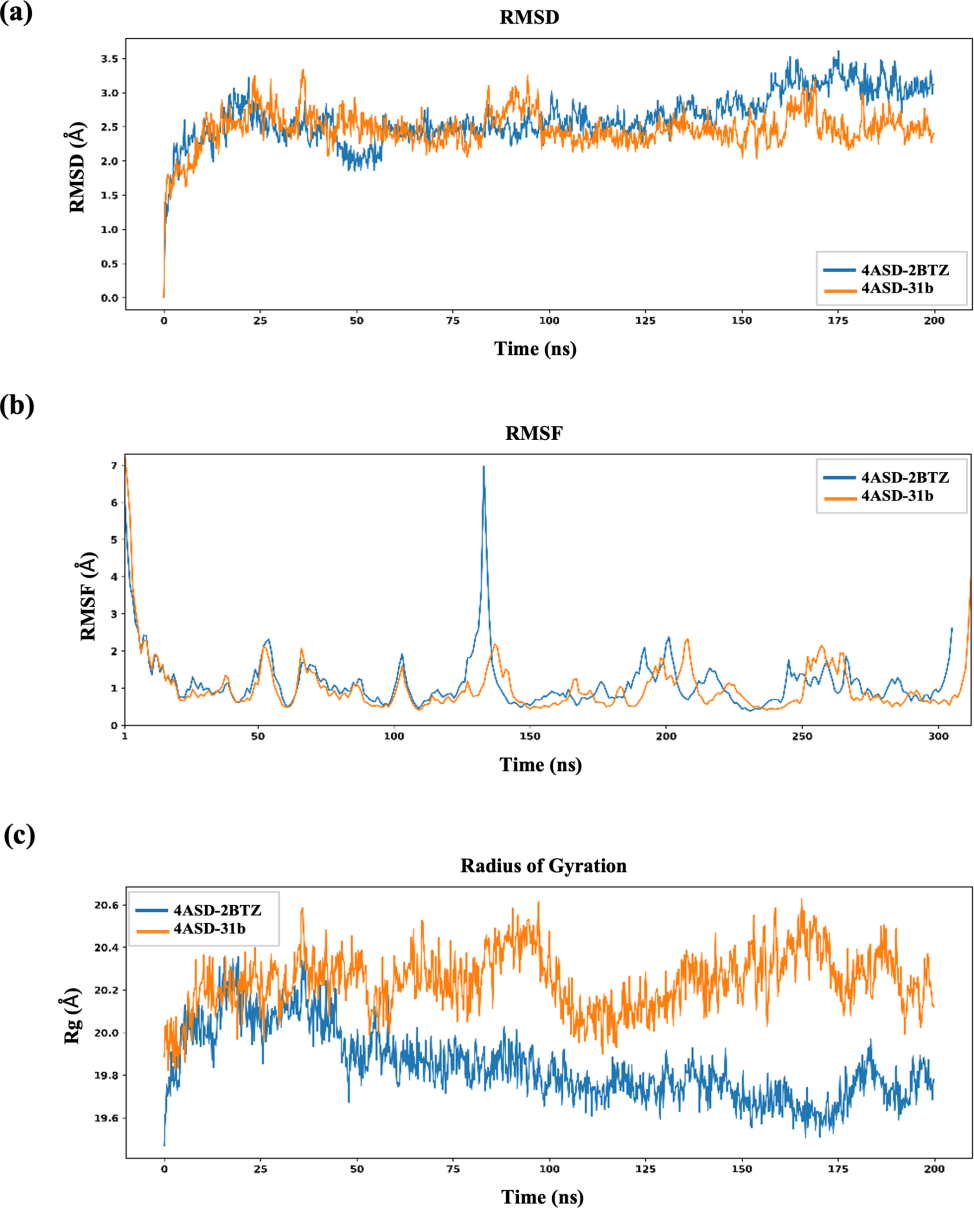
(a) RMSD, (b) RMSF, (c) and radius of gyration (*R*_g_) plots of sorafenib (blue) and **6b** (orange).

This indicates that the structure remained stable throughout the simulation. In the case of the 4ASD-**6b** complex, the RMSD initially remained low but gradually increased to approximately 3.0 Å after 40 ns, suggesting greater structural fluctuations. Nevertheless, as the RMSD remained below 3.0 Å, the complex can still be considered stable. The observed flexibility may be attributed to the binding mode and fit of compound **6b** within the active site.

RMSF measured how much each residue moved. In both the 4ASD and 4ASD-**6b** complexes, the N-terminal and C-terminal ends moved the most. The core residues moved very little, staying below 1.0 Å in most places. This shows that the main structural integrity remained stable. In the 4ASD-**6b** complex, certain loop regions had higher RMSF peaks, exceeding 2.0 Å in areas. This may be because of specific interactions established by the newly bound ligand. However, no significant structural instability was observed (Figure 10).

The radius of gyration indicates the compactness of the protein. For 4ASD, *R*_g_ stayed between 19.5 and 19.85 Å. This means that the protein did not spread out much. For 4ASD-**6b**, *R*_g_ ranged from 19.6 to 20.1 Å. It moved a bit more. This means that the protein was slightly more flexible when 2BTZ was bound. However, both systems stayed compact and stable overall (Figure 10).

The dynamic cross-correlation matrix (DCCM) was applied to examine the mobilities of different residues inside both complexes. DCCM looked at how residues moved together. In 4ASD, most of the correlated motions were close to the diagonal. This shows that local regions moving together. There were a few anticorrelated motions. This means that the structure moved stably. In 4ASD-**6b**, more off-diagonal patterns appeared. This means that distant residues moved together or in opposite ways. This may show that compound **6b** binding changes how the protein moves, maybe due to an induced fit (Figure 11).

**Figure 11:**
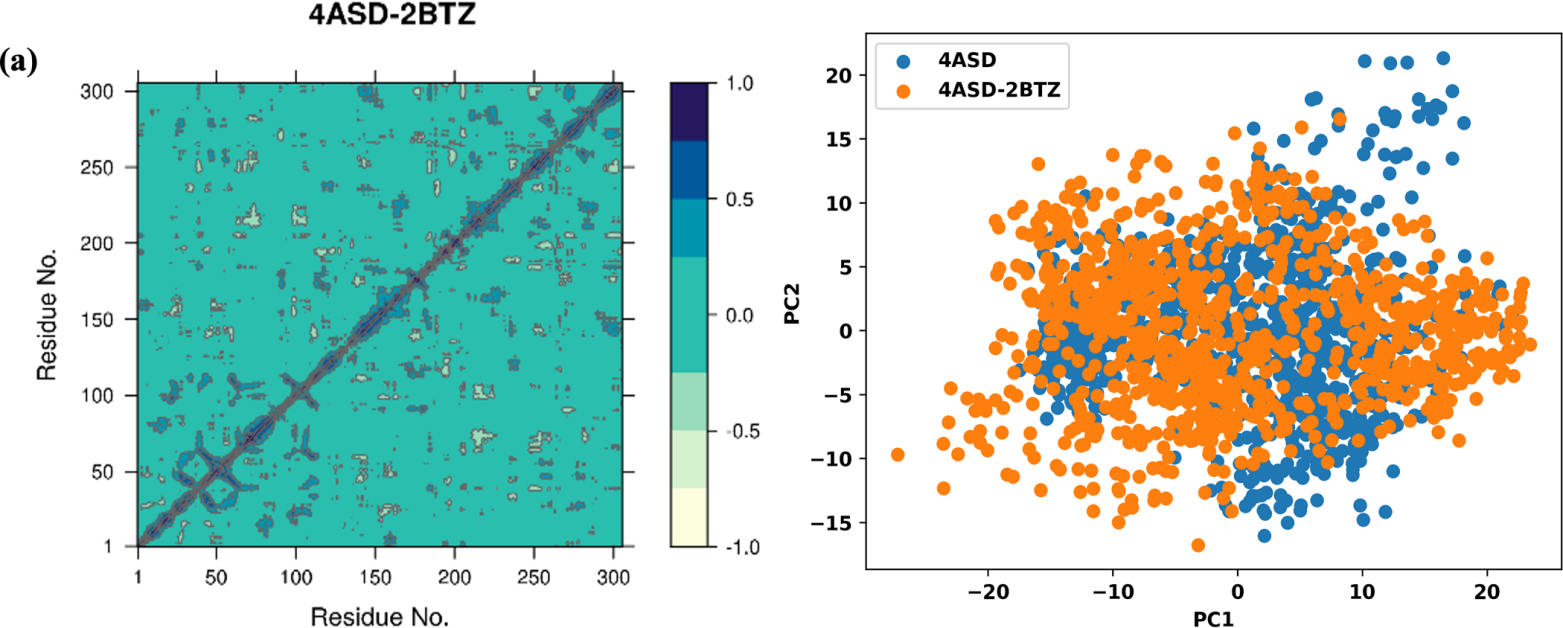
(a) DCCM of protein (4ASD = blue) and protein–ligand complex (4ASD-2BTZ = orange). (b) Principal component analysis (PCA) of compound **6b** (orange) and sorafenib (blue).

The free energy landscape is applied to quantify the energy change during MD simulation as a function of the principal components recommended in PCA. PCA showed the main movements in the proteins. The first two principal components, PC1 and PC2, were used. In 4ASD, the points were close together in the PCA plot. This means limited motion. In 4ASD-**6b**, the spread was wider across both PC1 and PC2. This shows more conformational changes. However, they were still there. Thus, both systems stayed in the same general shape, but 4ASD-**6b** was more dynamic (Figure 12).

**Figure 12:**
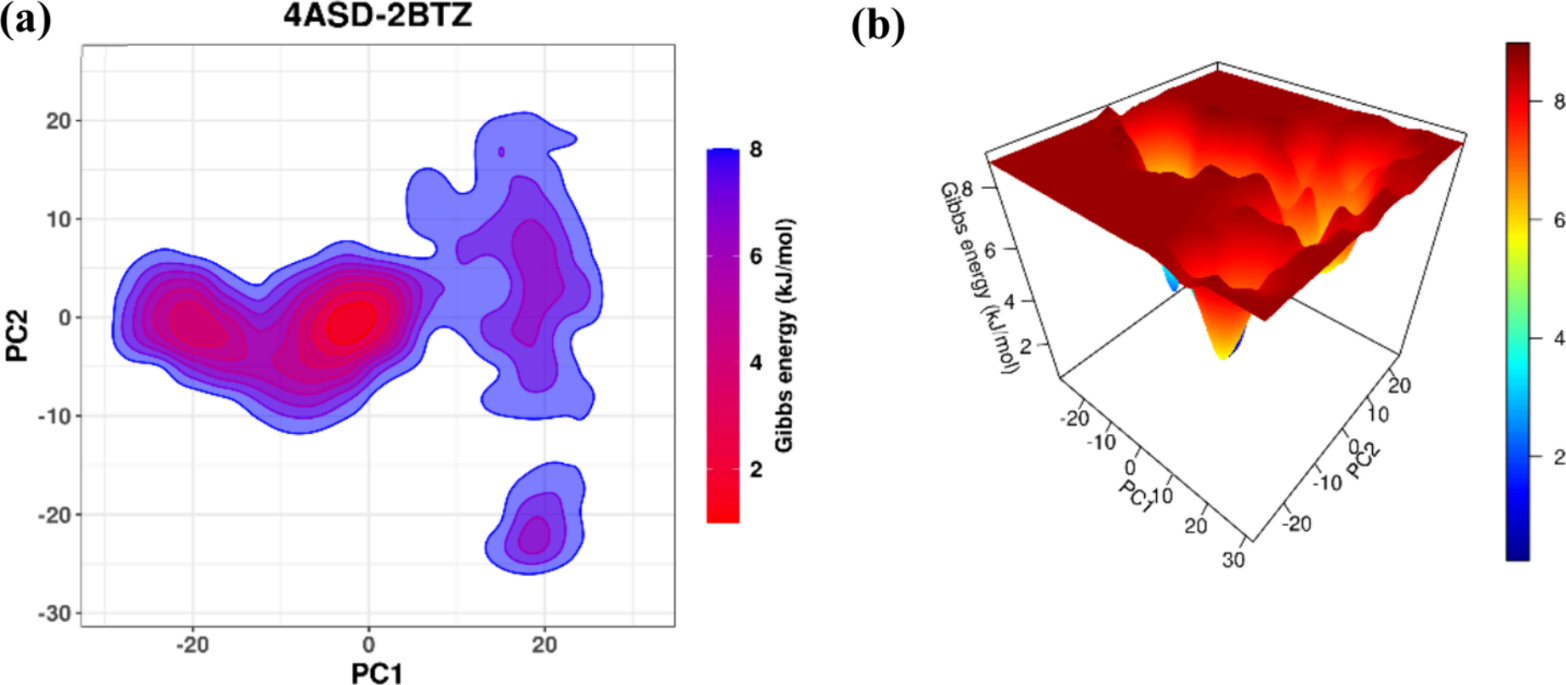
(a) Free energy landscape of the protein–ligand complex (4ASD**-6b**) and (b) 3D FEL of the protein–ligand complex.

2D FEL showed energy states over the simulation. In 4ASD, there was one deep energy well. This shows that the system stayed mostly in one stable shape. In 4ASD-**6b**, there were multiple energy wells. These were shallower and spread out more. Thus, the protein visited more shapes but still stayed stable (Figure 12). 3D FEL confirmed this. 4ASD had one steep, deep well with high barriers around it. This shows that the structure did not change much. 4ASD-**6b** had several shallow wells and gentle energy slopes, indicating that it moved between stable shapes more often. Therefore, the 2BTZ ligand allows the protein to be more flexible while still maintaining stability (Figure 12). This is also indicated by the interaction heatmaps in Figure 13.

**Figure 13:**
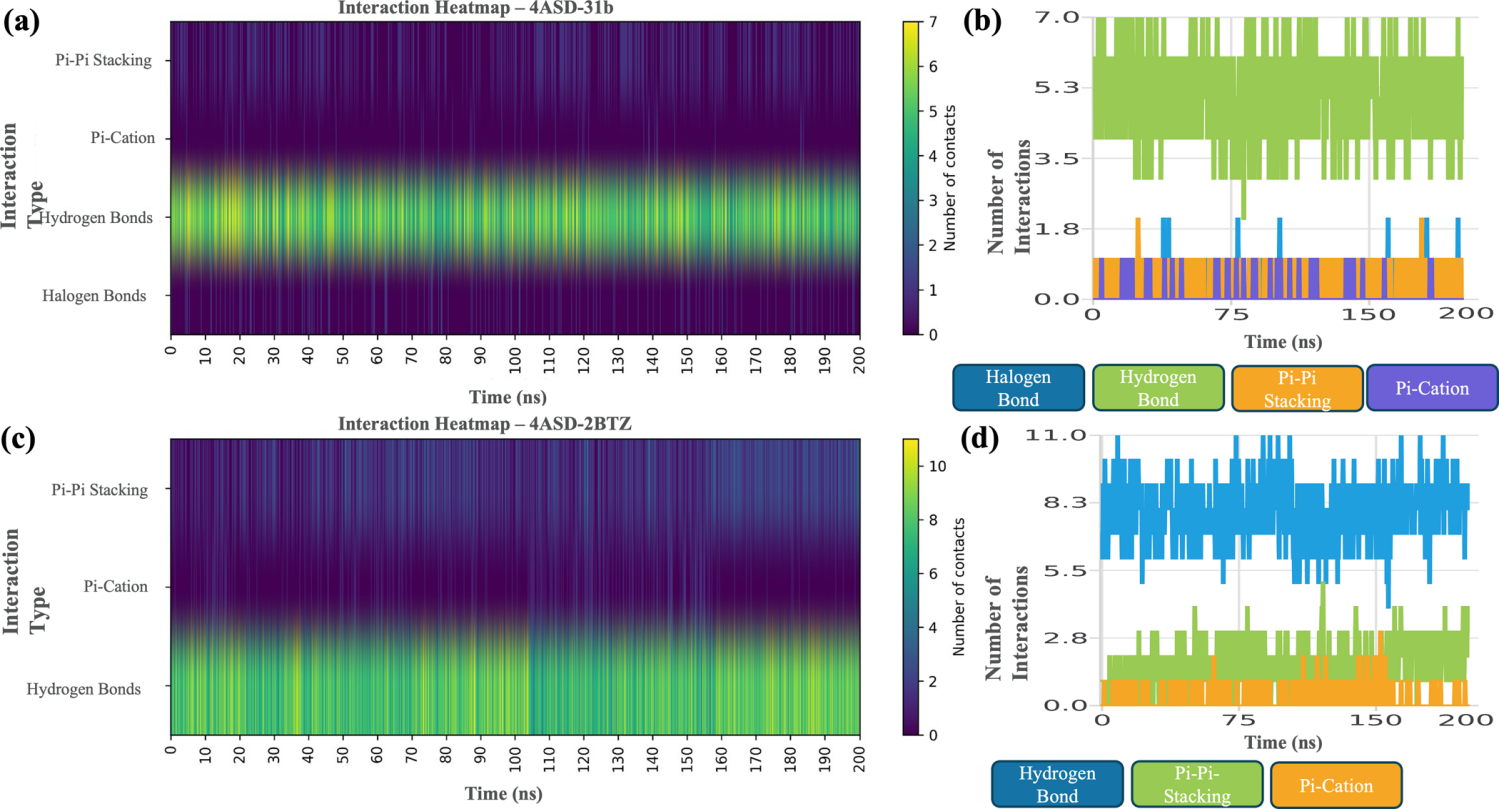
Interaction heatmaps of **4ASD-6b** (a, b) and **4ASD-2BTZ** (c, d).

## Conclusions

The present study describes the structural evolution of our earlier VEGFR-2 inhibitors by strategic substitution of a bicyclic 2-aminopyrimidine core with benzimidazole and benzothiazole scaffolds, coupled with diverse functionalities. In this study, two main scaffolds, 2-amino-6-hydroxybenzothiazole and methyl 2-(4-nitrophenyl)-1*H*-benzo­[*d*] imidazole-5-carboxylate, were used. On the right side, the 2-amino-6-hydroxy-benzothiazole core connected with the urea moiety via phenoxy and benzyloxy linkers, while its 2-amino group on the left-hand side was derivatized into carbamate and quinoline analogues. Conversely, nitro of the benzimidazole scaffold was converted to amine for urea formation, and modification of the ester functionality yielded amide derivatives. This fragment-merging approach successfully yielded a new series of multikinase inhibitors targeting c-Met, VEGFR-2, and EGFR.

The SAR analysis highlighted the urea/amide moiety, planar heteroaryl rings (thiazole, pyrazine, quinoline), and rigid aromatic linkers as critical elements of multikinase potential. Notably, compound **6b** (IC_5__0_ VEGFR-2 = 0.014 μM, EGFR = 0.94 μM, and c-Met = 3.58 μM) emerges as a potent inhibitor. Analogues **12a**, **12d**, **17**, and **18** exhibited the best antiproliferative potential against MCF7 and A549 cells, and **6b**, **7a**, **17**, and **18** showed inactivity against normal HEK-293 cells with IC_50_ values exceeding 100 μM (>100 μM) and indicated negligible/no cytotoxicity. Moreover, these analogues demonstrated antiangiogenic activity in the CAM assay through VEGFR-2 inhibition. Furthermore, **6b**, **7a**, **12a,** and **18** exhibit stable ATP-binding interactions with all three kinases, as indicated by molecular docking. Finally, these observations were reinforced by MD simulations (200 ns), where **6b** showed a stable binding mode as sorafenib yet verified enhanced conformational adaptability, as confirmed by PCA, DCCM, FEL, and interactions heatmap analyses. In summary, biological and molecular dynamic simulation data positioned **6b** as a potent multikinase inhibitor against c-Met, VEGFR-2, and EGFR. Currently, *in vivo* validation cannot be carried out as the experimental setup is not available. In the future, we intend to apply for financial support or find collaborating partners to test these lead analogues in xenograft tumor models and perform pharmacokinetic and toxicity assessments, further promoting them as potential anticancer agents.

## Materials and Methods

### General

All commercially available solvents were used without further purification. A digital balance was used to weigh the starting chemicals required for the reactions. ^1^H NMR and ^13^C NMR spectra were recorded in deuterated solvents (DMSO-*d*_6_, CDCl_3_) at 400 and 100 MHz, respectively, using a Bruker Avance III HD 400 MHz NMR spectrometer. Reaction progress was monitored using thin-layer chromatography (TLC) on precoated silica gel aluminum plates (Kieselgel 60, F254; Merck, Germany). A UV lamp (λ_max_ = 254 nm) was used to detect the chromatograms. Melting points (°C) of the synthesized compounds were measured by using a Stuart melting point apparatus and are uncorrected. An Elemental Vario analyzer (EI III CHN) was used for the elemental analysis. The key precursors **4a**, **4b**, **8**, and **9** were synthesized according to the reported procedures.

### General Method for the Synthesis of Intermediate **2a** and **2b**

A solution of 6-hydroxy-2-aminobenzothiazole **1** (25 mmol) and K_2_CO_3_ in DMF (35 mL), 1-chloro-4-nitrobenzene (25 mmol), or 4-nitrobenzyl bromide (25 mmol) was added. Then, at room temperature, the reaction mixture was stirred for almost 4 h. After the reaction was complete, the mixture was poured into ice-cold water (50 mL), and the obtained precipitates were filtered, washed, and recrystallized from ethanol to obtain **2a** and **2b,** respectively.

#### (4-Nitrophenoxy)­benzo­[*d*]­thiazole-2-amine (**2a**)

Brownish powder, yield 79%, 1H NMR (400 MHz, DMSO-*d*_6_) (ppm); δ 8.16 (d, *J* = 8.8 Hz, 2H), 7.70 (d, *J* = 8.8 Hz, 2H), 7.42 (d, *J* = 8.6 Hz, 1H), 7.21 (d, *J* = 2.4 Hz, 1H), 6.80 (dd, ^1^ *J* = 8.6 Hz, ^2^ *J* = 2.5 Hz, 1H), 6.57 (s, 2H, NH_2_).

#### 6-((4-Nitrobenzyl)­oxy)­benzo­[*d*]­thiazol-2-amine (**2b**)

Brown powder, Yield 82%, 1H NMR (400 MHz, DMSO-*d*_6_) (ppm); δ 8.16 (d, *J* = 8.8 Hz, 2H), 7.70 (d, *J* = 8.8 Hz, 2H), 7.42 (d, *J* = 8.6 Hz, 1H), 7.21 (d, *J* = 2.4 Hz, 1H), 6.80 (dd, ^1^ *J* = 8.6 Hz, ^2^ *J* = 2.5 Hz, 1H), 6.57 (s, 2H, NH_2_), 5.40 (s, 2H, CH_2_).

### General Synthetic Procedure of Intermediates **3a**, **3b**, **4a**, and **4b**

In the mixture of pyridine (45 mmol) and **2a** (20 mmol) or **2b** (20 mmol) in acetonitrile (40 mL), methyl chloroformate (30 mmol, 1.5 equiv) was added dropwise. Then, the reaction mixture was stirred overnight at room temperature. As the reaction was completed, the reaction mixture was poured onto crushed ice. The formed precipitates were filtered, washed with MeOH/cold water, and dried to provide **3a** and **3b**. To a solution of the **3a** (15 mmol) or **3b** (15 mmol) in EtOH (30 mL), SnCl_2_·2H_2_O (45 mmol) and conc. HCl (3 equiv) were added to the reaction mixture and allowed to reflux for 2 h. As the reaction was completed, indicated by TLC, the mixture was neutralized by adding aq. NaOH. The resulting precipitates were filtered, washed with H_2_O, and recrystallized from EtOH to get pure products **4a** and **4b**, respectively.

#### Methyl (6-(4-Nitrophenoxy)­benzo­[*d*]­thiazol-2-yl)­carbamate (**3a**)

Light brown powder, yield 83%, ^1^H NMR (400 MHz, DMSO-*d*_6_) (ppm); δ 10.51 (s, 1H, NH), 8.16 (d, *J* = 8.8 Hz, 2H), 7.70 (d, *J* = 8.8 Hz, 2H), 7.42 (d, *J* = 8.6 Hz, 1H), 7.21 (d, *J* = 2.4 Hz, 1H), 6.80 (dd, *J* = 8.6 Hz, 2.5 Hz, 1H), 3.77 (s, *J* = 3H, CH_3_).

#### Methyl 6-((4-Nitrobenzyl)­oxy)­benzo­[*d*]­thiazol-2-yl)­carbamate (**3b**)

Light brown powder, yield 78%, ^1^H NMR (400 MHz, DMSO-*d*_6_) (ppm); δ 10.51 (s, 1H, NH), 8.16 d, *J* = 8.8 Hz, 2H), 7.70 (d, *J* = 8.8 Hz, 2H), 7.42 (d, *J* = 8.6 Hz, 1H), 7.21 (d, *J* = 2.4 Hz, 1H), 6.80 (dd, ^1^ *J* = 8.6 Hz, ^2^ *J* = 2.5 Hz, 1H), 5.41 (s, 2H, CH_2_), 3.77 (s, *J* = 3H, CH_3_).

#### Methyl (6-(4-Aminophenoxy)­benzo­[*d*]­thiazol-2-yl)­carbamate (**4a**)

Bright yellow powder, yield 81%, ^1^H NMR (400 MHz, DMSO-*d*_6_) (ppm); δ 10.52 (s, 1H, NH), 8.18 (d, *J* = 8.7 Hz, 2H), 7.53 (d, *J* = 8.6, 1H), 7.23 (s, 1H), 7.10 (d, 8.7 Hz, 2H), 6.98 (dd, ^1^ *J* = 9.04 Hz, ^2^ *J* = 2.8 Hz, 1H), 5.18 (s,2H, NH_2_), 3.78 (s, 3H, CH_3_).

#### Methyl 6-((4-Aminobenzyl)­oxy)­benzo­[*d*]­thiazol-2-yl)­carbamate (**4b**)

Yellow powder, yield 81%, ^1^H NMR (400 MHz, DMSO-*d*_6_) (ppm); δ 10.51 (s, 1H, NH), 7.39 (d, *J* = 8.4 Hz, 1H), 7.22 (s, 1H), 7.11 (d, *J* = 8.2, 2H), 6.93 (dd, ^1^ *J* = 8.4 Hz, ^2^ *J* = 2.3 Hz, 2H), 5.40 (s, 2H, CH_2_), 5.17 (s, 2H, NH_2_), 3.77 (s, 3H, CH_3_).

### General Synthetic Method of Intermediates **8** and **9**

To a solution of ethyl-3,4-diaminobenzoate (30 mmol) and 4-nitrobenzaldehyde (30 mmol) in DMF (45 mL) was added Na_2_S_2_O_5_ (36 mmol) while continuously stirring. After the addition, the mixture was refluxed for 15 h at 100 °C until the reaction was completed as indicated by TLC. The reaction mixture was then cooled to room temperature, diluted with ethyl acetate (3 × 70 mL), dried (MgSO_4_), and concentrated under vacuum. The precipitates obtained were filtered and washed with DCM to obtain the desired intermediate **8**.^38^ To a solution of **8** (25 mmol) in EtOH (45 mL), SnCl_2_·2H_2_O (75 mmol) and conc. HCl (3 equiv) was added to the reaction mixture and allowed to reflux for 2 h. As the reaction was completed, indicated by TLC, the mixture was neutralized by adding aq. NaOH. The resulting precipitates were filtered, washed with H_2_O, and recrystallized from EtOH to get pure product **9**.

#### Methyl 2-(4-Nitrophenyl)-1*H*-benzo­[*d*]­imidazole-5-carboxylate (**8**)

Yellow crystal, yield 67%. HNMR (400 MHz, DMSO-*d*_6_): (ppm); δ 12.1 (s, 1H, NH), 8.28 (d, *J* = 8.84 Hz, 2H), 8.10 (d, *J* = 8.84 Hz, 2H), 7.86 (d, *J* = 1.72 Hz, 1H), 7.73 (dd, ^1^ *J* = 6.6 Hz, ^2^ *J* = 2.16 Hz, 1H), 7.58 (d, *J* = 6.84 Hz, 1H), 3.83 (s, 3H, CH_3_).

#### Methyl 2-(4-Aminophenyl)-1*H*-benzo­[*d*]­imidazole-5-carboxylate (**9**)

Bright yellow crystal, yield 81%. HNMR (400 MHz, DMSO-*d*_6_): δ 12.1 (s, 1H, NH), 8.28 (d, *J* = 8.84 Hz, 2H), 8.10 (d, *J* = 8.84 Hz, 2H), 7.86 (d, *J* = 1.72 Hz, 1H), 7.73 (dd,^1^ *J* = 6.6 Hz, ^2^ *J* = 2.16 Hz, 1H), 7.58 (d, *J* = 6.84 Hz, 1H), 5.23 (s, 2H, NH_2_), 3.83 (s, 3H, CH_3_).

### General Synthetic Method of Intermediate **13**

A mixture of 6,7-dimethoxyquinolin-4-ol (62.4 g, 30 mmol) in POCl_3_ (400 mL) was allowed to stir at 100 °C for about 5–8 h. After completion of the reaction, the mixture was cooled and the solvent was evaporated under reduced pressure. The obtained residues were slowly added to ice-cold water (400 mL), and a pH of 6 was maintained by using the K_2_CO_3_ solution to continue stirring for the next half an hour. The solid precipitates obtained were filtered, washed with H_2_O, and completely dried to get **13** as a product.

#### 4-Chloro-6,7-dimethoxyquinoline (**13**)

Light brown solid, Yield 83%. ^1^H NMR (400 MHz, DMSO-*d*_6_): (ppm); δ 8.60 (d, *J* = 5.2 Hz, 1H), 7.53 (d, *J* = 5.2 Hz, 1H), 7.35 (s, 1H), 7.43 (s, 1H), 3.96 (s, 3H), 3.95 (s, 3H).

### General Synthetic Method of Intermediates **14a** and **14b**

A solution of intermediate **13** (27 mmol) was reacted with either two different amines, **2a** or **2b** (32.4 mmol each), and allowed to reflux in the presence of 2-propanol (150 mL) for 5 h. After the reaction was completed, as indicated by TLC, the mixture was allowed to cool to room temperature, and the solvent was evaporated. Then, the obtained residues were purified by applying column chromatography eluting with CH_2_Cl_2_:CH_3_OH (10:1) to obtain title intermediates **14a** and **14b,** respectively.

#### *N*-(6,7-Dimethoxyquinolin-4-yl)-6-(4-nitrophenoxy)­benzo­[*d*]­thiazol-2-amine (**14a**)

Brown solid, yield 85%. ^1^H NMR (400 MHz, DMSO-*d*_6_): (ppm); δ 9.61 (s, 1H), 8.46 (d, *J* = 4.2 Hz, 1H), 8.29–8.23 (m, 2H), 7.61 (d, *J* = 7.8 Hz, 1H), 7.39 (d, *J* = 8.4 Hz, 2H), 7.23–7.17 (m, 2H), 7.02 (dd, ^1^ *J* = 7.6, ^2^ *J* = 2.1 Hz, 1H), 6.87 (d, *J* = 4.2 Hz, 1H), 3.91 (s, 3H), 3.85 (s, 3H).

#### *N*-(6,7-Dimethoxyquinolin-4-yl)-6-((4-nitrobenzyl)­oxy)­benzo­[*d*]­thiazol-2-amine (**14b**)

Brownish solid, yield 83%. ^1^H NMR (400 MHz, DMSO-*d*_6_): (ppm); ^1^H NMR (400 MHz, Chlo DMSO-*d*_6_) δ 9.61 (s, 1H), 8.46 (d, *J* = 4.2 Hz, 1H), 8.12–8.06 (m, 2H), 7.54 (ddd, ^1^ *J* = 7.8, ^2^ *J* = 1.8, ^3^ *J* = 1.0 Hz, 3H), 7.39 (d, *J* = 8.6 Hz, 2H), 7.04 (dd, ^1^ *J* = 7.7, ^2^ *J* = 2.2 Hz, 1H), 6.87 (d, *J* = 4.2 Hz, 1H), 4.95 (d, *J* = 0.9 Hz, 2H), 3.91 (s, 3H), 3.85 (s, 3H).

### General Synthetic Method of Intermediates **15a** and **15b**

To a stirring mixture of **14a** and **14b** (25 mmol each) in EtOH (45 mL), tin chloride dihydrate SnCl_2_·2H_2_O (75 mmol) and conc. HCl (3 equiv) were added and allowed to reflux for about 2 h. After reaction completion, indicated by TLC, aq. NaOH was added to neutralize the mixture. The resulting precipitates were filtered, washed with H_2_O, and recrystallized from EtOH to obtain pure products **15a** and **15b** subsequently.

#### 6-(4-Aminophenoxy)-*N*-(6,7-dimethoxyquinolin-4-yl)­benzo­[*d*]­thiazol-2-amine (**15a**)

Bright yellow solid, yield 80%. ^1^H NMR (400 MHz, DMSO-*d*_6_): δ 9.61 (s, 1H), 8.46 (d, *J* = 4.2 Hz, 1H), 7.61 (d, *J* = 7.8 Hz, 1H), 7.39 (d, *J* = 8.4 Hz, 2H), 7.02 (dd, ^1^ *J* = 7.5, ^2^ *J* = 2.2 Hz, 1H), 6.89–6.80 (m, 3H), 6.78–6.72 (m, 2H), 4.17 (s, 2H), 3.91 (s, 3H), 3.85 (s, 3H).

#### 6-((4-Aminobenzyl)­oxy)-*N*-(6,7-dimethoxyquinolin-4-yl)­benzo­[*d*]­thiazol-2-amine (**15b**)

Bright yellow solid, yield 79%. ^1^H NMR (400 MHz, DMSO-*d*_6_): δ 9.61 (s, 1H), 8.46 (d, *J* = 4.2 Hz, 1H), 7.53 (d, *J* = 7.8 Hz, 1H), 7.39 (s, 2H), 7.26–7.18 (m, 3H), 7.04 (dd, ^1^ *J* = 7.7, ^2^ *J* = 2.2 Hz, 1H), 6.87 (d, ^1^ *J* = 4.2 Hz, 1H), 6.66–6.60 (m, 2H), 4.95 (d, ^1^ *J* = 0.9 Hz, 2H), 4.36 (s, 2H), 3.91 (s, 3H), 3.85 (s, 3H).

### General Method for the Preparation of Target Compounds **6a**–**c**, **7a**–**c**, **11a**–**c**, **17**, and **18**

To the mixture of **4a**, **4b**, **9**, **15a**, and **15b** (20 mmol each) and pyridine (30 mmol) in ACN (40 mL), phenyl chloroformate (30 mmol) was added dropwise, and the mixture was stirred at room temperature overnight. As the reaction was completed, the reaction mixture was poured into crushed ice; the resulting precipitates were filtered out, washed with cold water, and dried to give **5a**, **5b**, **10**, **16a**, and **16b**, respectively. To the solution of **5a**, **5b**, **10**, **16a**, and **16b** (15 mmol) in 1,4 dioxane (30 mL) in Et_3_N (19.5 mmol), different amines were added and stirred at 60 °C for almost 6 h. The reaction mixture was cooled to RT, washed with diethyl ether, and recrystallized from ethanol to get urea derivatives **6a**–**c**, **7a**–**c**, **11a–d**, **17**,and **18**, respectively.

#### Methyl (6-(4-(3-(Pyrazin-2-yl)­ureido)­phenoxy)­benzo­[*d*]­thiazol-2 yl)­carbamate (**6a**)

White color solid, yield 62%, mp 190–192 °C, R_*f*_ = 0.57 (*n*-hexane/ethyl acetate 3:1) HPLC purity = 100% (C18 RP, MeOH/H_2_O – 95:5), *T*_R_ = 18.4 min. ^1^H NMR (400 MHz, DMSO-*d*_6_) δ 10.52 (s, 1H, NH), 10.22 (s, 1H, NH), 9.96 (s, 1H), 8.49 (s,1H), 8.27 (d, *J* = 7.32 Hz, 1H), 8.16 (d, *J* = 7.32 Hz, 1H), 7.53 (d, *J* = 8.6 Hz, 1H), 7.40 (d, *J* = 8.36 Hz, 2H), 7.21 (d, *J* = 2.28 Hz, 1H), 6.98 (dd, ^1^ *J* = 8.6 Hz, ^2^ *J* = 2.28 Hz, 1H), 6.89 (d, *J* = 8.36 Hz, 2H), 3.77 (s, 3H, CH_3_). ^13^C NMR (100 MHz, DMSO-*d*_6_) δ 163.0, 158.8, 156.5, 152.7, 152.2, 152,0, 145.0, 144.2, 134.9,134.0, 132.2, 130.5, 122.2 (2C), 121.2 (2C), 118.4, 117.2, 108.9, 52.1. LCMS: *m*/*z* = 437.1 [M + H]+; analysis calculated for C_20_H_16_N_6_O_4_S: C, 55.04; H, 3.70; N, 19.26; O, 14.66; S, 7.35; observed; C, 55.16; H, 3.68; N, 19.27.

#### Methyl (6-(4-(3-(5-Phenylthiazol-2-yl)­ureido)­phenoxy)­benzo­[*d*]­thiazol-2-yl)­carbamate (**6b**)

Light brownish color, solid, Yield 66%, mp 169–171 °C, R_*f*_ = 0.53 (*n*-hexane/Ethyl acetate 8:1) HPLC purity = 98.1% (C18 RP, MeOH/H_2_O – 95:5), *T*_R_ = 11.7 min. ^1^H NMR (400 MHz, DMSO-*d*_6_) δ 10.52 (s, 1H, NH), 10.23 (s, 1H, NH), 9.95 (s, 1H), 7.77–7.73 (m, 2H), 7.54 (d, *J* = 8.56 Hz, 1H), 7.47 (s, 1H), 7.39 (d, *J* = 8.36 Hz, 2H), 7.31–7.19 (m, 4H), 6.99 (dd, ^1^ *J* = 8.56 Hz, ^2^ *J* = 2.16 Hz, 1H), 6.88 (d, *J* = 8.36 Hz, 2H), 3.78 (s, 3H, CH_3_). ^13^C NMR (100 MHz, DMSO-*d*_6_) δ: 168.4, 163.2, 158.6, 156.9, 152.5, 151.5, 146.2, 138.9, 133.9, 132.3, 131.8, 129.7,129.3, 128.9 (2C), 125.7 (2C), 122.2 (2C), 120.7 (2C), 118.5, 117.4, 109.0, 52.6. LCMS: *m*/*z* = 518.6 [M + H]+; analysis calculated for C_25_H_19_N_5_O_4_S_2_: C, 58.02; H, 3.70; N, 13.53; O, 12.36; S, 12.39; observed: C, 58.1; H, 3.72; N, 13.50.

#### Methyl (6-(4-(3-(Benzo­[*d*]­thiazol-yl)­ureido)­phenoxy)­benzo­[*d*]­thiazol-2-yl)­carbamate (**6c**)

Light brown color solid, yield 60%, mp 213–215 °C, R_*f*_ = 0.54 (*n*-hexane/ethyl acetate 8:1) HPLC purity = 98.6% (C18 RP, MeOH/H_2_O – 95:5), T_R_ = 15.1 min. ^1^H NMR (400 MHz, DMSO-*d*_6_) δ 10.86 (s, 1H, NH), 10.51 (s, 1H, NH), 9.96 (s, 1H), 7.91 (d, *J* = 7.4 Hz, 1H), 7.64 (d, *J* = 7.36 Hz, 1H), 7.53 (d, *J* = 8.44 Hz, 1H), 7.40 (d, *J* = 8.12 Hz, 2H), 7.23 (d, *J* = 2.2 Hz, 1H), 7.1–7.08 (dt, *J* = 8.4 Hz, 2H), 6.98 (dd, ^1^ *J* = 8.44 Hz, ^2^ *J* = 2.2 Hz, 1H), 6.89 (d, *J* = 8.12 Hz, 2H), 3.77 (s, 3H, CH_3_). ^13^C NMR (100 MHz, DMSO-*d*_6_) δ: 163.5, 161.2, 158.5, 156.7, 152.9, 152.5, 148.4,145.2, 134.2, 132.7, 132.1, 125.4, 124.5, 123.7, 122.9, 122.0 (2C), 121.5 (2C), 118.9, 117.5, 108.4, 51.4. LCMS: *m*/*z* = 492.5 [M + H]+; analysis calculated for C_23_H_17_N_5_O_4_S_2_: C, 56.20; H, 3.49; N, 14.25; O, 13.02; S, 13.04; observed: C, 56.29; H, 3.47; N, 14.26.

#### Methyl (6-((4-(3-(Pyrazin-2-yl)­ureido)­benzyl)­oxy)­benzo­[*d*]­thiazol-2-yl)­carbamate (**7a**)

Off-white solid, yield 60%, mp 148–150 °C, R_*f*_ = 0.52 (*n*-hexane/ethyl acetate 3:1) HPLC purity = 100% (C18 RP, MeOH/H_2_O – 95:5), T_R_ = 10.49 min. ^1^HNMR (400 MHz, CDCl_3_) δ 10.51 (s, 1H, NH), 10.22 (s, 1H, NH), 9.99 (s, 1H), 8.49 (s,1H), 8.28 (d, *J* = 7.2 Hz, 1H), 8.18 (d, *J* = 7.2 Hz, 1H), 7.50 (d, *J* = 8.04 Hz, 1H), 7.40 (d, *J* = 8.36 Hz, 2H), 7.21 (d, *J* = 2.36 Hz, 1H), 7.14 (d, *J* = 8.04 Hz, 2H), 6.88 (dd, ^1^ *J* = 8.32 Hz, ^2^ *J* = 2.36 Hz, 1H), 5.40 (s, 2H, CH_2_), 3.73 (s, 3H, CH_3_). ^13^C NMR (100 MHz, CDCl_3_) δ 163.3, 158.3, 155.1, 152.1, 144.9, 143.7, 138.7,137.5, 134.3, 132.0, 130.8, 130.3, 129.5, (2C), 121.1 (2C), 118.1, 116.4, 109.2, 68.2, 51.6. LCMS: *m*/*z* = 451.5 [M + H]+; analysis calculated for C_21_H_18_N_6_O_4_S: C, 55.99; H, 4.03; N, 18.66; O, 14.21; S, 7.12; observed: C, 56.08; H, 4.01; N, 18.63.

#### Methyl (6-((4-(3-(Pyridin-3-yl)­ureido)­benzyl)­oxy)­benzo­[*d*]­thiazol-2-yl)­carbamate (**7b**)

White solid, yield 62%, mp 168–170 °C, R_*f*_ = 0.54 (*n*-hexane/ethyl acetate 5:1) HPLC purity = 99.1% (C18 RP, MeOH/H_2_O – 95:5), *T*_R_ = 10.01 min. ^1^H NMR (400 MHz, CDCl_3_) (ppm); δ 10.51 (s, 1H, NH), 10.22 (s, 1H, NH), 9.99 (s, 1H, NH), 8.92 (d, *J* = 2.36 Hz, 1H), 8.12 (dd, ^1^ *J* = 4.6 Hz, ^2^ *J* = 1.4 Hz, 1H), 7.97 (d, *J* = 8 Hz, 1H), 7.79 (dd, ^1^ *J* = 7.96 Hz, ^2^ *J* = 4.84 Hz, 1H), 7.50 (d, *J* = 8.12 Hz, 2H), 7.40 (d, *J* = 8.36 Hz, 2H), 7.22, (d, *J* = 2.2 Hz, 1H), 7.14 (d, *J* = 8.12 Hz, 2H), 6.89 (dd, ^1^ *J* = 8.36 Hz, ^2^ *J* = 2.2 Hz, 1H), 5.40 (s, 2H, CH_2_), 3.72 (s, 3H, CH_3_). ^13^C NMR (100 MHz, CDCl_3_) δ: 163.8, 158.6, 155.0, 145.0, 143.8, 142.8, 137.1, 135.5, 132.19, 130.2, 129.2, (2C), 125.6, 122.5, 121.3­(2C), 118.2, 116.6, 109.5, 68.3, 51.7. LCMS: *m*/*z* = 450.5 [M + H]+; analysis calculated for C_22_H_19_N_5_O_4_S C, 58.79; H, 4.26; N, 15.58; O, 14.24; S, 7.13; observed; C, 58.87; H, 4.24; N, 15.57.

#### Methyl (6-((4-(3-Benzylureido)­benzyl)­oxy)­benzo­[*d*]­thiazol-2-yl)­carbamate (**7c**)

Creamy color, solid, yield 59%, mp 154–156 °C, R_*f*_ = 0.55 (*n*-hexane/ethyl acetate 5:1) HPLC purity = 98.9% (C18 RP, MeOH/H_2_O – 95:5), *T*_R_ = 11.89 min. ^1^H NMR (400 MHz, CDCl_3_) (ppm); δ 10.52 (s, 1H, NH), 10.99 (s, 1H, NH), 7.98 (t, *J* = 5.92 Hz, *NH*(CH_2_), 1H), 7.50 (d, *J* = 8.16 Hz, 2H), 7.40 (d, *J* = 8.36 Hz, 2H), 7.34–7.27, (m, *J* = 2.2 Hz, 5H), 7.22 (d, *J* = 2.28 Hz, 1H), 7.14 (d, *J* = 8.16 Hz, 2H), 6.89 (dd, ^1^ *J* = 8.34 Hz, ^2^ *J* = 2.28 Hz, 1H), 5.40 (s, 2H, CH_2_), 4.78 (s, (*CH*_2_)­NH, 2H), 3.73 (s, 3H, CH_3_). ^13^C NMR (100 MHz, CDCl_3_) (ppm); δ 162.5, 158.9, 155.6, 142.9, 140.5, 138.1, 136.8, 132.4, 130.4, 129.1, 128.5, 127.2, 126.6, 121. 4, 117.8, 116.1, 108.0, 67.5, and 51.3. LCMS: *m*/*z* = 463.2 [M + H]+; analysis calculated for C_24_H_22_N_4_O_4_S: C, 62.32; H, 4.79; N, 12.11; O, 13.84; S, 6.93; observed; C, 62.41; H, 4.77; N, 12.13.

#### Methyl 2-(4-(3-(Pyrazin-2-yl)­ureido)­phenyl)-1*H*-benzo­[d]­imidazole-5-carboxylate (**11a**)

Light brown color solid, yield 60%, mp 181–183 °C, R_*f*_ = 0.44 (DCM/MeOH 4:1) HPLC purity = 98.5% (C18 RP, MeOH/H_2_O – 95:5), *T*_R_ = 7.8 min. ^1^H NMR (400 MHz, MeOD) δ 12.11 (s, 1H, NH), 10.24 (s, 1H, NH), 9.98 (s, 1H, NH), 8.48 (s, 1H, ArH), 8.28 (d, *J* = 7.56 Hz, 1H), 8.16 (d, *J* = 7.56 Hz, 1H), 8.03 (d, *J* = 8.4 Hz, 1H), 7.88 (d, *J* = 1.72 Hz, 1H), 7.77 (dd, ^1^ *J* = 6.84 Hz, ^2^ *J* = 1.72 Hz, 1H), 1H), 7.62 (dt, *J* = 8.4 Hz, 2H), 7.47 (d, *J* = 6.84 Hz, 1H), 3.83 (s, 3H).

^13^C NMR (100 MHz, MeOD) δ 168.5, 158.6, 154.3, 152.5, 144.2, 142.5, 141.2, 138.2, 134.7, 131.0, 129.2, 127.5, 127.0, 124.2, 118.7, 117.6, 112.7, 52.2 LCMS: *m*/*z* = 389.3 [M + H]+; analysis calculated for C_23_H_20_N_4_O_3_: C, 68.99; H, 5.03; N, 13.99; O, 11.99; observed; C, 69.08; H, 5.01; N, 13.97.

#### Methyl 2-(4-(3-(Benzo­[*d*]­thiazol-2-yl)­ureido)­phenyl)-1*H*-benzo­[*d*]­imidazole-5-carboxylate (**11b**)

Light brown color solid, yield 63%, mp 199–201 °C, R_*f*_ = 0.51 (*n*-hexane/Ethyl acetate 5:1) HPLC purity = 100% (C18 RP, MeOH/H_2_O – 95:5), *T*_R_ = 7.87 min. ^1^H NMR (400 MHz, DMSO-*d*_6_) δ 12.09 (s, 1H, NH), 9.79 (s, 1H, NH), 8.03 (d, *J* = 8.36 Hz, 2H), 7.96 (t, *J* = 5.76 Hz, 1H), 7.88 (d, *J* = 1.8 Hz, 1H), 7.77 (dd, ^1^ *J* = 6.92 Hz, ^2^ *J* = 1.8 Hz, 2H), 7.62 (d, *J* = 8.36 Hz, 1H), 7.47 (d, *J* = 6.92 Hz, 1H), 7.29 (dd, ^1^ *J* = 7.52 Hz, ^2^ *J* = 2.52 Hz, 2H), 7.21 (dd,^1^ *J* = 2.12 Hz, ^2^ *J* = 6.76 Hz, 3H) 4.72 (d, *J* = 5.67 Hz, 2H), 3.83 (s, 1H). ^13^C NMR (100 MHz, CDCl_3_) δ: 168.2, 158.8, 154.2, 142.5, 141.4, 138.9, 138.0, 129.0, 128.5 (2C), 127.7, 127.4 (2C), 126.9, 126.2 (2C), 124.0, 118.7 (2C), 117.5, 112.7, 52.2, 44.8. LCMS: *m*/*z* = 401.5 [M + H]+; analysis calculated for C_20_H_16_N_6_O_3_: C, 61.85; H, 4.15; N, 21.64; O, 12.36; observed; C, 61.93; H, 4.17; N, 21.61.

#### Methyl 2-(4-(3-Benzylureido)­phenyl)-*1H*-benzo­[*d*]­imidazole-5-carboxylate (**11c**)

Haze color solid, yield 61%, mp 140–142 °C, R_*f*_ = 0.58 (*n*-hexane/ethyl acetate 5:1) HPLC purity = 98.1% (C18 RP, MeOH/H_2_O – 95:5), *T*_R_ = 10.6 min. ^1^H NMR (400 MHz, MeOD) δ 12.09 (s,1H,NH), 9.79 (s, 1H, NH), 8.03 (d, *J* = 1.5 Hz, 2H), 7.97 (dd, ^1^ *J* = 7.4 Hz, ^2^ *J* = 1.5 Hz, 1H), 7.95 (dd, ^1^ *J* = 7.3, ^2^ *J* = 1.6 Hz, 1H), 7.88 (d, *J* = 1.5 Hz, 1H), 7.70 (dd, ^1^ *J* = 7.3 Hz, ^2^ *J* = 1.6 Hz, 1H), 7.66 (d, *J* = 7.5 Hz, 1H), 7.34 (td, ^1^ *J* = 7.4 Hz, ^2^ *J* = 1.6 Hz, 1H), 7.29 (td, ^1^ *J* = 7.4, ^2^ *J* = 1.6 Hz, 1H), 3.92 (s, 2H). ^13^C NMR (100 MHz, MeOD) δ: 166.99, 159.95, 154.92, 153.33, 151.47, 140.64, 140.58, 139.11, 131.23, 128.01, 126.06, 125.77, 125.74, 125.18, 124.65, 120.89, 120.33, 119.70, 119.22, 114.46, 52.11. LCMS: *m*/*z* = 444.4 [M + H]+; analysis calculated for C_23_H_17_N_5_O_3_S: C, 62.29; H, 3.86; N, 15.79; O, 10.82; S, 7.23; observed; C, 62.39; H, 3.84; N, 15.76.

#### Methyl 2-(4-(3-(Pyridin-3-yl)­ureido)­phenyl)-1*H*-benzo­[*d*]­imidazole-5-carboxylate (**11d**)

Yellow solid, yield 63%, m.p.185–192 °C, R_*f*_ = 0.51 (DCM/MeOH 3:1) HPLC purity = 98% (C18 RP, MeOH/H_2_O – 95:5), *T*_R_ = 3.89 min. ^1^H NMR (400 MHz, DMSO-*d*_6_) δ 12.11 (s, 1H, NH), 10.24 (s, 1H, NH), 9.97 (s, 1H, NH), 8.48 (s, 1H, ArH), 8.28 (d, *J* = 7.48 Hz, 1H), 8.16 (d, *J* = 7.48 Hz, 1H), 8.03 (d, *J* = 8.36 Hz, 2H), 7.88 (d, *J* = 1.8 Hz, 1H), 7.78 (dd, ^1^ *J* = 6.88 Hz, ^2^ *J* = 1.8 Hz, 1H), 7.62 (dt, *J* = 8.36 Hz, 2H), 7.47 (d, *J* = 6.88 Hz, 1H), 7.34 (t, *J* = 7.6 Hz, 1H), 3.93 (s, 3H). ^13^C NMR (100 MHz, DMSO-*d*_6_) δ: 166.99, 154.92, 153.3 144.64, 140.58, 140.1, 139.11, 131.23, 128.01 (2C), 125.77, 125.74, 125.18, 124.65, 120.89, 120.33, 119.70 (2C), 114.46, 52.11. LCMS: *m*/*z* = 388.1 [M + H]+; analysis calculated for C_20_H_17_N_7_O_2_: C, 65.11; H, 4.42; N, 18.08; O, 12.39; observed; C, 65.19; H, 4.40; N, 18.05.

### General Method for the Preparation of Benzimidazole-Based Urea Analogs **12a**–**d**

A solution of **11a**–**d** (20 mmol each) in ethanol was prepared by adding methylamine (30 mmol) dropwise and allowing the mixture to reflux for 5 h. As the reaction was completed, the reaction mixture was poured into crushed ice; the precipitates were filtered, washed with cold water, and recrystallized from ethanol to obtain the desired pure products **12a**–**d**.

#### *N*-Methyl-2-(4-(3-(pyrazin-2-yl)­ureido)­phenyl)-1*H*-benzo­[*d*]­imidazole-5-carboxamide (**12a**)

Yellow color solid, yield 68%, mp 200–202 °C, R_*f*_ = 0.54 (DCM/MeOH 3:1) HPLC purity = 98.1% (C18 RP, MeOH/H_2_O – 95:5), *T*_R_ = 10.6 min. ^1^H NMR (400 MHz, MeOD) δ 12.11 (s, 1H, NH), 10.24 (s, 1H, NH), 9.97 (s, 1H, NH), 8.70 (q, *J* = 5.64 Hz, 1H), 8.48 (s, 1H), 8.28 (d, *J* = 7.48 Hz, 1H), 8.16 (d, *J* = 7.48 Hz, 1H), 8.03 (d, *J* = 8.36 Hz, 2H), 7.88 (d, *J* = 1.8 Hz, 1H), 7.78 (dd, ^1^ *J* = 6.88 Hz, ^2^ *J* = 1.8 Hz, 1H), 7.62 (d, *J* = 8.36 Hz, 2H), 7.47 (d, *J* = 6.88 Hz, 1H), 2.90 (d, *J* = 5.64 Hz, 3H). ^13^C NMR (100 MHz, MeOD) δ 170.2, 159.5, 155.1, 152.1, 144.0, 142.3, 141.1, 137.7, 134.2, 131.0, 129.3, 127.9 (2C), 127.0, 124.3,118.8 (2C), 117.4, 112.8, 25.9. LCMS: *m*/*z* = 387.2 [M + H]+; analysis calculated for C_21_H_18_N_6_O_2_: C, 65.27; H, 4.70; N, 21.75; O, 8.28; observed; C, 65.35; H, 4.72; N, 21.73.

#### 2-(4-(3-(Benzo­[*d*]­thiazol-2-yl)­ureido)­phenyl)-*N*-methyl-1*H*-benzo­[*d*]­imidazole-5-carboxamide (**12b**)

Brown color solid, yield 61%, mp 209–212 °C, R_*f*_ = 0.43 (*n*-hexane/ethyl acetate 5:1) HPLC purity = 100% (C18 RP, MeOH/H_2_O – 95:5), *T*_R_ = 9.8 min. ^1^H NMR (400 MHz, DMSO-*d*_6_) δ 12.09 (s, 1H, NH), 10.24 (s, 1H, NH), 9.79 (s, 1H, NH), 8.70 (q, *J* = 5.64 Hz, 1H, NH), 8.03 (d, *J* = 8.36 Hz, 2H), 7.88 (d, *J* = 1.8 Hz, 1H), 7.77 (dd, ^1^ *J* = 6.92 Hz, ^2^ *J* = 1.8 Hz, 1H), 7.62 (d, *J* = 8.36 Hz, 2H), 7.47 (d, *J* = 6.92 Hz, 1H), 7.29 (dd, ^1^ *J* = 7.52 Hz, ^2^ *J* = 2.52 Hz, 2H), 7.21 (dd,^1^ *J* = 2.12 Hz, ^2^ *J* = 6.76 Hz, 2H), 2.90 (d, *J* = 5.64 Hz, 3H). ^13^C NMR (100 MHz, CDCl_3_) δ: 170.2, 158.8, 154.2, 151.4, 142.5, 141.4, 138.9, 138.0, 130.2, 129.0, 127.8 (2C), 127.4, 126.0 (2C), 124.5, 119.6, 118.7 (2C), 117.7­(2C), 112.9, 25.9. LCMS: *m*/*z* = 442.2 [M + H]+; analysis calculated for C_23_H_18_N_6_O_2_S: C, 62.43; H, 4.10; N, 18.99; O, 7.23; S, 7.25; observed; C, 62.34; H, 4.12; N, 19.02.

#### 2-(4-(3-Benzylureido)­phenyl)-*N*-methyl-1*H*-benzo­[*d*]­imidazole-5-carboxamide (**12c**)

Yellowish solid, yield 60%, mp 160–162 °C, R_*f*_ = 0.43 (*n*-hexane/ethyl acetate 3:1) HPLC purity = 98.1% (C18 RP, MeOH/H_2_O – 95:5), *T*_R_ = 10.6 min. ^1^H NMR (400 MHz, MeOD) δ 12.09 (s, 1H, NH), 10.24 (s, 1H, NH), 9.79 (s, 1H, NH), 8.69 (q, *J* = 5.72 Hz, 1H), 8.03 (d, *J* = 8.4 Hz, 2H), 7.96 (t, *J* = 5.76 Hz, 1H), 7.88 (d, 1.8 Hz, 1H), 7.77 (dd, ^1^ *J* = 6.84 Hz, ^2^ *J* = 1.72 Hz, 1H), 7.62 (d, *J* = 8.4 Hz, 2H), 7.47 (d, *J* = 6.84 Hz, 1H), 7.29–7.19 (m, 5H), 4.72 (d, *J* = 5.76 Hz, 2H), 2.90 (d, *J* = 5.68 Hz, 3H). ^13^C NMR (100 MHz, MeOD) δ 170.2, 157.6, 153.2, 142.7, 140.0, 139.5, 138.2, 130.1, 129.2, 127.8 (2C), 127.0 (2C), 126.0 (2C), 124.5, 123.0 (2C), 119.3, 117.7, 112.9, 44.81, 25.7. 168.25, 156.44, 153.27, 140.54, 140.31, 138.72, 138.16, 131.36, 128.53, 127.97, 127.60, 127.29, 125.07, 124.69, 119.70, 117.48, 113.66, 44.61, 26.69. LCMS: *m*/*z* = 400.4 [M + H]+; analysis calculated for C_23_H_21_N_5_O_2_: C, 69.16; H, 5.30; N, 17.53; O, 8.01; observed; C, 69.25; H, 4.28; N, 17.55.

#### *N*-Methyl-2-(4-(3-(pyridin-3-yl)­ureido)­phenyl)-1*H*-benzo­[*d*]­imidazole-5-carboxamide (**12d**)

Bright yellow solid, yield 67%, mp 208–210 °C, R_*f*_ = 0.41 (DCM/MeOH 3:1) HPLC purity = 98% (C18 RP, MeOH/H_2_O – 95:5), T_R_ = 5.89 min. ^1^H NMR (400 MHz, DMSO-*d*_6_) δ 12.11 (s, 1H, NH), 10.24 (s, 1H, NH), 9.97 (s, 1H, NH), 8.70 (q, *J* = 5.64 Hz, 1H), 8.48 (s, 1H), 8.28 (d, *J* = 7.48 Hz, 1H), 8.16 (d, *J* = 7.48 Hz, 1H), 8.03 (d, *J* = 8.36 Hz, 2H), 7.88 (d, *J* = 1.8 Hz, 1H), 7.78 (dd, ^1^ *J* = 6.88 Hz, ^2^ *J* = 1.8 Hz, 1H), 7.62 (d, *J* = 8.36 Hz, 2H), 7.47 (d, *J* = 6.88 Hz, 1H), 2.90 (d, *J* = 5.64 Hz, 3H). ^13^C NMR (100 MHz, CDCl_3_) δ: 170.2, 159.5, 155.1, 152.1, 144.0, 142.3, 141.1, 137.7, 134.2, 131.0, 129.3, 127.9, 127.0, 124.3, 118.8, 117.4, 112.8, 25.9. LCMS: *m*/*z* = 388.6 [M + H]+; analysis calculated for C_20_H_17_N_7_O_2_: C, 62.01; H, 4.42; N, 25.31; O, 8.26; observed; C, 62.11; H, 4.40; N, 25.28.

#### 1-(4-((2-((6,7-Dimethoxyquinolin-4-yl)­amino)­benzo­[*d*]­thiazol-6-yl)­oxy)­phenyl)-3-(pyrazin-2-yl)­urea (**17**)

Light yellow solid, yield 68%, mp 217–219 °C, R_*f*_ = 0.51 (*n*-hexane/ethyl acetate 3:1) HPLC purity = 100% (C_18_ RP, MeOH/H_2_O – 95:5), *T*_R_ = 5.7 min. ^1^H NMR (400 MHz, CDCl_3_) δ 10.15 (s, 1H, NH), 8.86 (s, 1H, NH), 8.71 (s, 1H, NH), 8.47–8.42 (m, 2H, ArH), 8.27 (d, *J* = 7.4 Hz, 1H), 8.16 (d, *J* = 7.4 Hz, 1H), 7.90 (d, *J* = 7.96 Hz, 1H), 7.62 (s, 1H), 7.52 (d, *J* = 8.5 Hz, 1H), 7.39 (d, *J* = 8.04 Hz, 2H), 7.31 (s, 1H), 7.19 (d, *J* = 2.16 Hz, 1H), 6.97 (dd, ^1^ *J* = 8.5, ^2^ *J* = 2.16 Hz, 1H), 6.86 (d, *J* = 8.04 Hz, 2H), 3.88 (s, 3H, OCH_3_), 3.83 (s, 3H, OCH_3_). ^13^C NMR (100 MHz, CDCl_3_): δ 163.9, 156.4, 153.6, 152.7, 152.3, 151.0, 149.9, 147.3, 146.2, 144.6, 142.7, 142.2, 137.6, 136.3, 133.3, 130.4, 122.5, 120.6, 118.4, 117.3, 115.5, 110.3, 107.4, 105.5, 104.4, 55.3, 54.0; LCMS: *m*/*z* = 566.1 [M + H]+; analysis calculated for C_29_H_23_N_7_O_4_S: C, 61.58; H, 4.10; N, 17.34; O, 11.31; S, 5.67 observed; C, 61.67; H, 4.08; N, 17.32.

#### 1-(4-(((2-((6,7-Dimethoxyquinolin-4-yl)­amino)­benzo­[*d*]­thiazol-6-yl)­oxy)­methyl)- phenyl)-3-(pyridin-3-yl)­urea (**18**)

Yellow color solid, Yield 65%, mp 204–206 °C, R_*f*_ = 0.54 (*n*-hexane/ethyl acetate 5:1) HPLC purity = 100% (C_18_ RP, MeOH/H_2_O – 95:5), *T*_R_ = 5.5 min. ^1^H NMR (400 MHz, DMSO-*d*_6_): δ 10.16 (s, 1H), 8.86 (s, 1H), 8.71 (s, 1H), 8.57 (d, *J* = 4.2 Hz, 1H), 8.44 (d, *J* = 7.84 Hz, 1H), 8.06 (d, ^1^ *J* = 8.32 Hz, 1H), 7.94–85 (m, 5H), 7.64–7.55 (m, 5H), 5.42 (s, 2H), 3.85, 3.83 (s, 6H, 2 × OCH_3_)), ^13^C NMR (100 MHz, DMSO-*d*_6_): δ 162.6, 155.5, 154.5, 151.9, 150.6, 149.7, 147.8, 146.9, 145.4, 143.8, 142.6, 139.9, 136.8, 130.7, 128.8, 127.3, 125.4, 118.7, 117.5, 115.4, 109.2, 107.0, 104.8, 70.8, 55.6, 54.4.6, 54.4. LCMS: *m*/*z* = 579.6 [M + H]+; analysis calculated for C_31_H_26_N_6_O_4_S: C, 64.35; H, 4.53; N, 14.52; O, 11.06; S, 5.54; observed; C, 64.44; H, 4.51; N, 14.78.

### Biological Evaluation

#### *In Vitro* VEGFR-2, EGFR, and c-Met Assays

The synthesized compounds were tested for their inhibitory potential against VEGFR-2, EGFR, and c-Met using target-specific assay kits. The assay kits configured in 96-well plates were supplied with VEGFR-2, EGFR, or c-Met kinase substrates, refined recombinant kinases, ATP, and appropriate assay buffers to support enzymatic reactions. The activity of the kinase was assessed using (Kinase-Glo) MAX, a luminescent detection agent. The assays were executed based on the outlined protocol: (i) Thaw 5× of kinase assay buffer together with Poly Glu/Tyr 4:1 substrate and ATP. (ii) For each of the *N* wells, prepare the master solution (25 μL per well) by blending 6 μL of Kinase assay buffer (5×), 1 μL of ATP (500 μM), 50× Poly­(Glu/Tyr) 4:1 (1 μL), and 17 μL of distilled water. (iii) Dispense inhibitor reagent (5 μL) to the wells marked as “Test Inhibitor”. Wells labeled as “Positive Control″ and “Blank” were given the same solution (5 μL) minus the inhibitor buffer or inhibitor. (iv) To make 3 mL of kinase buffer (1×), combine 2400 μL of water and 600 μL of kinase buffer (5×). (v) Deliver 20 μL of kinase buffer (1×) into the designated ″Blank″ well. (vi) Place the kinase to thaw, calculate the quantity of enzyme required for the test, and standardize the concentration of enzyme to 1 ng/μL using kinase buffer (1×). (vii) To activate the assay, deliver diluted kinases (20 μL) into “Positive Control” and Test Inhibitor Control″ wells. Then, incubation was maintained at 30 °C (45 min). (viii) Subsequently, in the span of 45 min, 50 μL of Kinase-Glo Max reagent was distributed to each well. Finally, luminescence intensity was detected through a microplate reader.^39,40^

#### *In Vitro* Antiproliferative Assay against MCF7 and A549 Cells

For the antiproliferative experiment, two tumor cell lines, namely, MCF7 and A549, were used. The cells were maintained in DMEM, supplemented with fetal bovine serum (10%) (FBS) and 1× antimycotic-antibiotic solution in a humidified incubator containing 5% CO_2_ at 37 °C. When the cells reached 70% confluency, they were subcultured into new T75 flasks. The antiproliferative effects of the test inhibitors were assessed using a sulforhodamine B (SRB) assay, as described previously. Next, the hemocytometer was utilized for counting the cells, which were then subsequently seeded at densities of 2,000 cells per well for A549 and 3,000 cells per well in 96-well plates accordingly. Following overnight incubation in a CO_2_-enriched incubator at 37 °C, the cells were treated for the next 72 h with nine serial 2-fold dilutions of inhibitors to calculate the GI_50_ values by taking DMSO as a solvent control. After post-treatment, at 4 °C, the cells were fixed using 10% trichloroacetic acid (TCA) for 2 h and then allowed to wash, air-dried, and stained at room temperature using 0.06% SRB dye. As cells were stained out, the plates were again washed by using 1% acetic acid and then air-dried. The protein-bound SRB dye was solubilized with a Tris base (pH 10.5), and the absorbance was measured at 490 nm with the help of a microplate reader. GI_5__0_ values were calculated by utilizing GraphPad Prism software.^41,42^

#### *In Vitro* Cytotoxicity Evaluation on Normal Human Embryonic HEK-293 Cells by the MTT Assay

The *in vitro* cytotoxicity of the newly synthesized compounds against the normal human kidney cell lines (HEK293) was estimated using an MTT assay.^43^ The cells were allowed to be cultured in DMEM medium and then treated with increasing concentrations of each compound (range from 0.125 to 512 μg/mL) followed by incubation for 48 h at 37 °C. After overnight incubation, the spent media were removed, and 10 μL of MTT reagent (3-(4,5-dimethylthiazol-2-yl)–2,5-diphenyltetrazolium bromide in PBS) was poured into 96-well cell culture plates and incubated at 37 °C for the next 4 h in a humidified incubator under dark conditions. Subsequently, 100 μL of DMSO was added and thoroughly mixed to dissolve the insoluble formazan crystals. The absorbance was measured at 570 nm using a micro-ELISA reader, and the IC_50_ value of the compounds was calculated accordingly.

### Method for the Acute Toxicity Test

The *in vivo* study was approved by the Ethics Committee (Approval No EC/1598/2024–8). Following the completion of the experiments, all animals were euthanized following the Animal Euthanasia guidelines established by the AVMA, ensuring humane treatment.

To prepare the dosing solution, compounds **6b** and **18** were initially dissolved in water and then diluted in 0.9% saline immediately before administration. Before the experiment, mice were fasted overnight. The following morning, each mouse received a single oral dose of compound **15b** or **35** via gavage, after which they were allowed free access to food and water. The animals were continuously monitored for the next 48 h to observe any signs of toxicity. The LD_5__0_ for compounds **6b** and **18** was determined using the up-and-down method, as outlined in the OECD guidelines. The study design involved increasing group sizes at each level: three mice at the first level followed by five, seven, nine, and ten mice at subsequent levels. The oral doses administered for compound **6b** were 1250, 1000, 750, 500, 250, 100, and 50 mg/kg of body weight (BW). While compound **18** doses employed were 1500, 1200, 900, 600, 300, 100, and 50 mg/kg BW. Throughout the experiment, mice were closely observed for any symptoms of toxicity immediately following compound administration.^44^

### Chicken Chorioallantois Membrane (CAM) Assay

The CAM assay was carried out in accordance with international guidelines on embryos by using fertilized chicken eggs before embryonic day 14. Subsequently, there was no need for ethical approval. However, all procedures were carefully performed to reduce any potential harm to the embryos.^45^ White Leghorn chicken eggs were purchased from the poultry trader in Lahore, Pakistan, and they were kept under an incubation period of 3 days. The next day of incubation, all eggs were sanitized with ethanol first, and then all eggs were again incubated vertically at 37 °C and 80% relative humidity for 24 h. Fertilized eggs were classified into four groups, in which three respective groups were treated with the tested compounds, while the fourth one served as a control group, receiving only ethanol therapy. Next, the window from the broad side of eggshells was broken up by using sterile scissors and arranging the sterile discs of tested analogues on sterile Whatman filter paper discs fixed with ethanol. Then, the windows were sealed with paper tape and allowed to incubate for 24 h. After the incubation period, windows were reopened, and the pictures for evaluating the angiogenesis process in the developing chick embryo’s chorioallantoic membrane (CAM).

### Docking Study Protocol

Docking studies were performed by using the Molecular Operating Environment (MOE 2016 08.02). VEGFR-2, EGFR, and c-Met crystal structures were downloaded (http://www.rcsb.org/pdb (PDB ID: 4ASD, PDB ID:1M17 and PDB ID:3LQ8)) from the Protein Data Bank. Water molecules were removed. All the three proteins and their crystallographic disorders, including alternate conformations and incomplete valence atoms, were corrected by applying the valence monitor options and alternate conformations, and protein energy minimization of the these three proteins (4ASD, 1M17, and 3LQ8) was performed by utilizing force fields (MMFF94) to assign charges and partial charges accordingly. The protein’s active binding site was then identified and prepared for molecular docking studies^46,47^

### Selection and Validation of the Docking Protocol

The active site of the binding region was determined using coordinates of the cocrystallized ligand as defined in the binding site instructions. The protein preparation step included 3D protonation, as well as energy minimization at the Amber10EHT force field with a convergence criterion of 0.1. The ligand structures were created utilizing the Molecular Builder segment present in MOE and minimized at the same force field but with a 0.00001 convergence gradient. For assessing the docking protocol, the redocking of the cocrystallized ligand had to be performed, and the root-mean-square deviation (RMSD) of the redocked structure was evaluated. All docking approaches with RMSD values approaching 1.0 Å were accepted. The Triangular Matcher algorithm was used for flexibility and induced fit docking to capture the conformational variability within the catalytic site. The computational estimator used to assess ligand binding affinity was the GBVI/WSA scoring function, which calculates the binding energy Δ*G* in kcal/mol, meaning that more negative values indicate more favorable interactions. Following the docking, the ligand–protein interaction analysis and 2D visualization of the binding poses were performed using BIOVIA Discovery Studio Visualizer (DSV).^48^

### Molecular Dynamics Simulation

Docking studies were carried out to determine the optimal binding interactions and conformations of the compounds, which were subsequently used for molecular dynamics simulations (MDS) using Schrödinger software. The selected docked poses were analyzed to explore protein–ligand interactions and assess their dynamic behavior. Maestro’s Wizard was used for preprocessing the protein–ligand complexes, including structural optimization and energy minimization. The System Builder tool was utilized to prepare all simulation systems. To ensure system neutrality, Na^+^ and Cl^–^ ions were added following solvation using the simple point-charge (SPC) water model within an orthorhombic box, maintaining a 10 Å buffer from the box edges. A physiological salt concentration of 0.15 M NaCl was introduced to mimic *in vivo* conditions.

Energy minimization was performed before simulations, and the potential energy of the complexes was further reduced under the NPT ensemble. Molecular dynamics simulations were conducted for 200 ns using the OPLS4 force field, maintaining a temperature of 300 K and a pressure of 1 atm. Short-range electrostatic interactions were computed by using the particle mesh Ewald method with a Coulomb cutoff radius of 9.0 Å. Water molecules were explicitly modeled using the SPC model. Pressure regulation was achieved via the Martyna–Tuckerman–Klein chain coupling method with a 2.0 ps coupling constant, while the temperature was controlled using the Nosé–Hoover chain thermostat. Trajectory data were recorded every 100 ps for postsimulation analysis. Root-mean-square deviation (RMSD) of the protein–ligand complex over time was used to assess the stability of the simulation. Both RMSD and root-mean-square fluctuation (RMSF) analyses were employed to evaluate the conformational changes from the initial structure throughout the simulation. The procedure for principal component analysis (PCA) is detailed in the cited methodological section.^49,50^

## Supplementary Material



## Notes

The NMR spectra and LC-MS chromatograms supporting the structural characterization of the compounds (PDF)
