# Supplementary material for: Structure-Guided Design of Benzothiazole and Benzimidazole-Based Urea Derivatives Curtailing Oncogenic Signaling via Concurrent Inhibition of VEGFR-2, EGFR, and c‑Met
Source: ACS Omega. 2026 Jan 21;11(4):6303–23. doi: 10.1021/acsomega.5c10972 (PMC12878492; doi:10.1021/acsomega.5c10972)
Supplement: Supplementary file 1 [file ao5c10972_si_001.pdf]

Supporting Information

Structure-Guided Design of Benzothiazole and Benzimidazole-Based Urea Derivatives Curtailing Oncogenic Signaling *via* Concurrent Inhibition of VEGFR-2, EGFR, and c-Met

Sadia Shaheen [1], Arshma Siddique [1], Ali Iftikhar [2], Amir Faisal [2], Hafiz Muzzammel Rehman [3], Ghulam Murtaza [4], Ayesha Tahir [1], Anees Saeed [1], Abbas Hassan [5,*], Umer Rashid [1,**]

[1] Department of Chemistry, COMSATS University Islamabad, Abbottabad Campus, 22060 Abbottabad, Pakistan.

[2] Department of Life Sciences, SBA School of Science and Engineering, Lahore University of Management Sciences, Lahore Cantt. 54792 Lahore, Pakistan

[3] School of Biochemistry and Biotechnology, University of the Punjab, Lahore, Punjab, Pakistan

[4] Department of Pharmacy, COMSATS University Islamabad, Lahore Campus, Lahore, Pakistan

[5] Department of Chemistry, College of Science, United Arab Emirates University, Al Ain, 15551, Abu Dhabi, United Arab Emirates

Corresponding authors

Abbas Hassan*: abbashassan@uaeu.ac.ae

Umer Rashid**; umerrashid@cuiatd.edu.pk

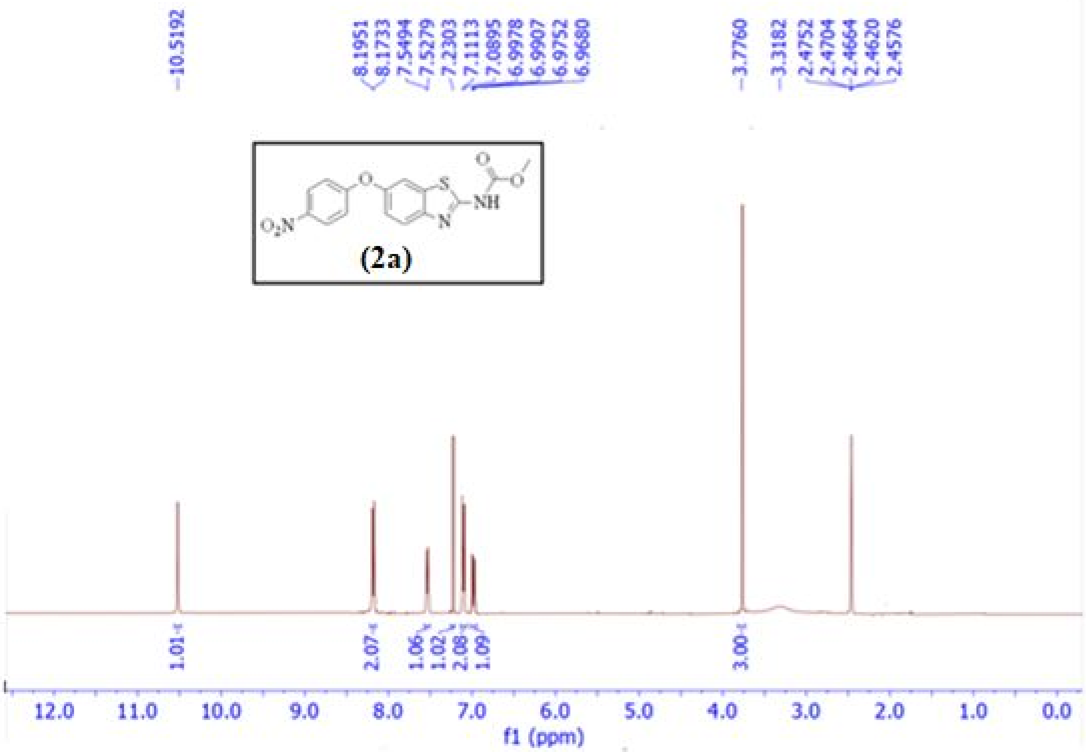

Figure S-1. 1H NMR (400 MHz, DMSO-*d6*) spectrum of compound **2a**

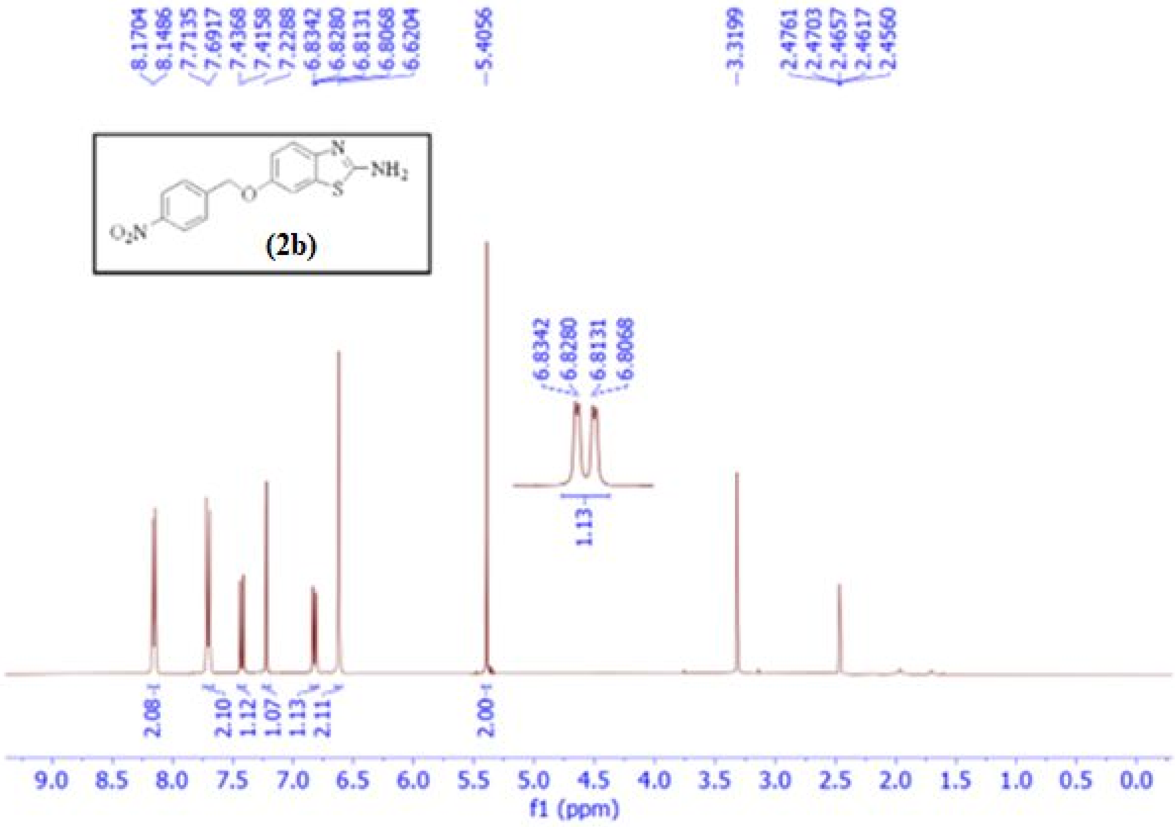

Figure S-2. 1H NMR (400 MHz, DMSO-*d6*) spectrum of compound **2b**

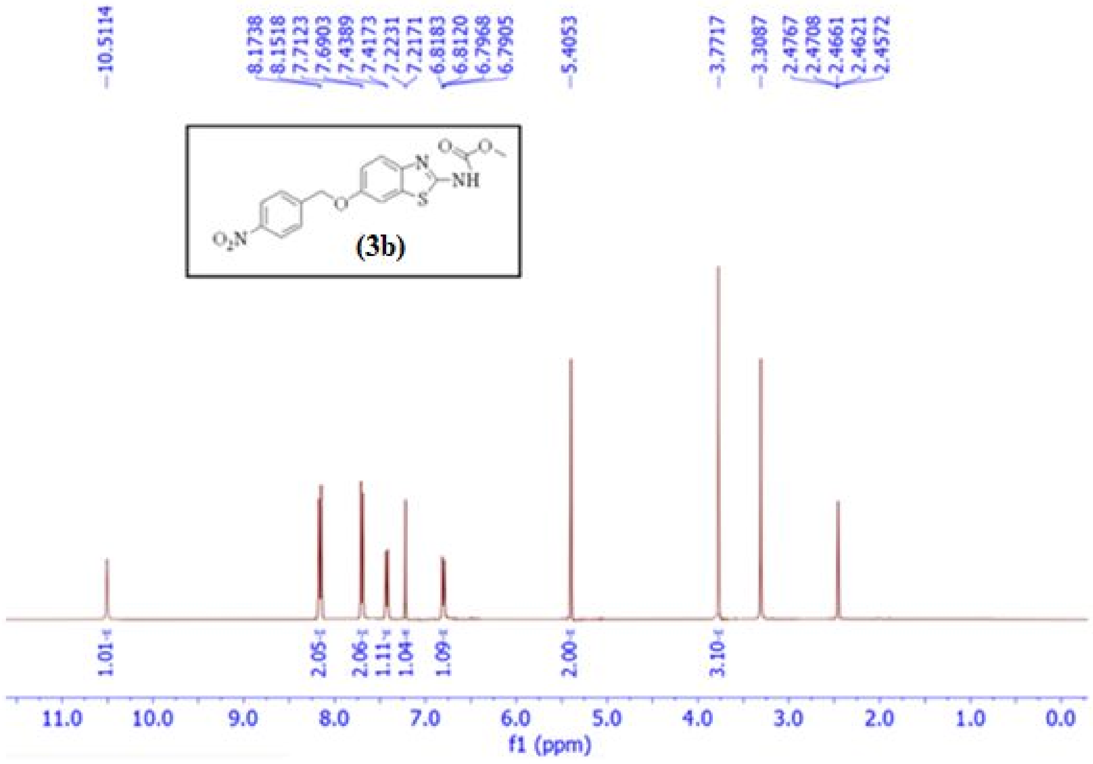

Figure S-3. 1H NMR (400 MHz, DMSO-*d6*) spectrum of compound **3b**

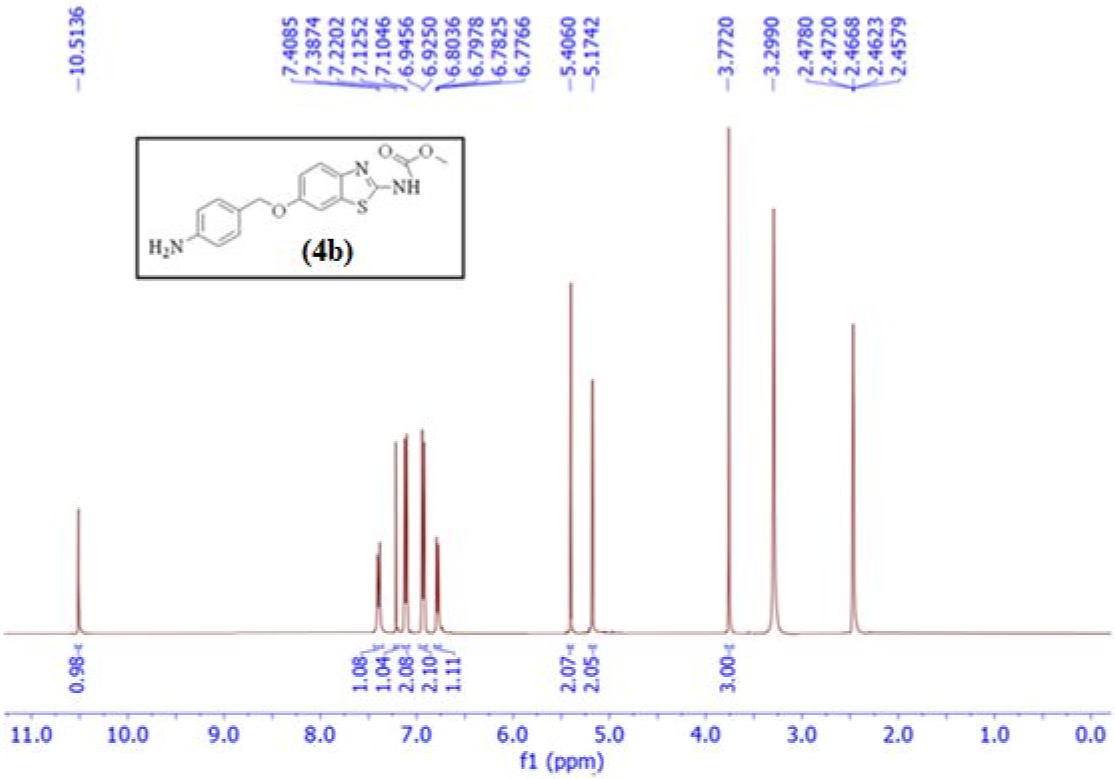

Figure S-4. 1H NMR (400 MHz, DMSO-*d6*) spectrum of compound **4b**

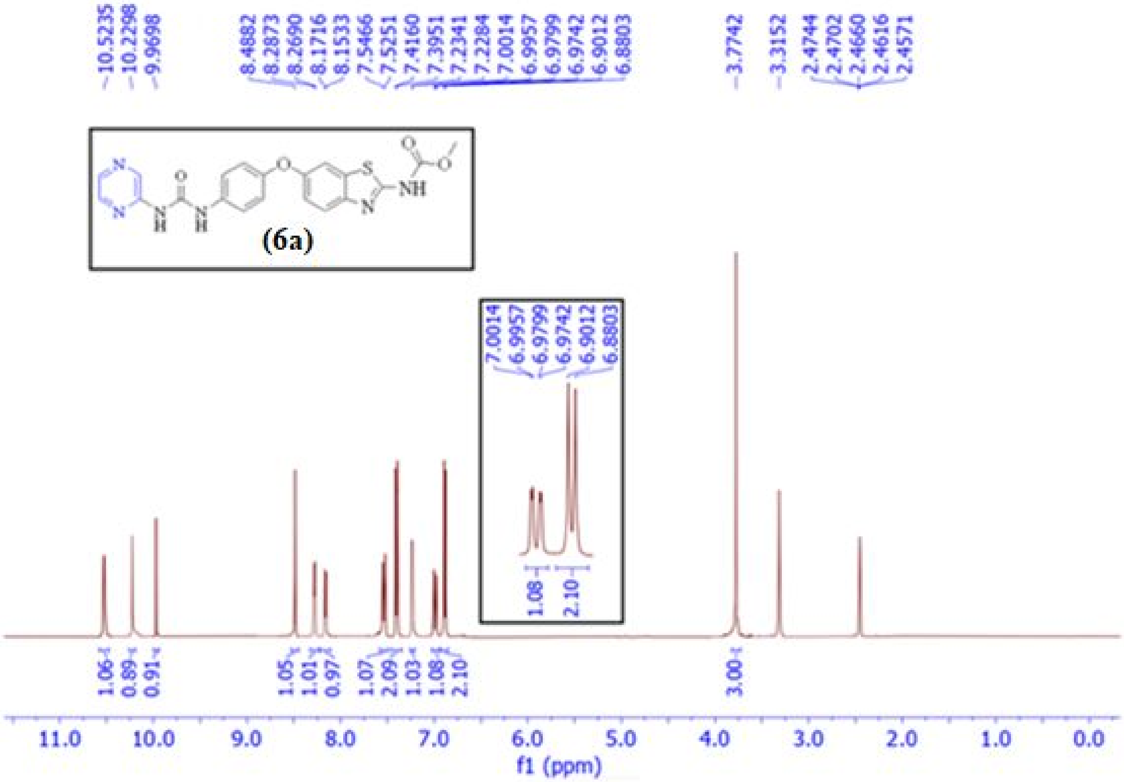

Figure S-5. 1H NMR (400 MHz, DMSO-*d6*) spectrum of compound **6a**

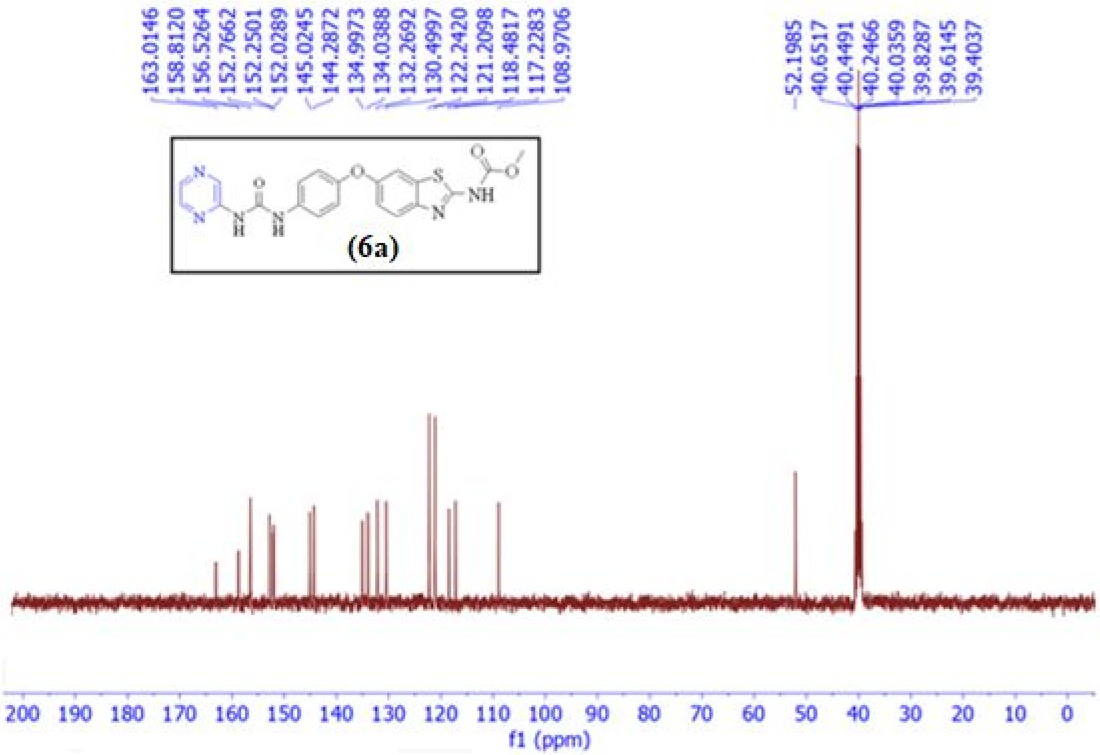

Figure S-6. ^{13}C NMR (100 MHz, DMSO-*d6*) spectrum of compound **6a**

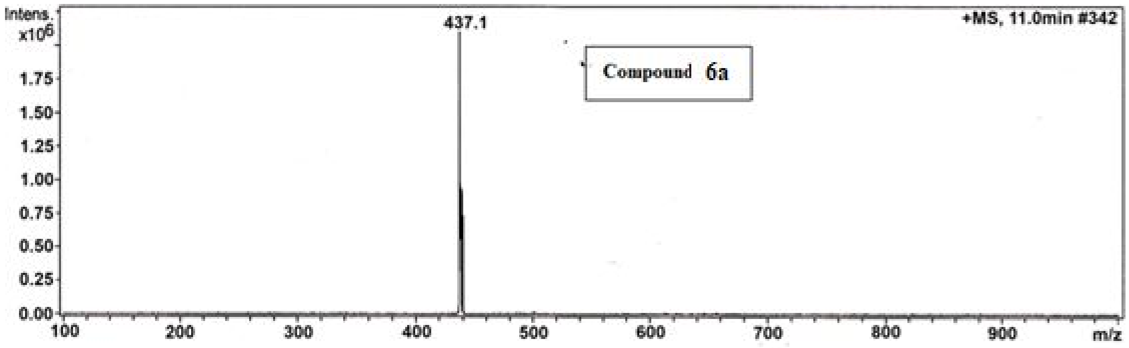

Figure S-7. LC-MS chromatogram of compound **6a**

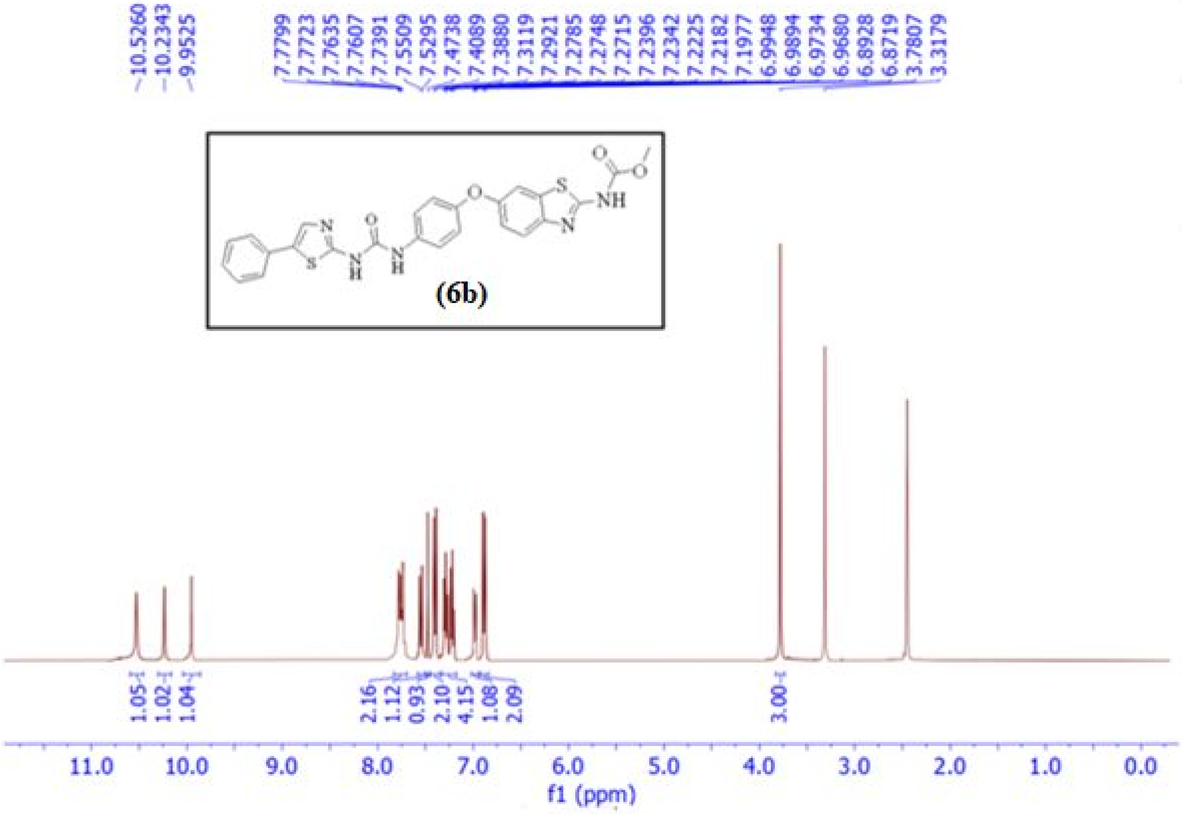

Figure S-8. 1H NMR (400 MHz, DMSO-*d6*) spectrum of compound **6b**

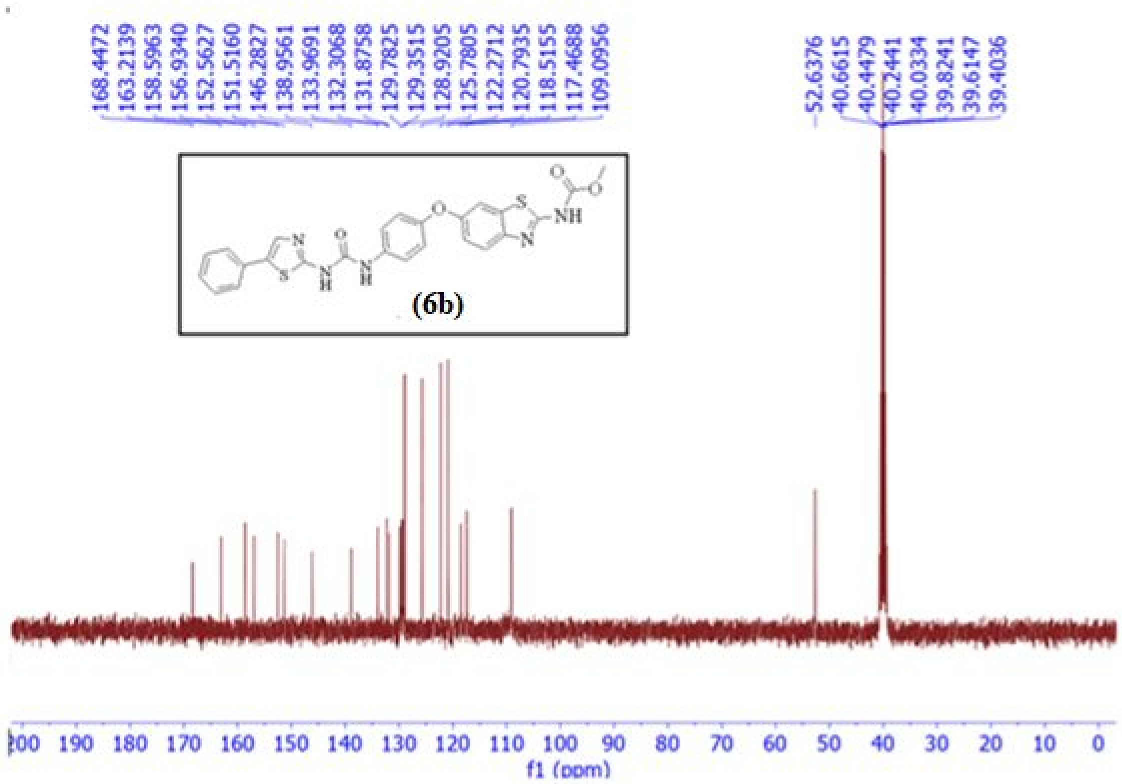

Figure S-9. ^{13}C NMR (100 MHz, DMSO-*d6*) spectrum of compound **6b**

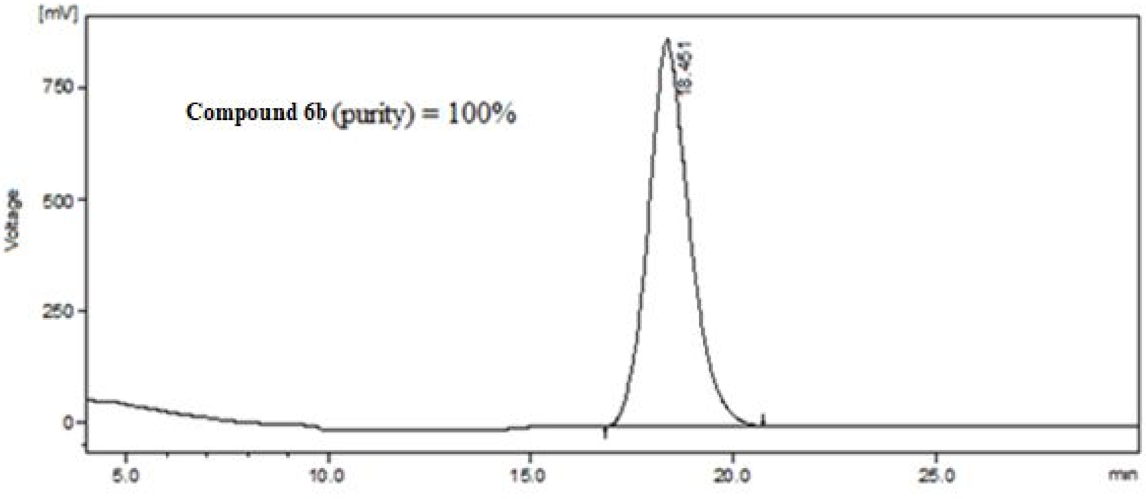

Figure S-10. HPLC chromatogram of compound **6b**

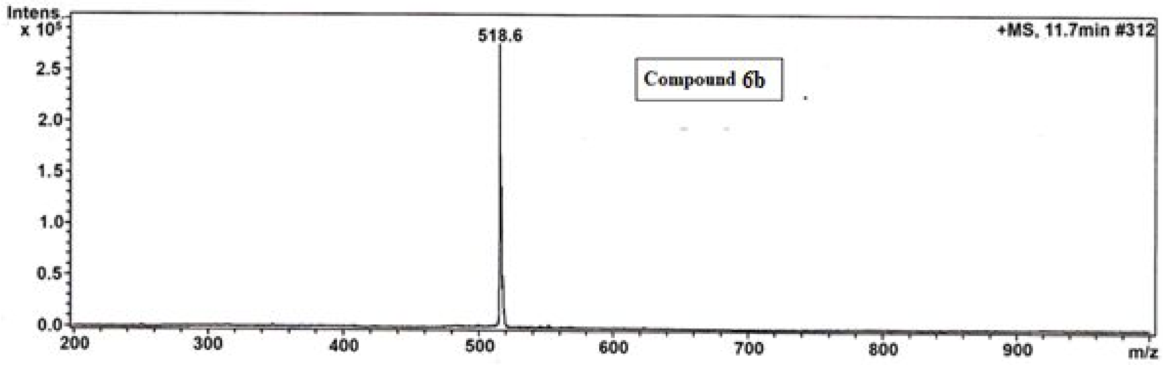

Figure S-11. LC-MS chromatogram of compound **6b**

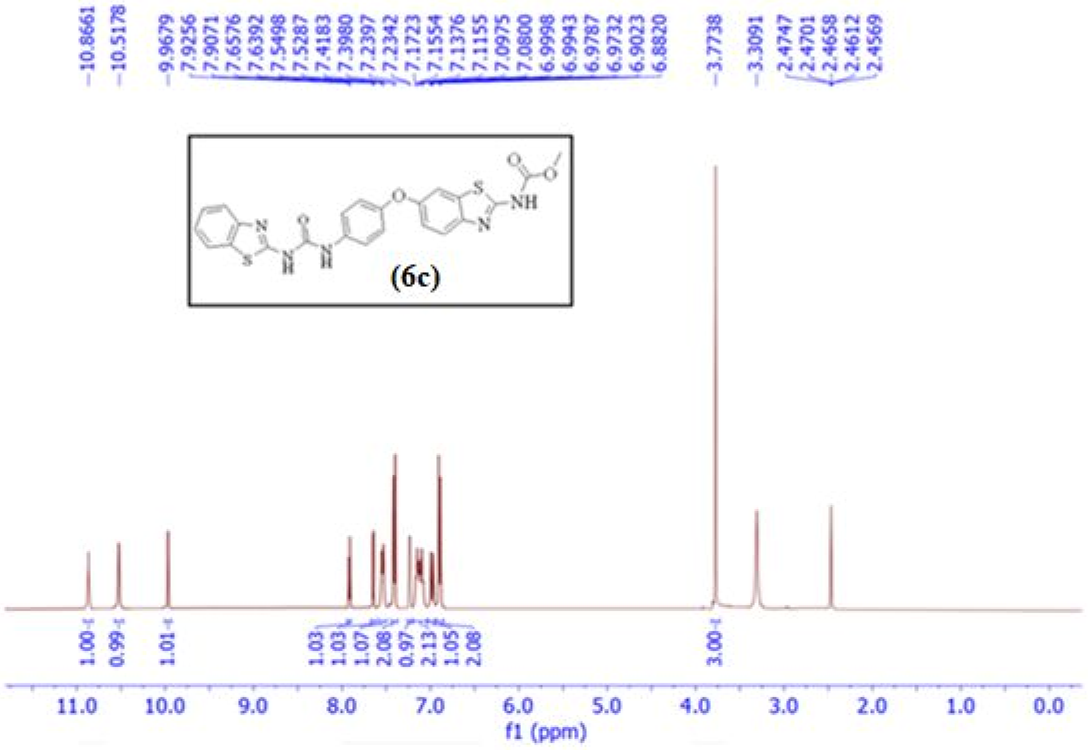

Figure S-12. 1H NMR (400 MHz, DMSO-*d6*) spectrum of compound **6c**

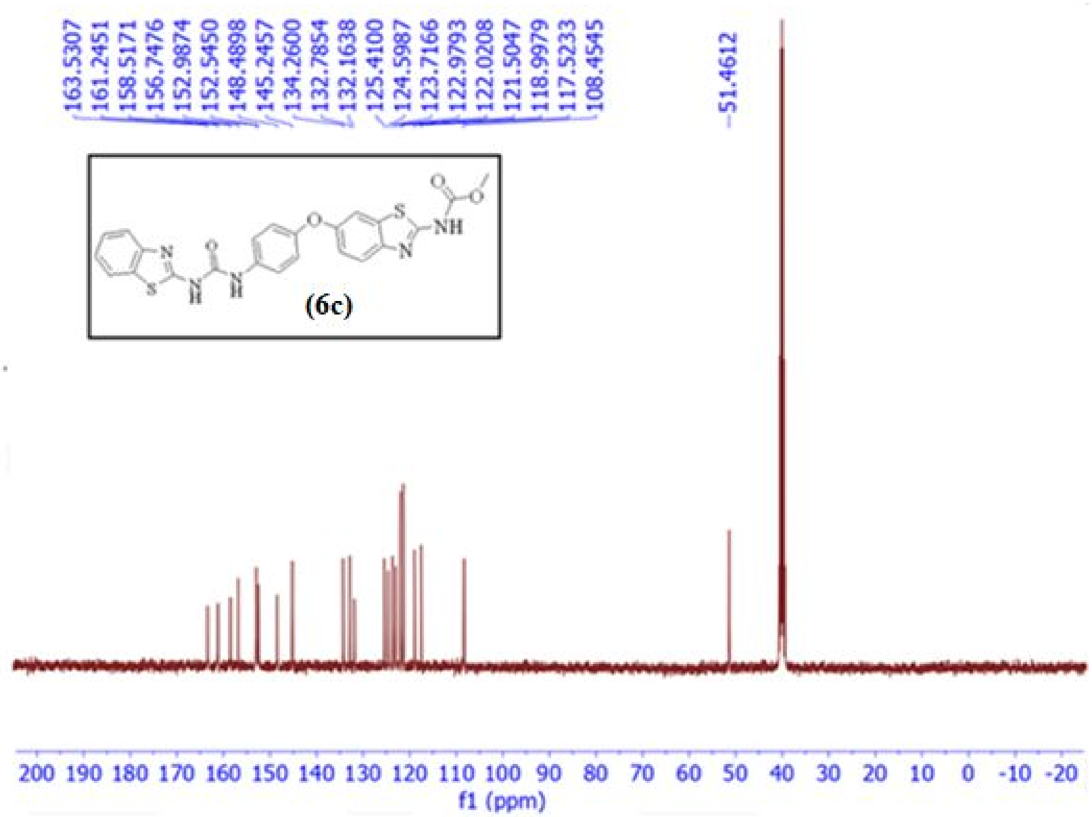

Figure S-13. ^{13}C NMR (100 MHz, DMSO-*d6*) spectrum of compound **6c**

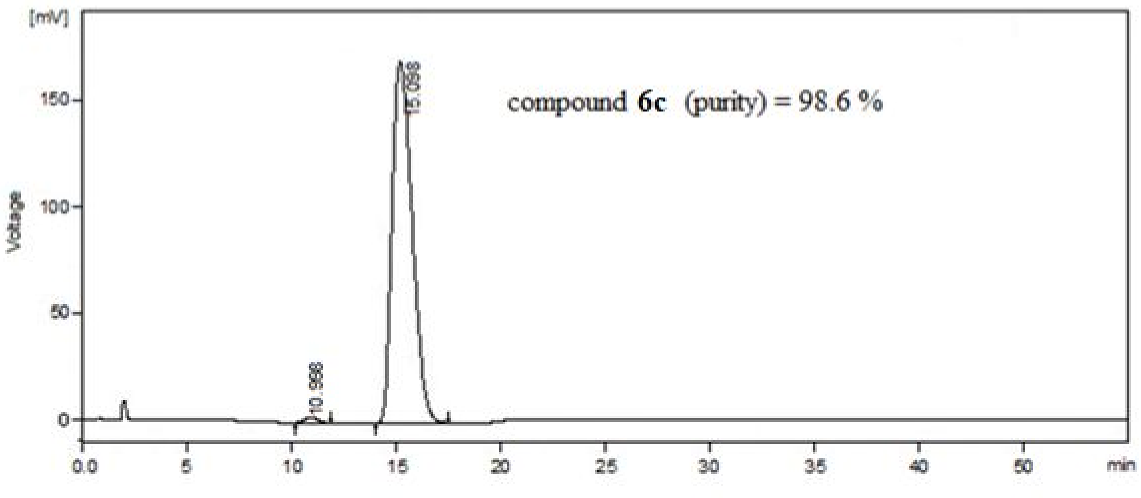

Figure S-14. HPLC chromatogram of compound **6c**

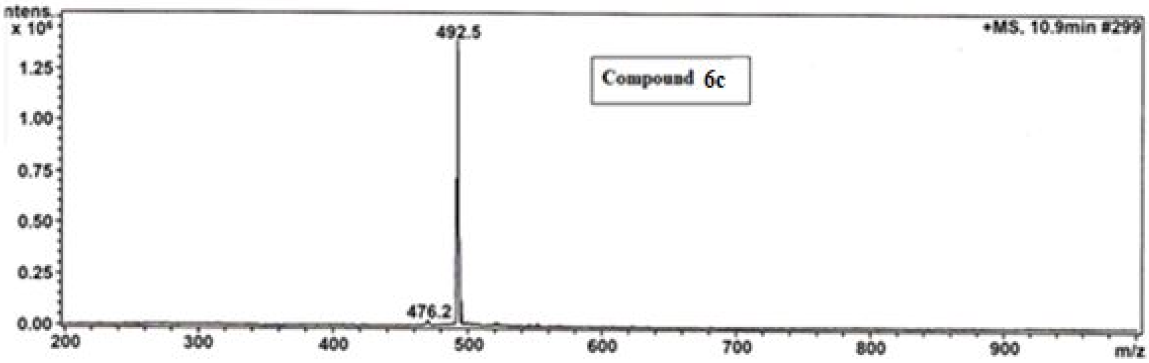

Figure S-15. LC-MS chromatogram of compound **6c**

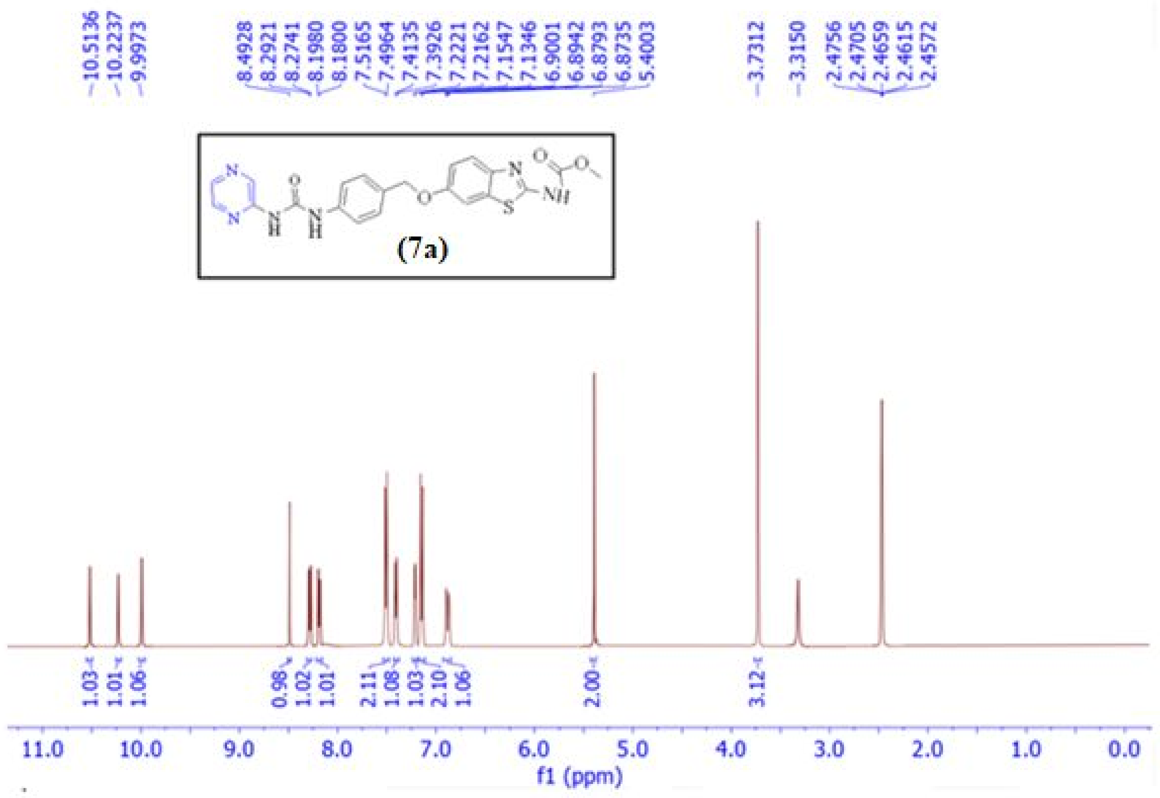

Figure S-16. 1H NMR (400 MHz, DMSO-*d6*) spectrum of compound **7a**

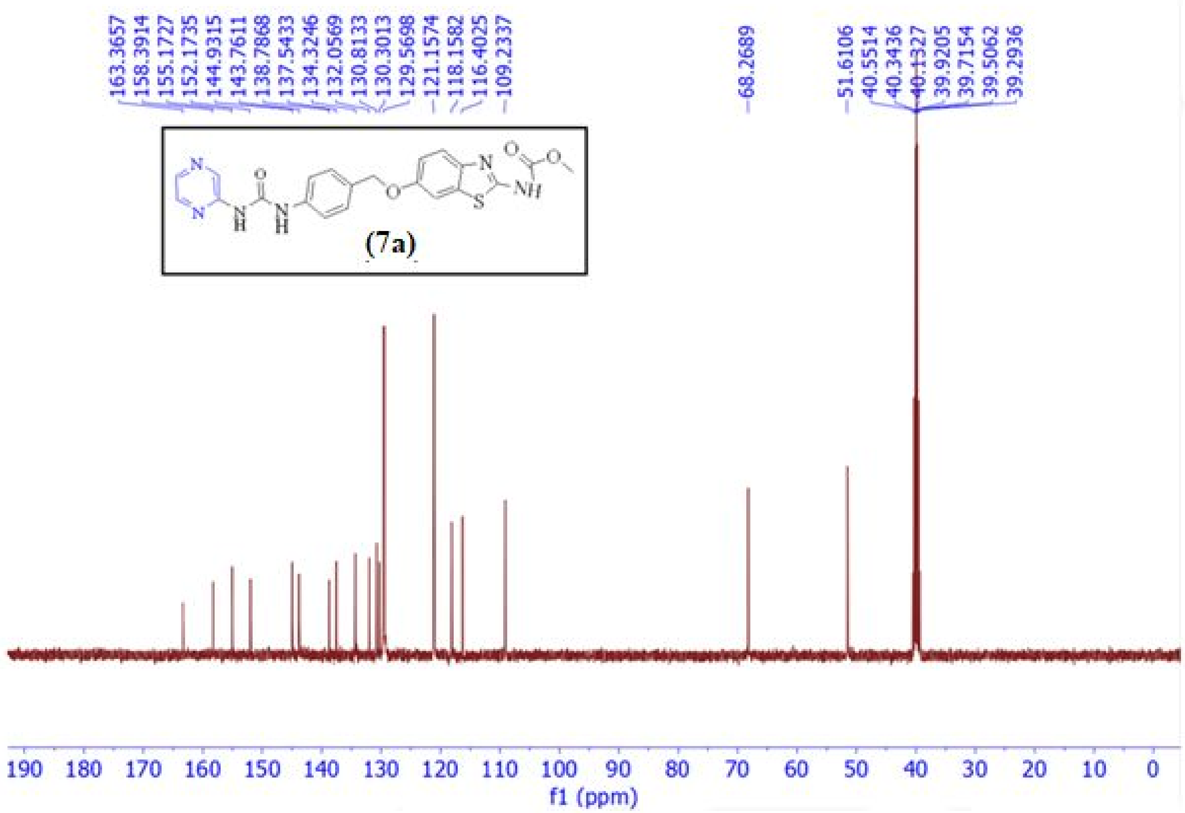

Figure S-17. ^{13}C NMR (100 MHz, DMSO-*d6*) spectrum of compound **7a**

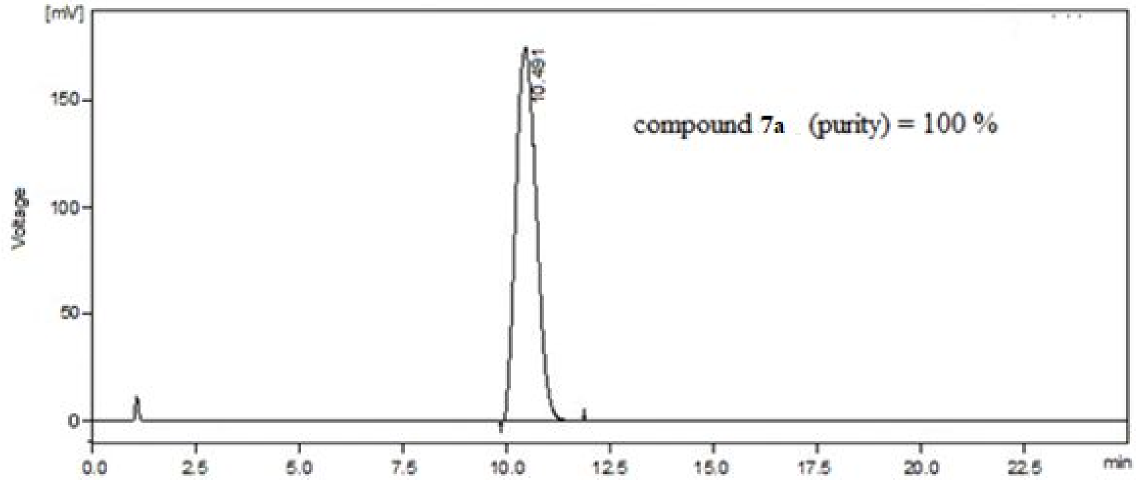

Figure S-18. HPLC chromatogram of compound **7a**

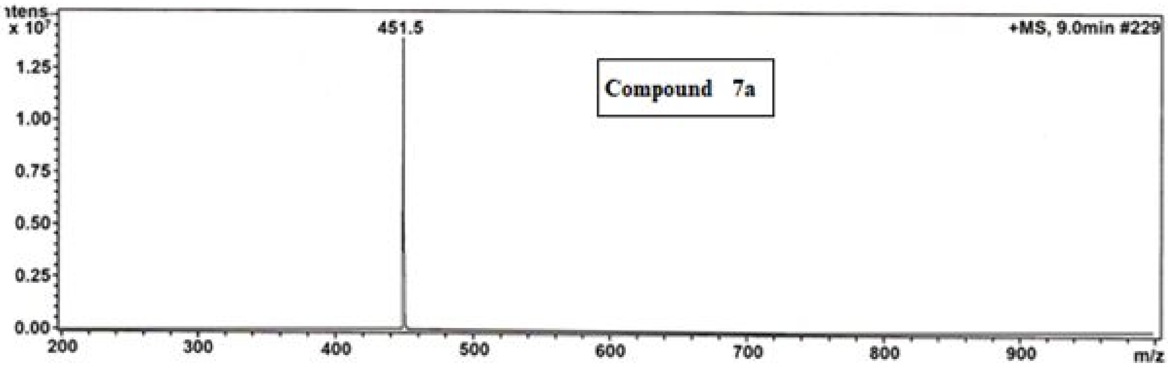

Figure S-19. LC-MS chromatogram of compound **7a**

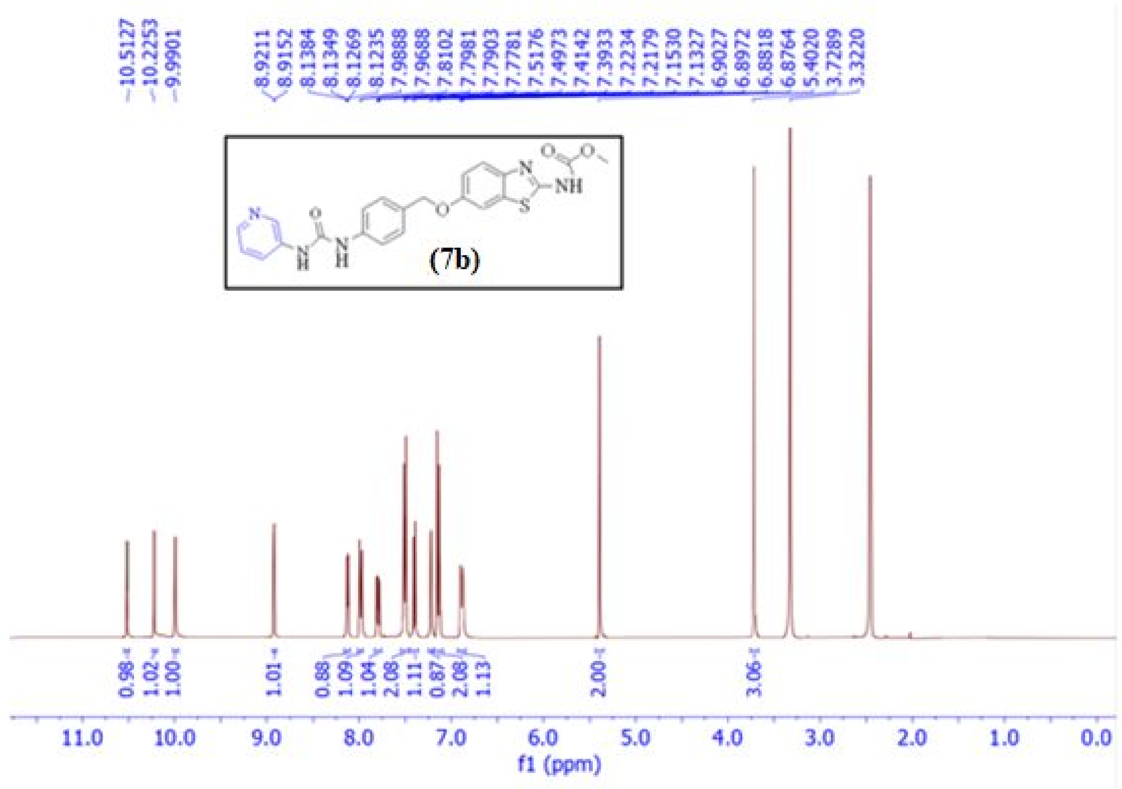

Figure S-20. 1H NMR (400 MHz, DMSO-*d6*) spectrum of compound **7b**

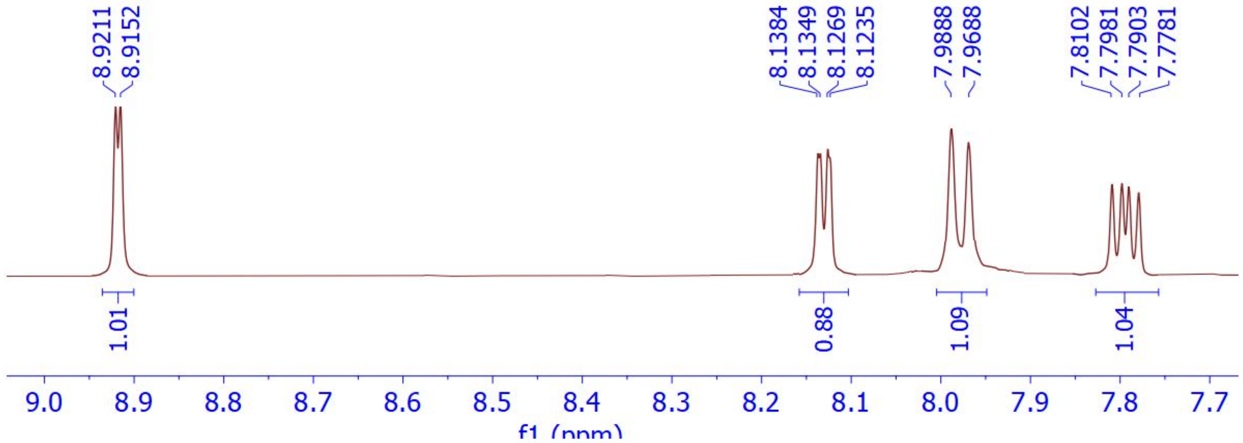

Figure S-21. 1H NMR (400 MHz, DMSO-*d6*) spectrum of compound **7b (**Expansion)

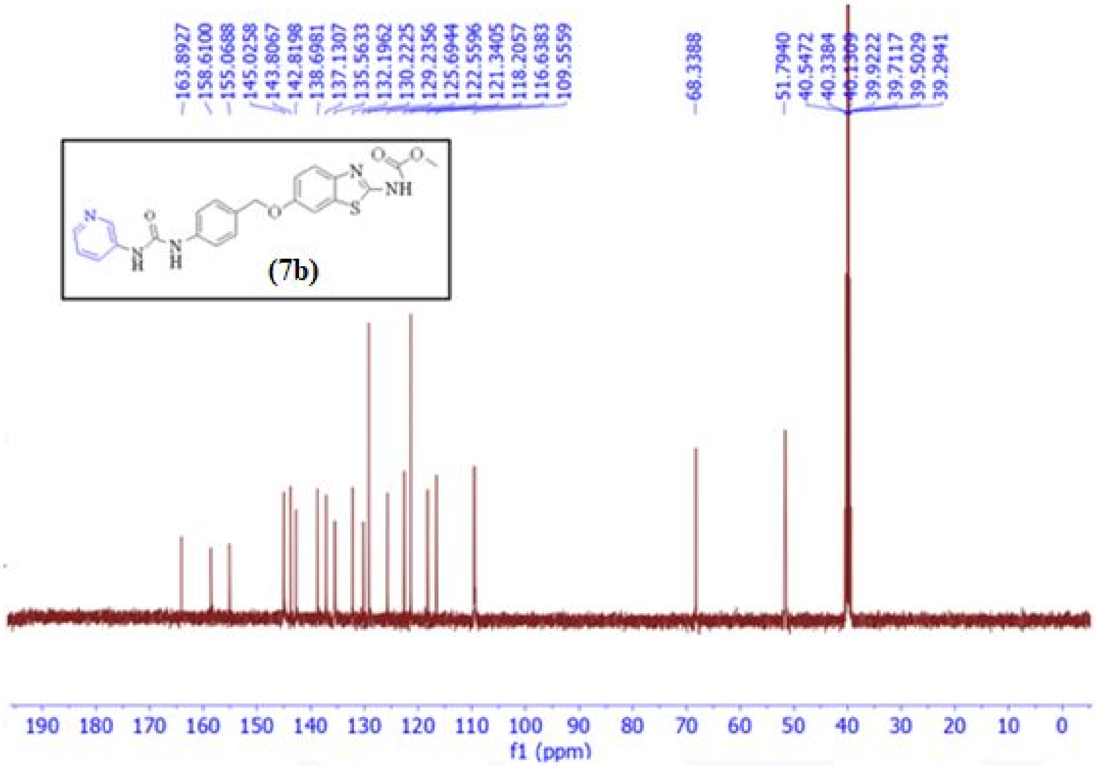

Figure S-22 ^{13}C NMR (100 MHz, DMSO-*d6*) spectrum of compound **7b**

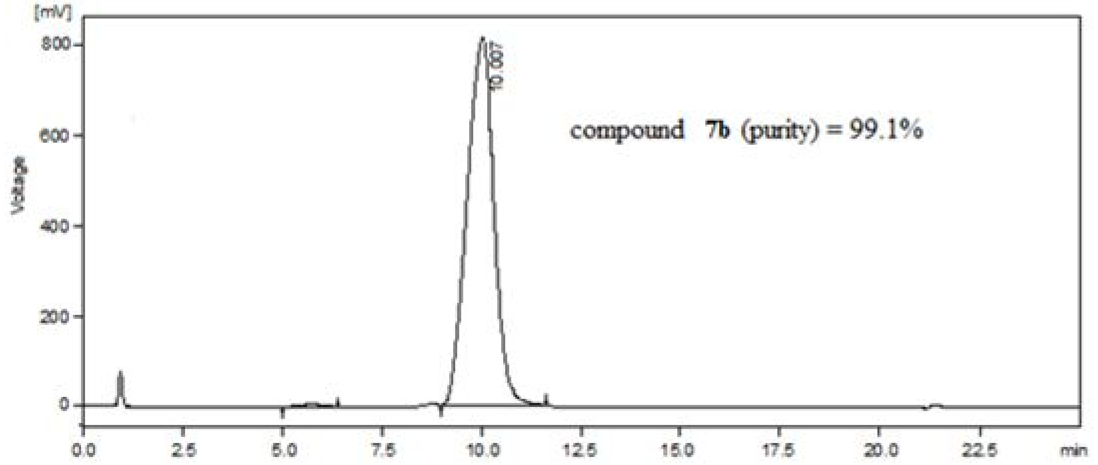

Figure S-23. HPLC chromatogram of compound **7b**

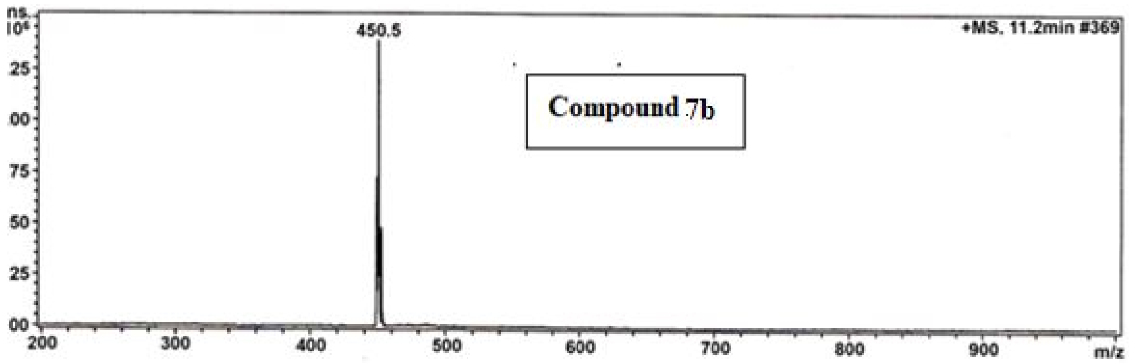

Figure S-24. LC-MS chromatogram of compound **7b**

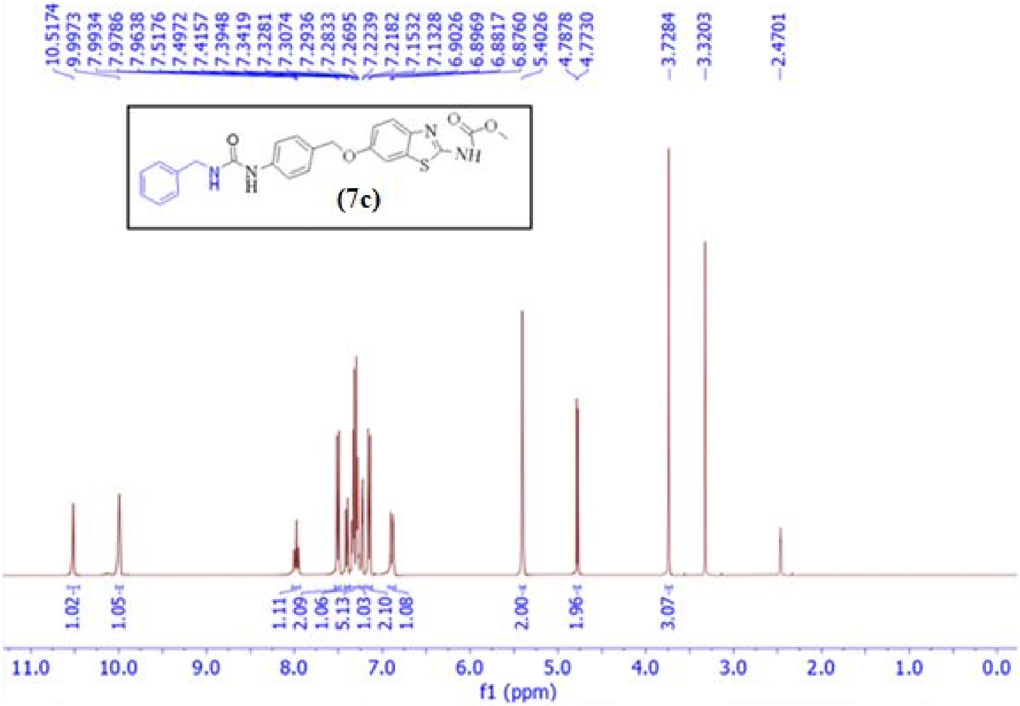

Figure S-25. 1H NMR (400 MHz, DMSO-*d6*) spectrum of compound **7c**

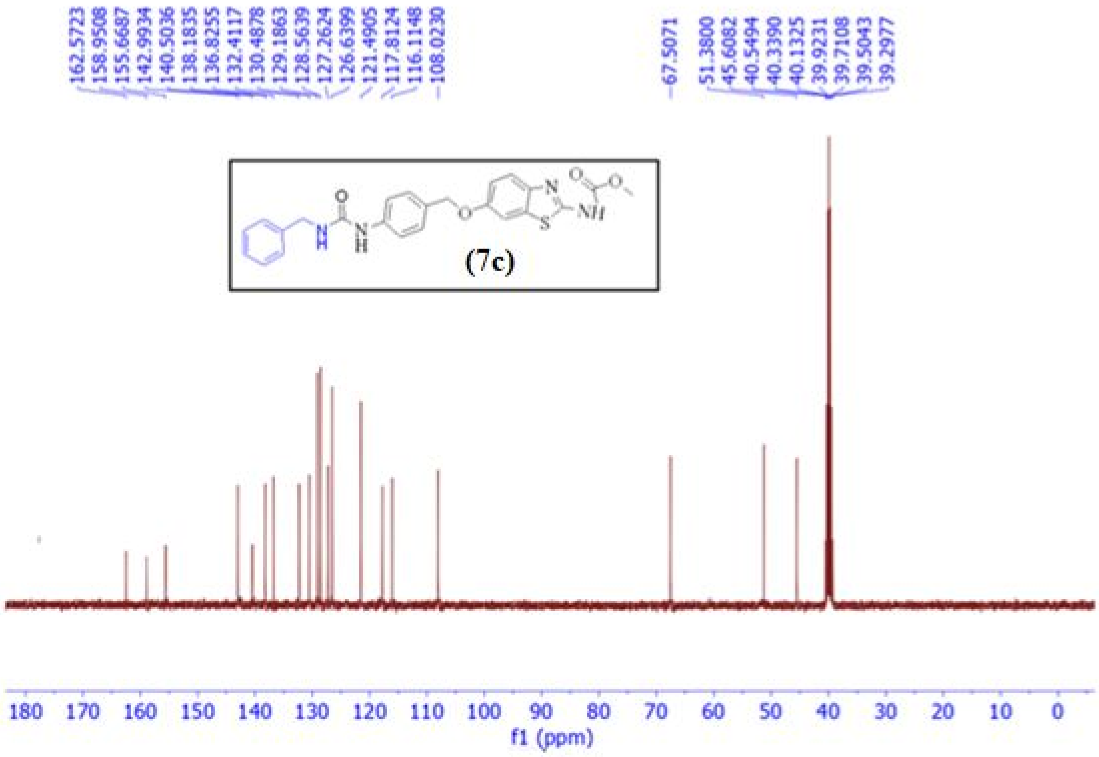

Figure S-26. ^{13}C NMR (100 MHz, DMSO-*d6*) spectrum of compound **7c**

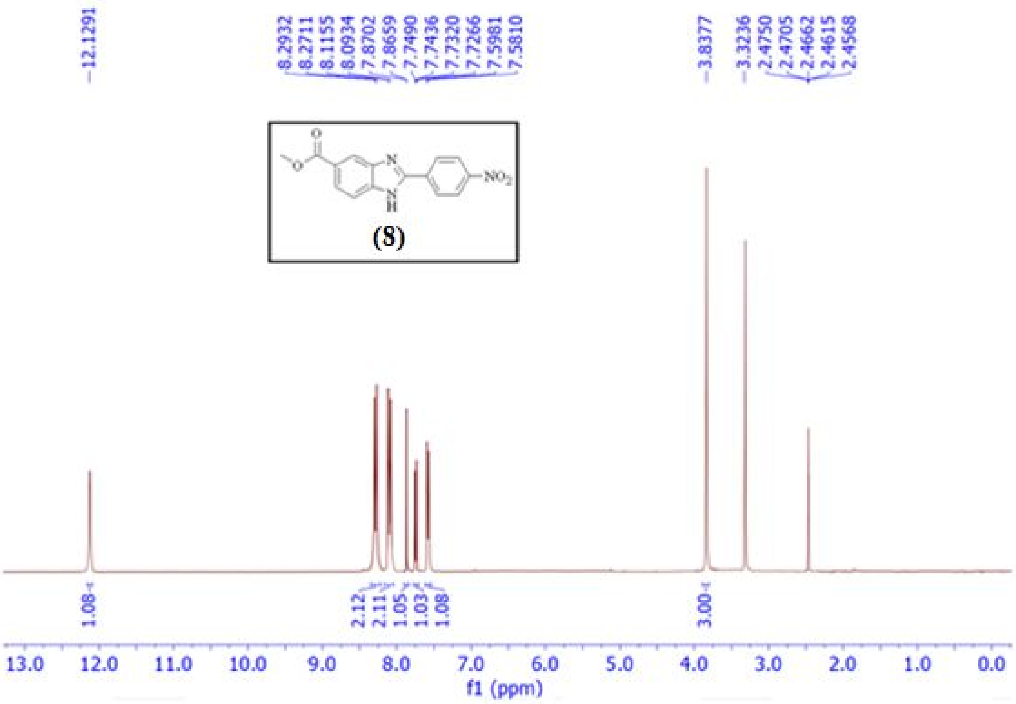

Figure S-27. 1H NMR (400 MHz, DMSO-*d6*) spectrum of compound **8**

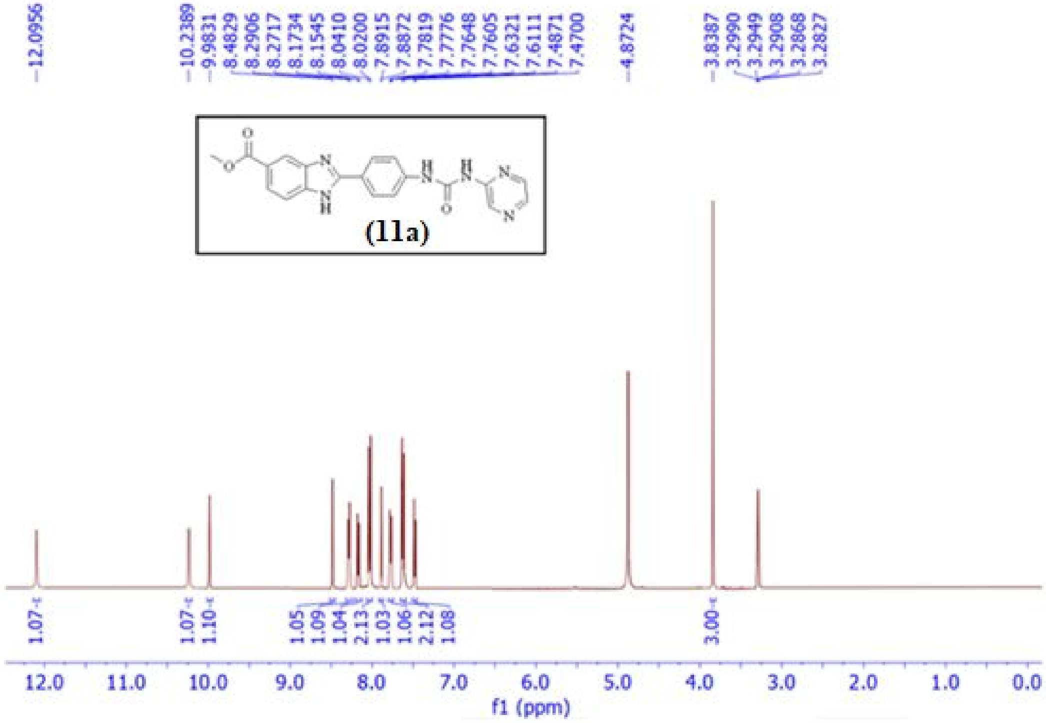

Figure S-28. 1H NMR (400 MHz, MeOD) spectrum of compound **11a**

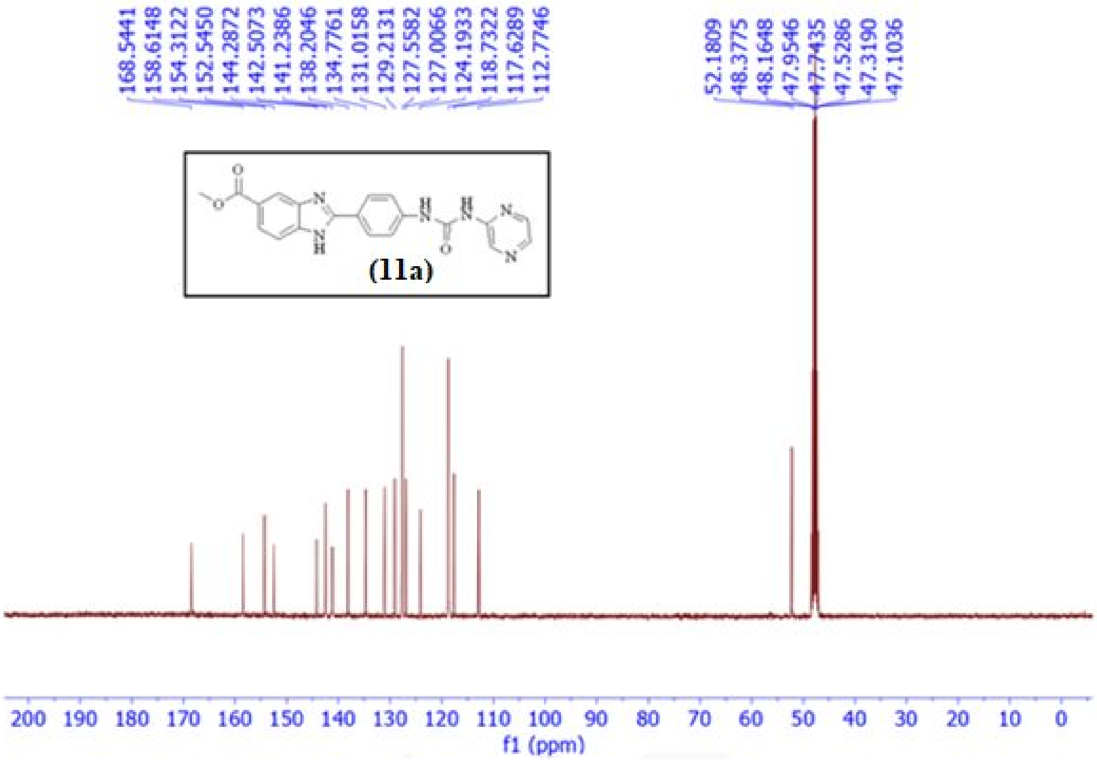

Figure S-29. ^{13}C NMR (100 MHz, MeOD) spectrum of compound **11a**

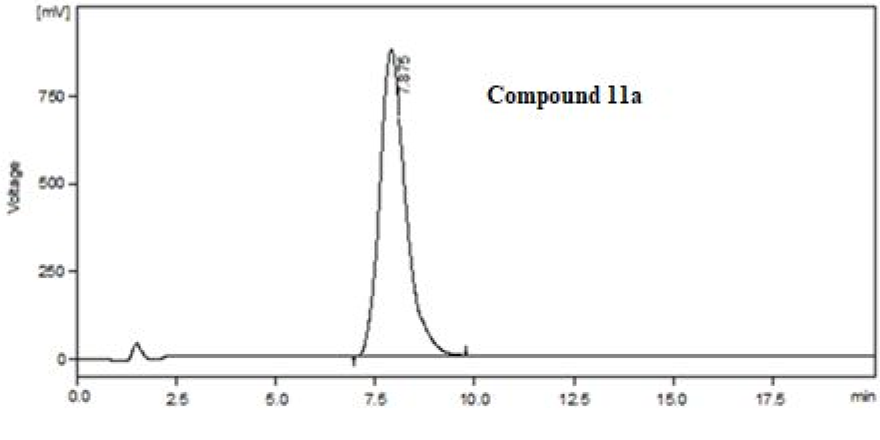

Figure S-30. HPLC chromatogram of compound **11a**

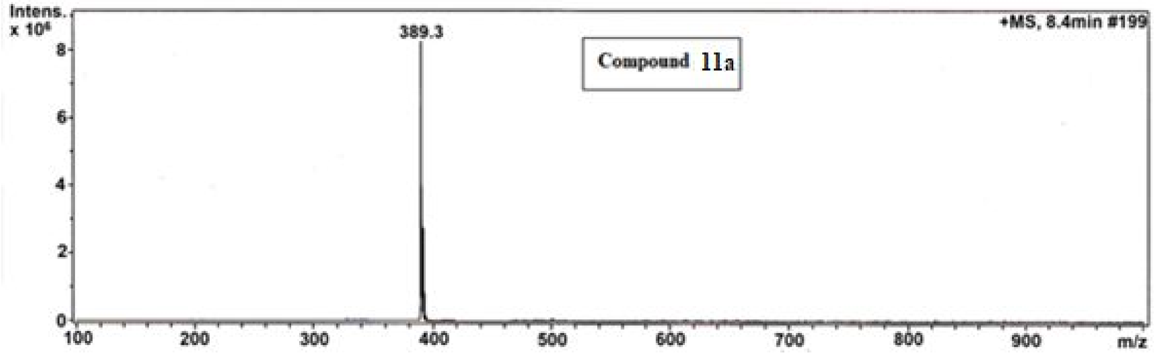

Figure S-31. LC-MS chromatogram of compound **11a**

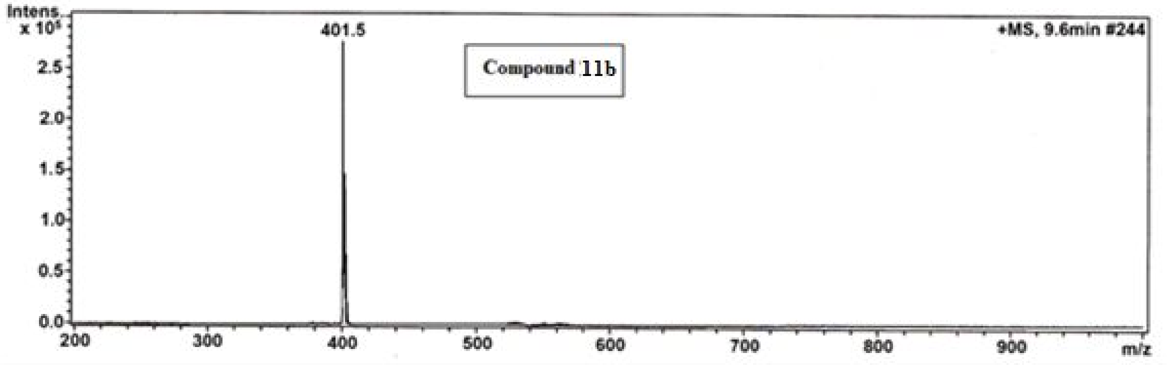

Figure S-32. LC-MS chromatogram of compound **11b**

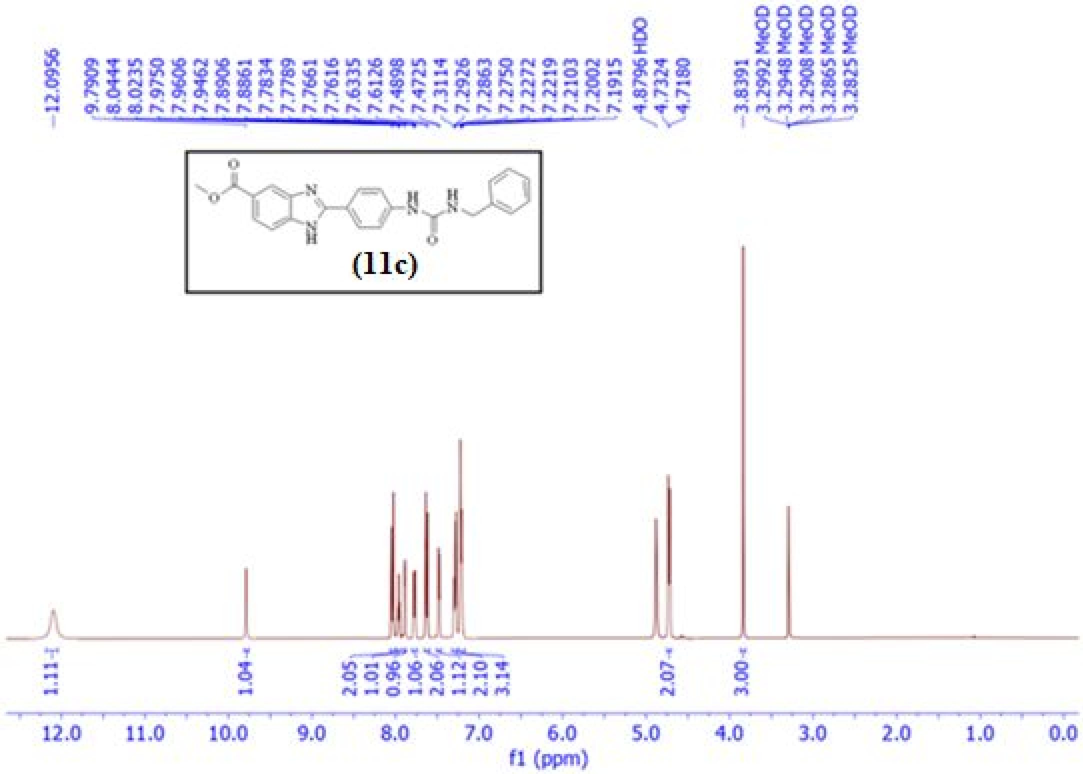

Figure S-33. 1H NMR (400 MHz, MeOD) spectrum of compound **11c**

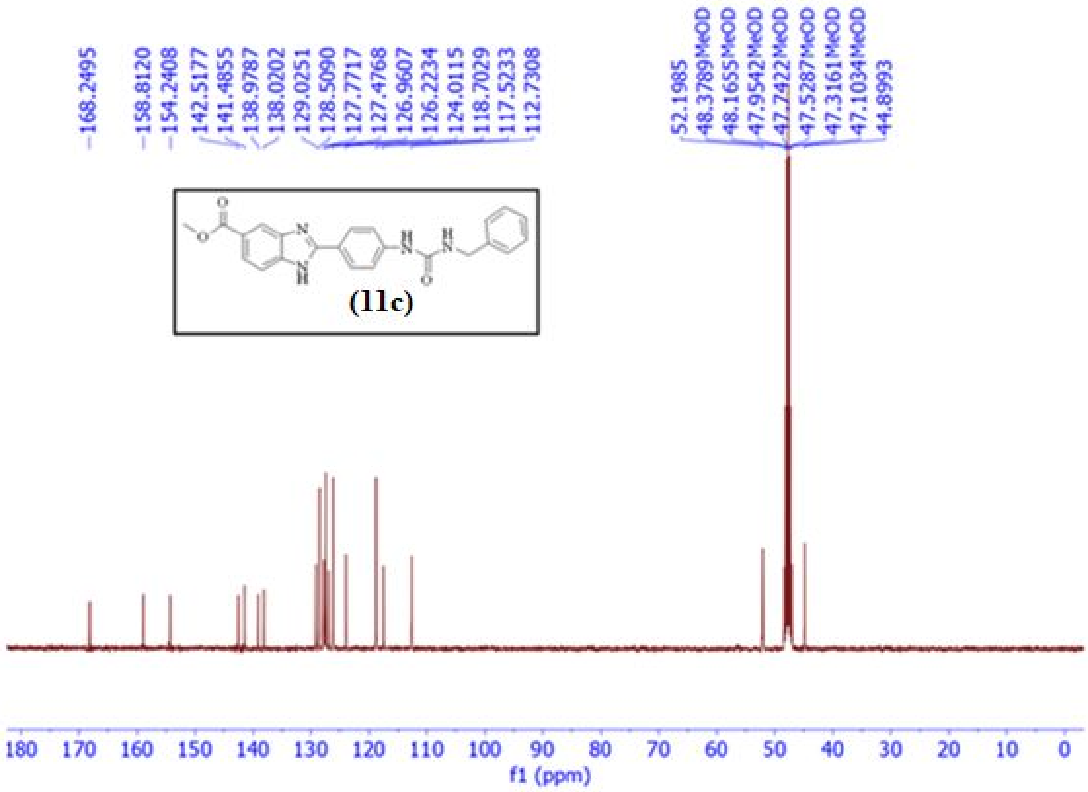

Figure S-34. ^{13}C NMR (400 MHz, MeOD) spectrum of compound **11c**

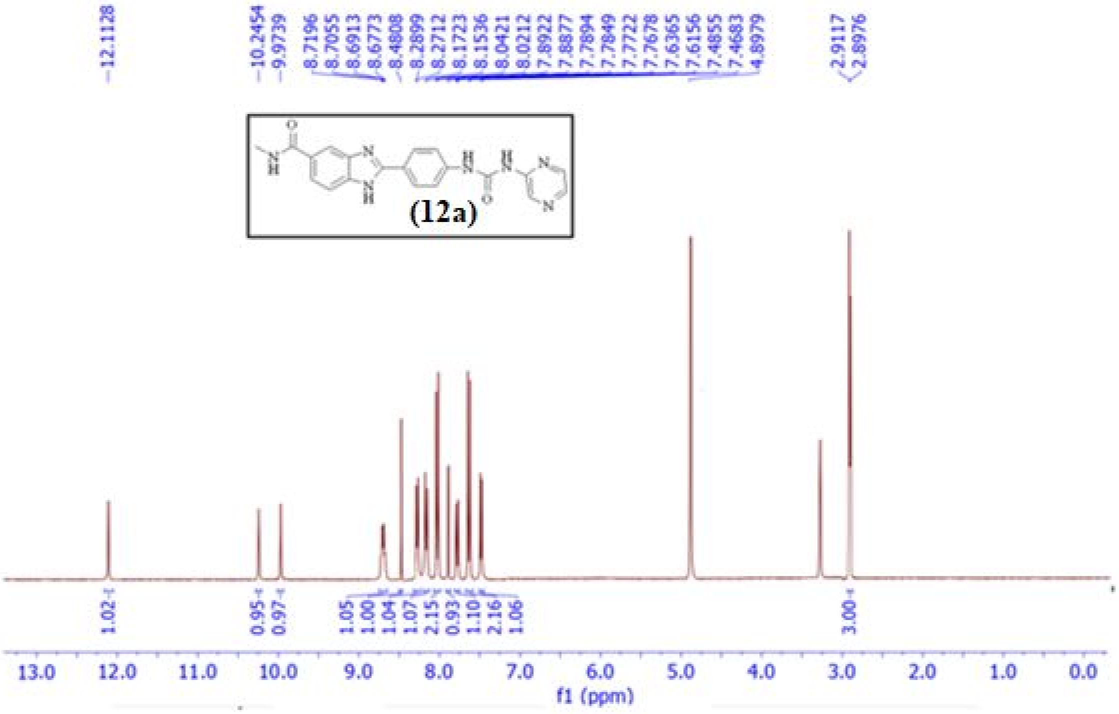

Figure S-35. 1H NMR (400 MHz, MeOD) spectrum of compound **12a**

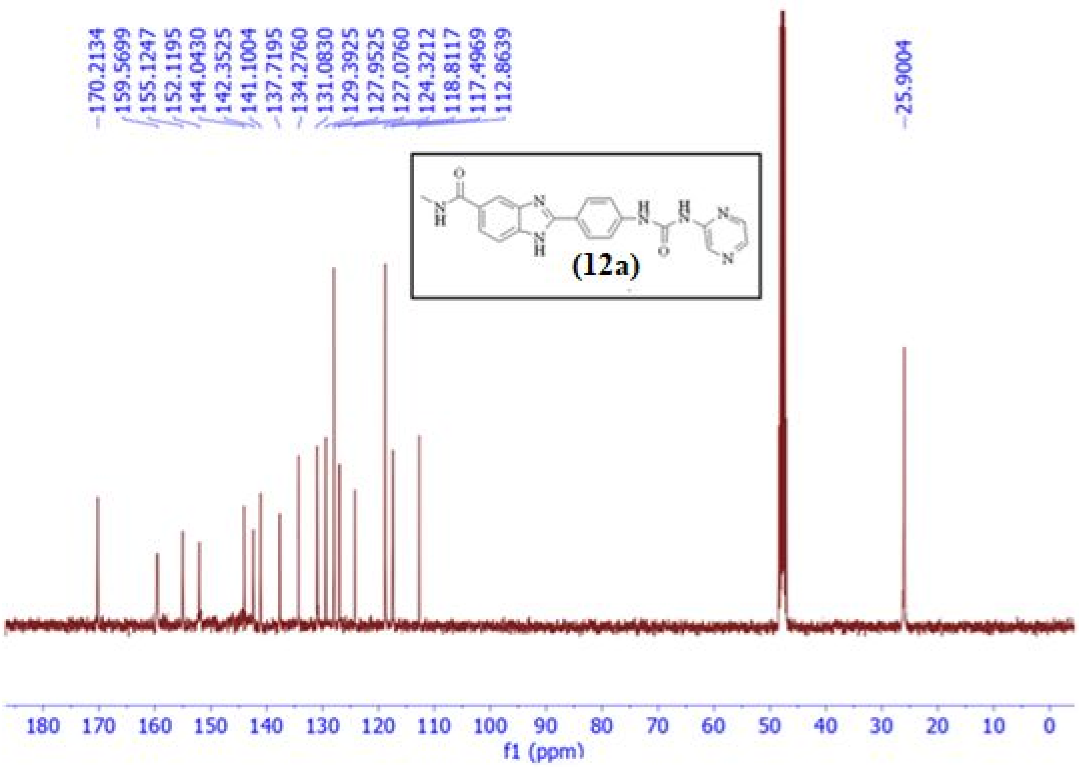

Figure S-36. ^{13}C NMR (400 MHz, MeOD) spectrum of compound **12a**

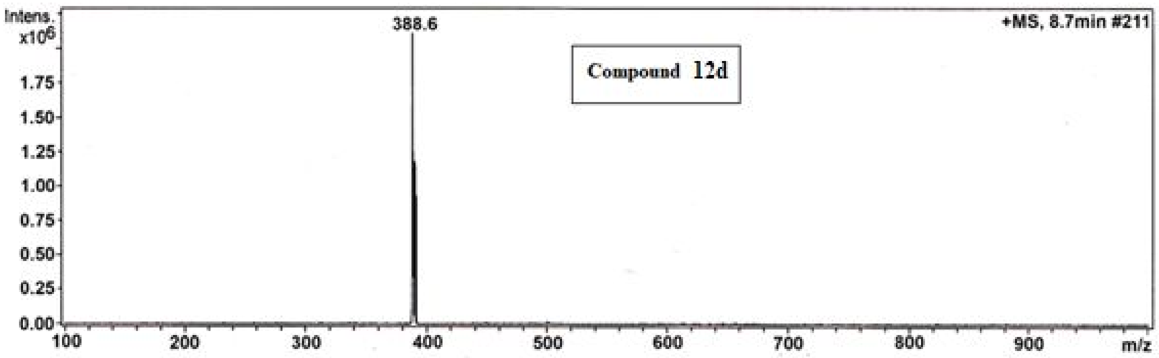

Figure S-37. LC-MS chromatogram of compound **12d**

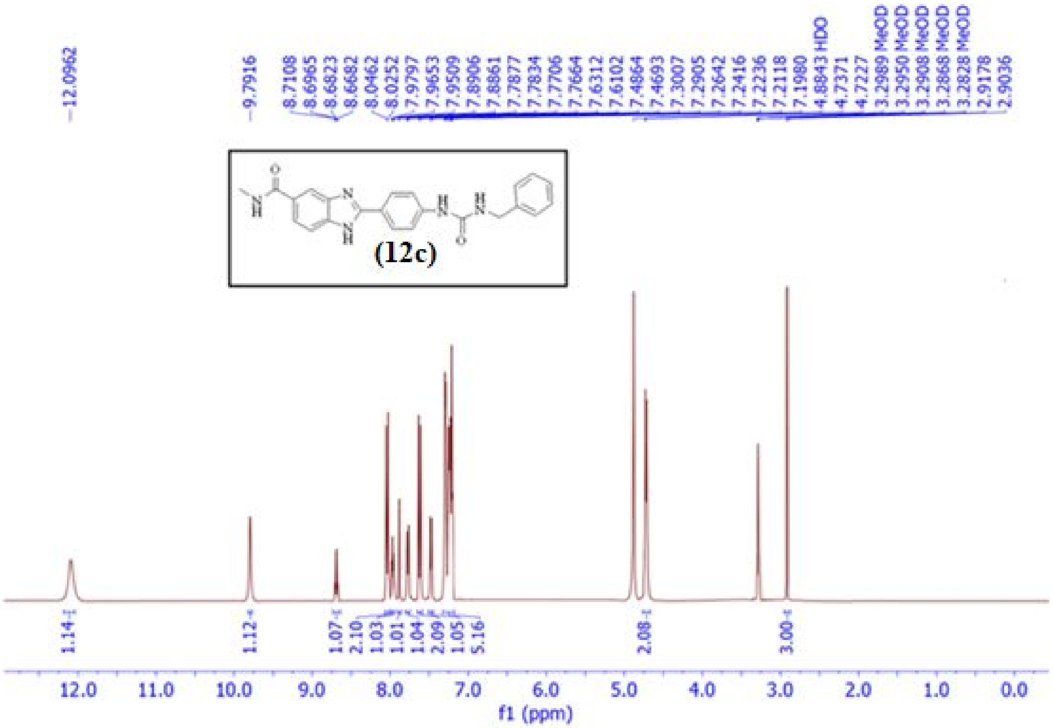

Figure S-38. 1H NMR (400 MHz, MeOD) spectrum of compound **12c**

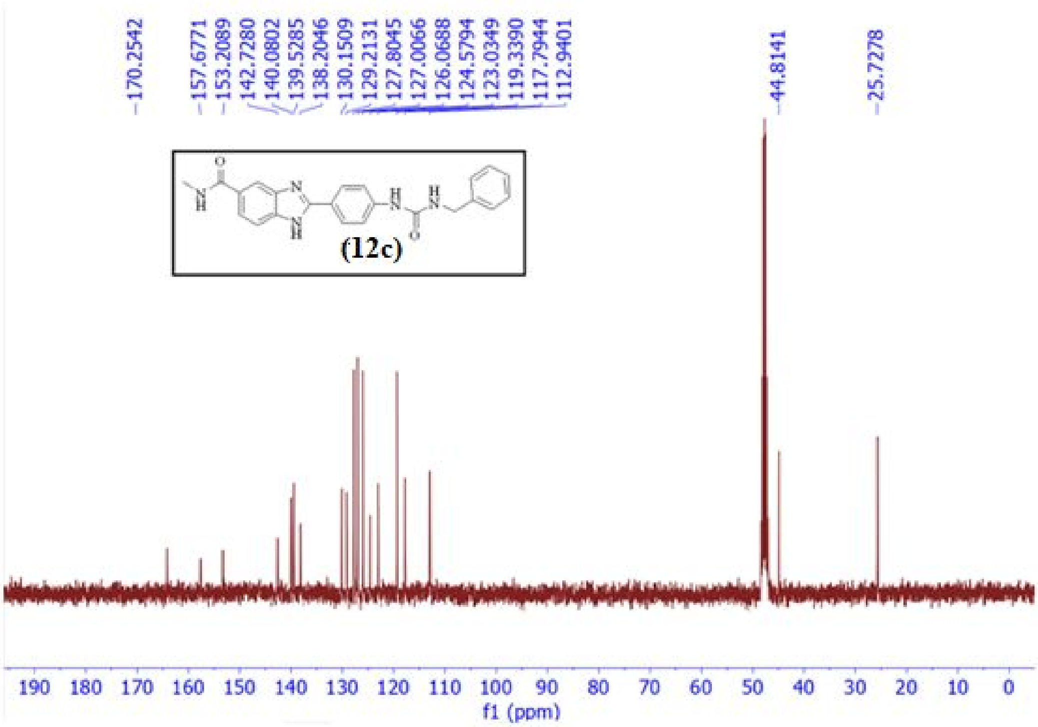

Figure S-39. ^{13}C NMR (400 MHz, MeOD) spectrum of compound **12c**

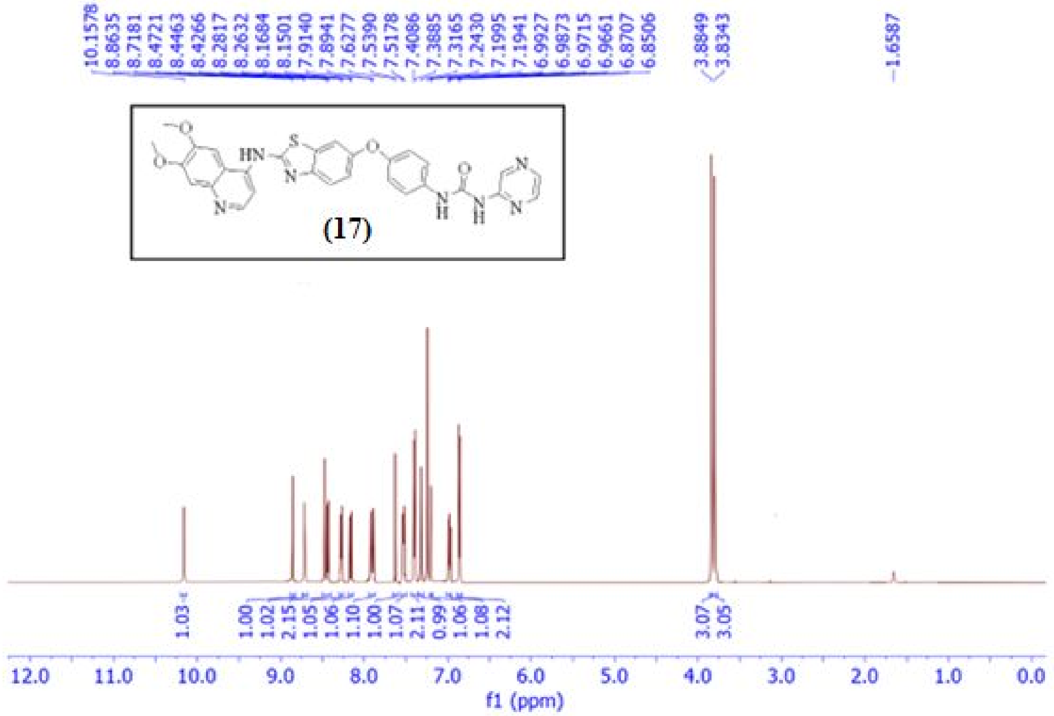

Figure S-40. 1H NMR (400 MHz, $CDCl_3$) spectrum of compound **17**

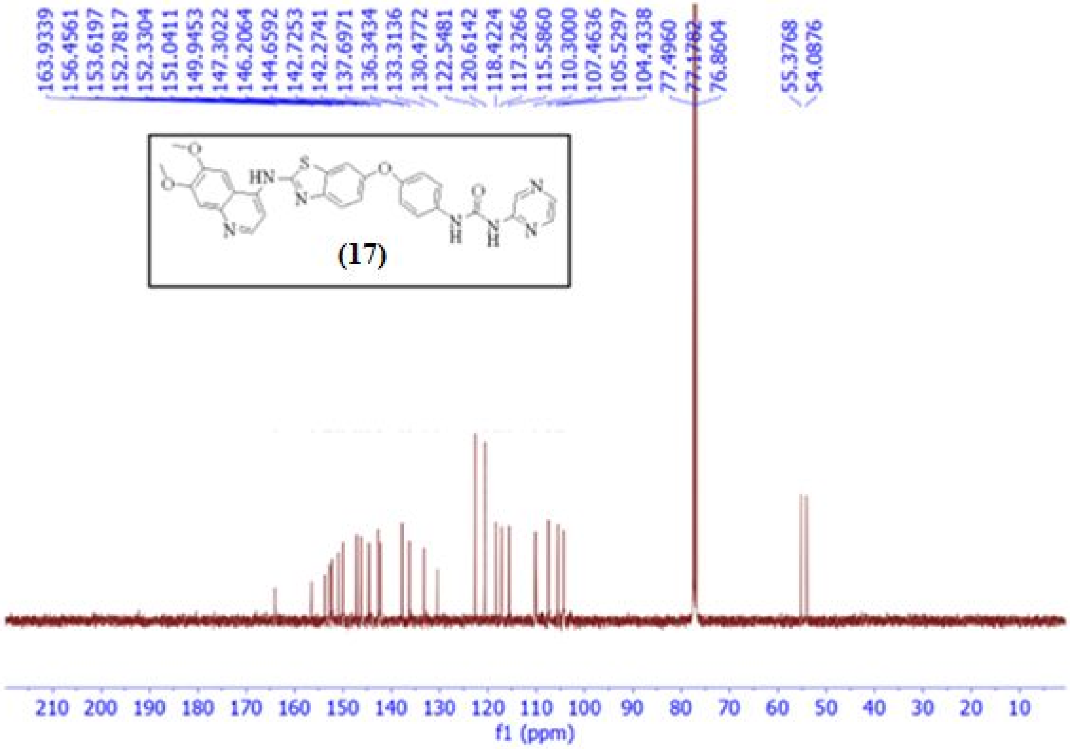

Figure S-41. ^{13}C NMR (400 MHz, $CDCl_3$) spectrum of compound **17**

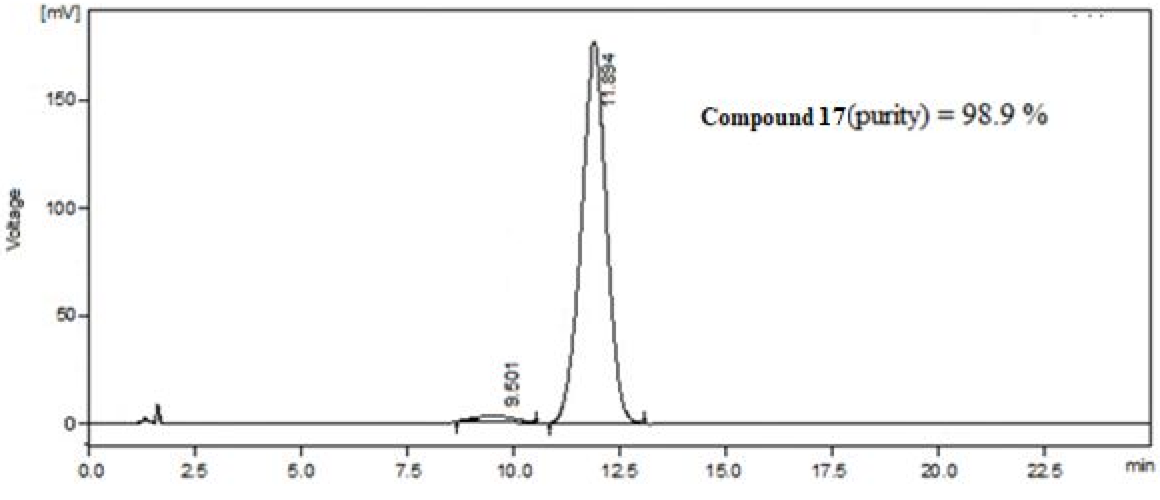

Figure S-42. HPLC chromatogram of compound **17**

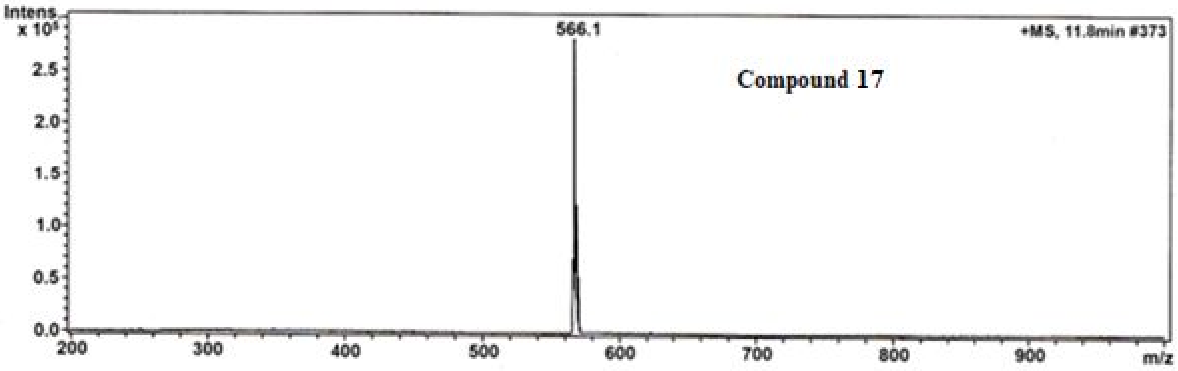

Figure S-43. LC-MS chromatogram of compound **17**

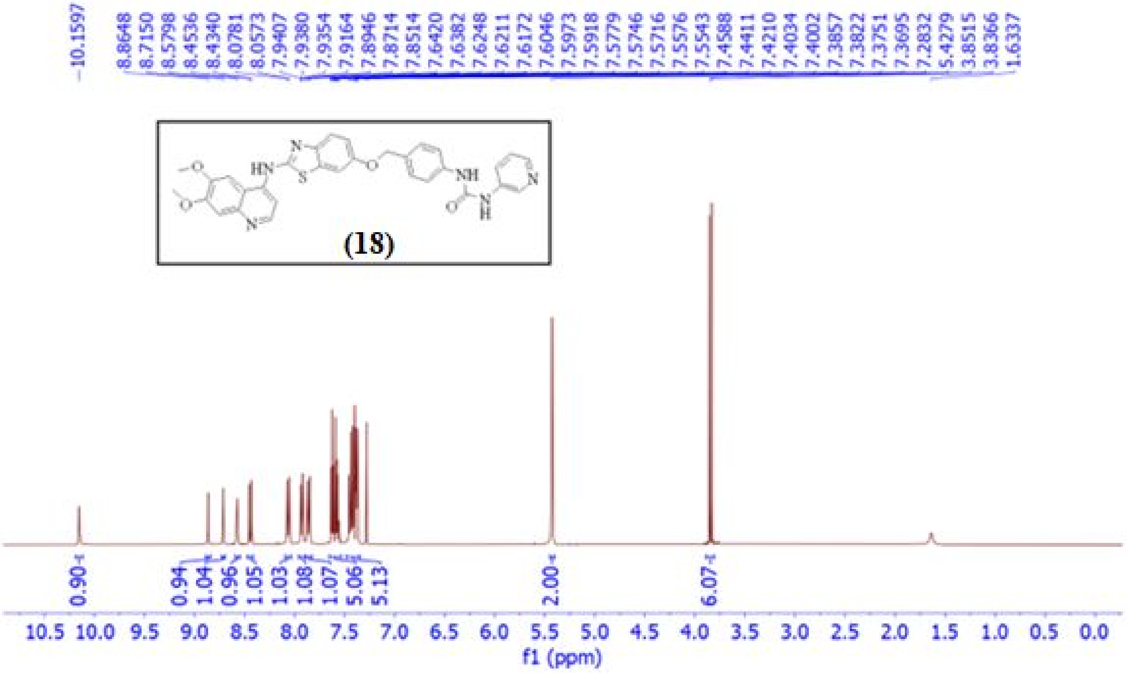

Figure S-44. 1H NMR (400 MHz, DMSO-*d6*) spectrum of compound **18**

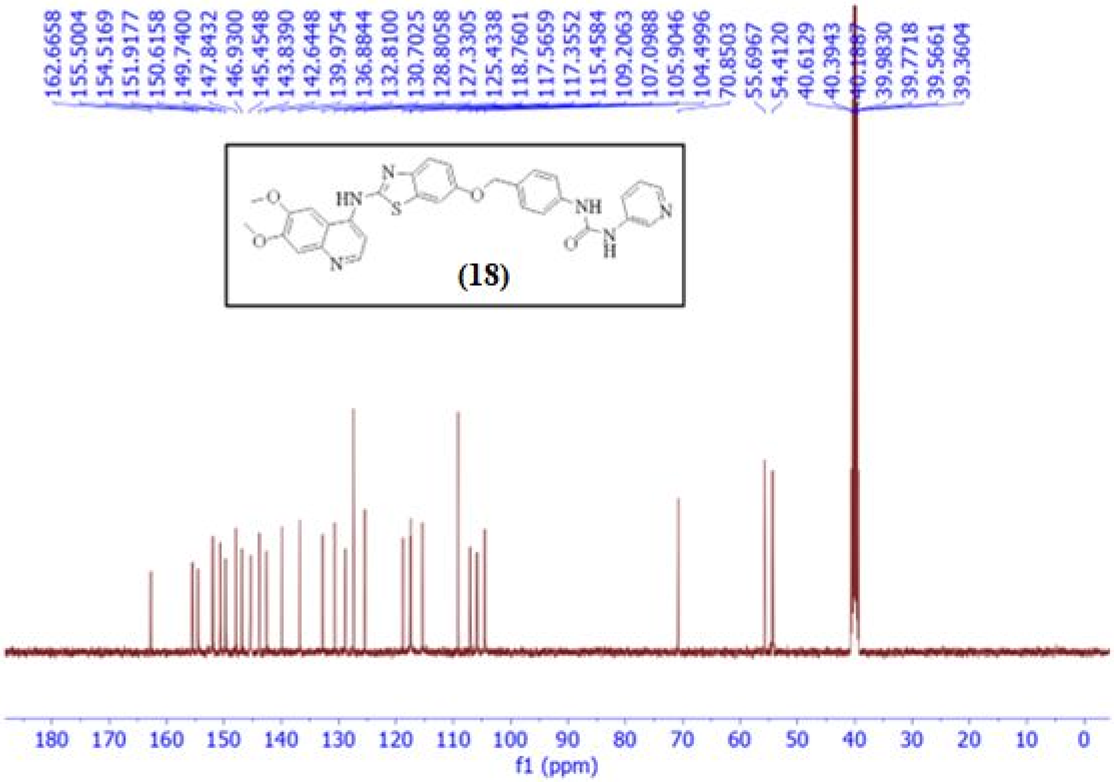

Figure S-45. ^{13}C NMR (400 MHz, DMSO-*d6*) spectrum of compound **18**

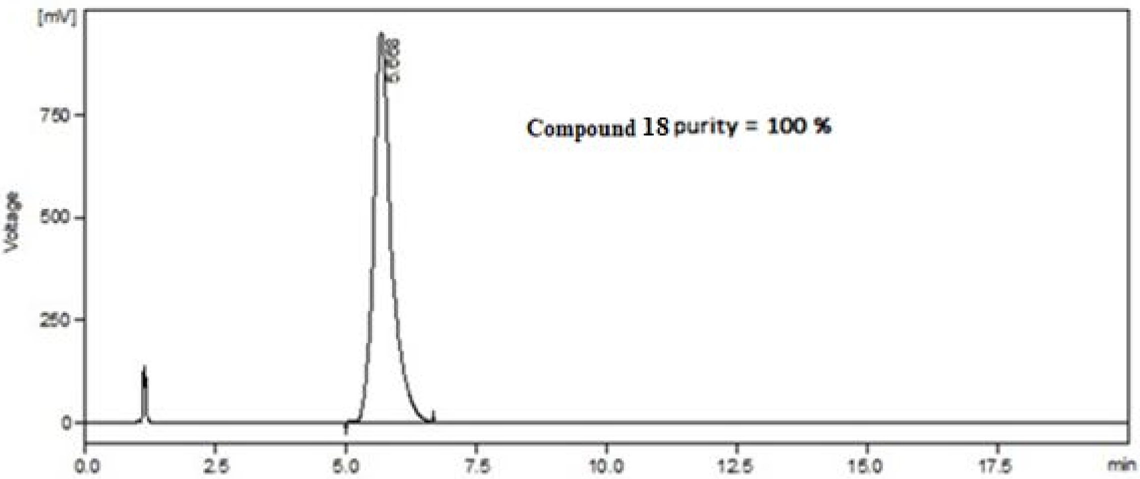

Figure S-46. HPLC chromatogram of compound **18**

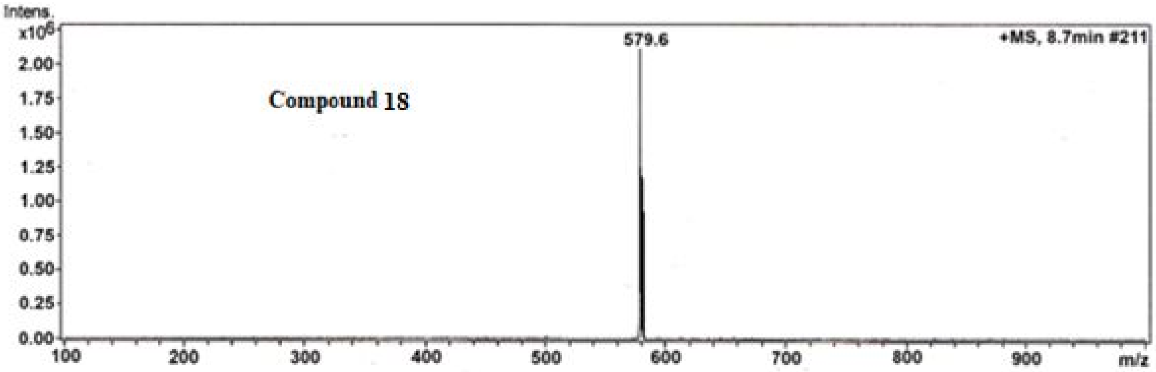

Figure S-47. LC-MS chromatogram of compound **18**

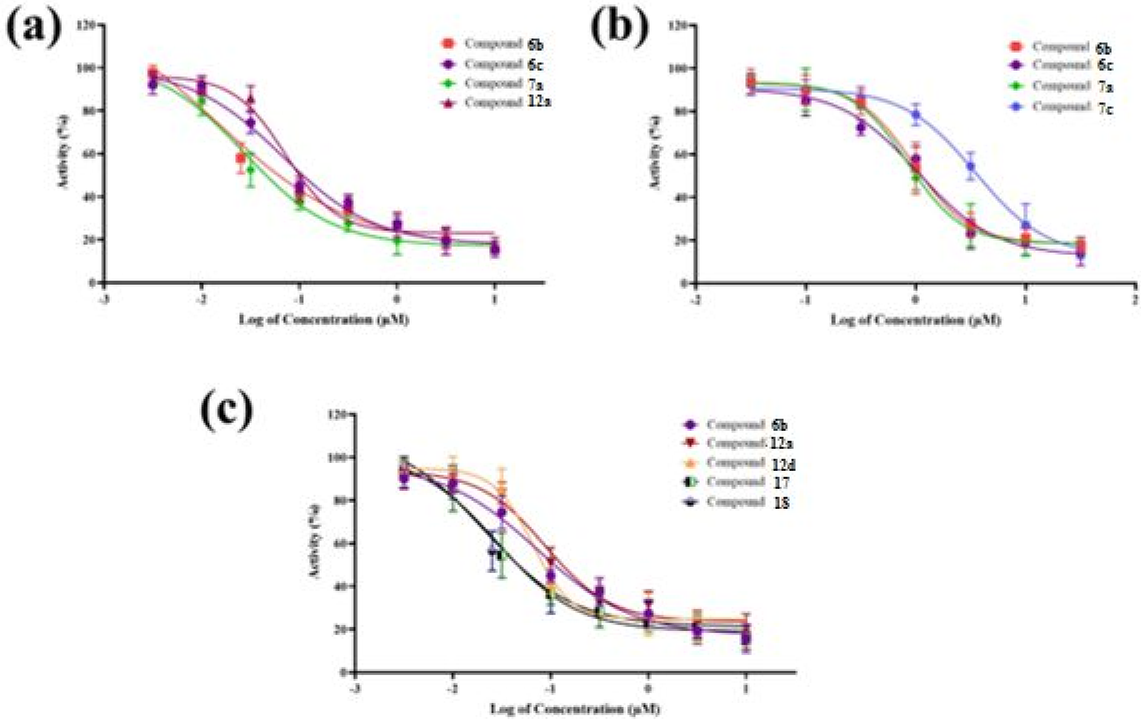

Figure S-48. Dose response curve of (**a**) **6b, 6c, 7a, 12a** against VEGFR-2, (**b**) **6b, 6c, 7a, 7c** against EGFR, and (**c**) **6b, 12a, 12d, 17, 18** against c-Met
